# Photoredox Catalyzed Dealkylative Aromatic Halogen Substitution with Tertiary Amines

**DOI:** 10.3390/molecules26113323

**Published:** 2021-06-01

**Authors:** Dmitry L. Lipilin, Alexander E. Frumkin, Alexey Y. Tyurin, Vitalij V. Levin, Alexander D. Dilman

**Affiliations:** N. D. Zelinsky Institute of Organic Chemistry, Leninsky Prosp. 47, 119991 Moscow, Russia; lipilin@ioc.ac.ru (D.L.L.); frumkin@ioc.ac.ru (A.E.F.); tyurin@ioc.ac.ru (A.Y.T.); levitavl@yandex.ru (V.V.L.)

**Keywords:** aromatic substitution, tertiary amines, photoredox catalysis, radical reactions

## Abstract

A reaction of aromatic halides bearing electron-withdrawing groups with tertiary amines in the presence of an iridium catalyst under blue light irradiation is described. Products of the aromatic substitution of the halide by the dialkylamino fragment are obtained. The interaction of aryl radicals with tertiary amines to generate zwitterionic radical species is believed to be the key factor responsible for the reaction efficiency.

## 1. Introduction

The substitution of halogen by heteroatom-centered nucleophiles in the aromatic ring is an important class of processes leading to numerous industrial products [1,2,3,4]. Since direct nucleophilic substitution proceeds through the loss of aromaticity, these reactions may require harsh conditions. Alternatively, aryl halides may be involved in the transition of metal catalyzed reactions mainly based on the application of complexes of palladium, nickel, or copper [5,6,7,8,9]. In the last decade, photoredox catalysis has emerged as a powerful tool for performing chemical reactions [10,11] and it is also applied for aromatic substitution [12].

**Scheme 1:**
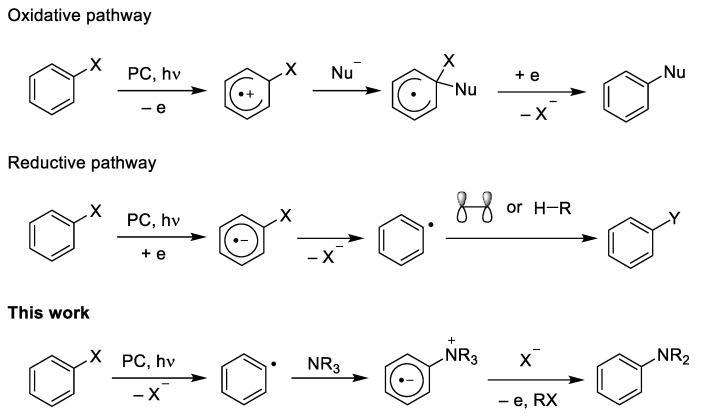
Modes of photocatalytic substitution.

Several types of photoredox mediated substitution reactions were realized differing by the mode of action of the photocatalyst serving either as oxidant or reductant (Scheme 1). The oxidative pathway involves a single electron oxidation of the aromatic substrate by the light activated catalyst followed by nucleophilic attack and subsequent reduction [13,14,15,16,17]. This pathway works well for aromatics bearing electron donating groups. Another mode involves the application of strongly reducing catalysts, which can reduce the aromatic halide to generate aryl radical capable of interacting with a nucleophile with subsequent oxidation [18,19]. Reactions may also be performed via dual activation involving both photoredox and transition metal catalysis [20,21,22,23].

The application of reductive photocatalysts toward generation of radicals is most appropriate for electron deficient substrates such as fluorinated or heteroaromatic compounds [24]. However, the resulting aryl radicals are typically trapped by π-systems (alkenes, alkynes, electron rich aromatics) or abstract a hydrogen atom from a suitable reagent [24,25,26,27,28].

Herein, we report a substitution of aromatic halides by dialkylamine group in reaction with *tertiary amines*. The reaction proceeds via the photoredox generation of the aryl radical, which interacts with the amine followed by loss of electron and elimination of one group from the ammonium fragment. Classical uncatalyzed nucleophilic substitution reactions of chlorine or heavier halogens with tertiary amines are known but proceed under thermal conditions [29,30,31,32], while such reactions of fluorides have not been described.

## 2. Results and Discussion

2,4-Dinitrofluorobenzene (**1a**) and triethylamine were selected as model compounds and their reaction was evaluated (Table 1). In dimethylsulfoxide, in the absence of photocatalyst or in the dark, there was no product formation at room temperature within 30 h. Traces of amine **3a** containing diethylamino fragment were observed when the reaction was heated at 50 °C for more than two days. Irradiation with blue LED at room temperature led to the 10% conversion (entry 3). Rewardingly, the use of photocatalysts gave noticeable improvement, with strongly reductive ones being more efficient. The best result was achieved using *fac*-Ir(ppy)_3_ affording product **3a** in 84% isolated yield (entry 8). Though incomplete conversion of **1a** was observed, increase of the reaction time did not give the increase of the yield of **3a**. Other solvents were evaluated but gave inferior results (entries 10–12).

Under the above conditions, a series of tertiary amines were involved in the reaction with 2,4-dinitrofluorobenzene leading to products of the substitution of the fluoride by the dialkylamino group (Table 2). To reduce the amount of amine to 1.1 equiv, Hünig base (1 equiv) was added, which does not react with the substrate due to steric reasons but serves as a basic scavenger. Amines bearing non-identical substituents may afford various products. In the reaction of cyclic *N*-methylamines, the methyl group was selectively removed (products **3c**–**f**). The reaction of 2-optimized cyanoethyldimethylamine unexpectedly furnished dimethyl-substituted amine **3g** as a single product. The preferred removal of the 2-cyanoethyl compared to the methyl group may be associated with the presence of acidic proton at the α-position of the nitrile, thereby favoring E2 elimination with the formation of acrylonitrile. In case of unsymmetrical alkyldimethylamines, the methyl group is cleaved preferentially, though traces of the products arising from the removal of another alkyl group were formed, and the portion of the latter products increases along with the expected efficiency of the nucleophilic substitution at carbon connected to nitrogen. These data allow to propose the following row of substituents with respect to increasing propensity of being detached from the amine: Cy < *i*-Pr < primary alkyl < Bn < allyl.

**Scheme 2:**
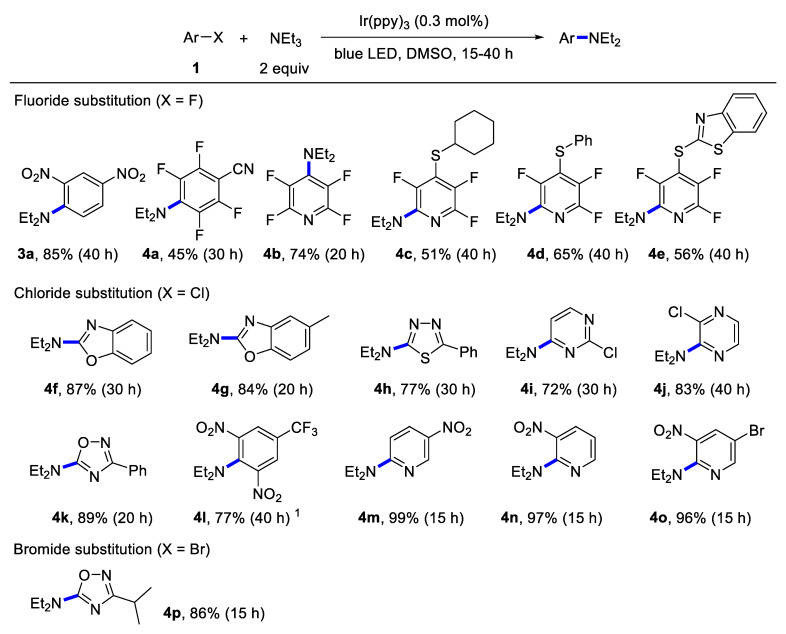
Reaction of aryl halides with NEt_3_. Isolated yields are shown, reaction times are in parenthesis. ^1^ Performed in CH_2_Cl_2_.

Then, reactions of various aromatic substrates **1** with 2 equiv of triethylamine were evaluated (Scheme 2). The fluoride can be displaced starting from pentafluoropyridine and 4-thio-substituted tetrafluorinated pyridines [33,34]. The reaction worked well with a series of heteroaromatic chlorides. Derivatives of benzoxazole, oxadiazole, and diazines gave good yields. At the same time, 2-chlorobenzothiazole, 1-chloro-2,4,6-trinitrobenzene, 4-nitrofluorobenzene, 4-bromofluorobenzne, ethyl 4-fluorobenzoate were unreactive.

**Scheme 3:**
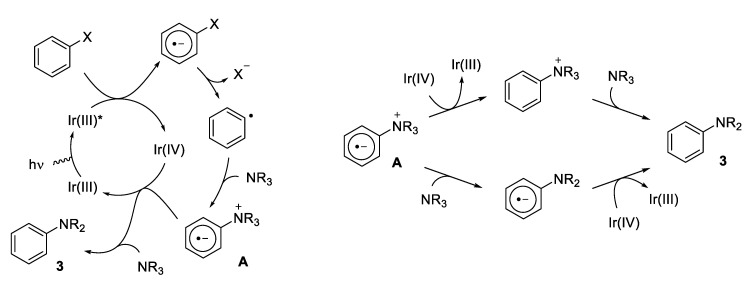
Proposed mechanism.

The proposed mechanism is shown in Scheme 3. Starting aryl halide is reduced by the photoexcited iridium complex with elimination of the halide anion and generation of the aryl radical. The radical interacts with tertiary amine leading to zwitterionic species **A**, which is converted into the product **3** along with regeneration of the Ir(III) complex. The transformation of intermediate **A** into the product involves single electron oxidation of the aromatic π-system and removal of the alkyl group from nitrogen with the aid of the amine (Scheme 3, right). The detachment of the alkyl group may be realized either via nucleophilic substitution or through E2 elimination mechanism, especially if the group has acidic hydrogen at the β-position. The order of oxidation and alkyl group removal may depend on the nature of substituents in the aromatic ring and at the nitrogen atom. The radical character of the process was supported by an experiment with a radical trap. Thus, when the reaction of 2,4-dinitrofluorobenzene with triethylamine were carried out in the presence of TEMPO (1.5 equiv) under standard conditions, no product was formed, leaving the starting arylfluoride unconsumed.

The redox potential of 2,4-dinitrofluorobenzene (**1a**) in DMSO was measured by cyclic voltammetry affording a value of −0.93 V (vs. SCE) (see Supplementary Materials for CV curves). The photoexcited *fac*-Ir(ppy)_3_ has the potential of −1.73 V (from Ir(III)* to Ir(IV)) [10], thereby suggesting facile single electron reduction of **1a**. It should also be pointed out that the photoexcited catalyst may be reductively quenched by tertiary amine generating Ir(II), which has even stronger reducing power (for Ir(II)/Ir(III), −2.19 V [10]). The use of iridium photocatalysts for the generation of aryl radicals by reductive cleavage of the C-F bond in aromatic aryl fluorides was described [24].

**Scheme 4:**
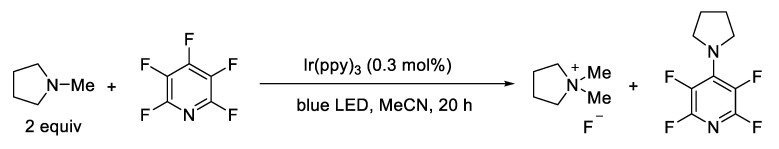
Formation of ammonium salt.

To gain insight into the alkyl detachment step from intermediate **A**, a photoreaction of pentafluoropyridine with two equivalents of *N*-methylpyrrolidine was evaluated (Scheme 4). The reaction was performed in acetonitrile, and after completion the mixture was directly analyzed by ^1^H, ^13^C, ^19^F NMR, and no alkyl fluorides were observed. The acetonitrile was evaporated and the residue was washed to remove all non-ionic organic compounds. When the residue was analyzed by NMR spectroscopy in DMSO-d_6_, *N,N*-dimethylpyrrolidinium cation was observed (the reference ammonium cation was formed by interaction of *N*-methylpyrrolidine with methyl iodide). The combined methyl *tert*-butyl ether phases were analyzed by GC-MS demonstrating that tetrafluorinated 4-pyrrolydinopyridine is a single substitution product.

**Figure 1:**
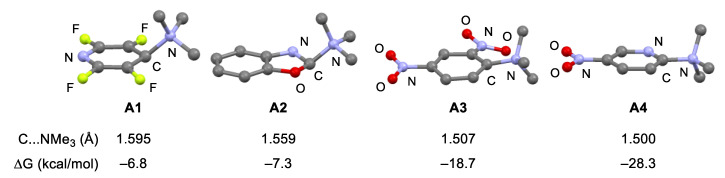
Calculated structures of complexes **A** formed upon interaction of aryl radicals with NMe_3_ (UM06-2X/6-31+G(d), DMSO as solvent). Hydrogen atoms are omitted. Bond length between the carbon atom of the aromatic ring and the nitrogen and free energies of complex formation are shown.

To support the hypothesis of the formation of species **A**, the free energies of the interaction of aryl radicals with trimethylamine were derived by means of quantum chemical calculations. Energies were calculated at M06-2X/6-31+G(d) [35], and for the stationary points, energies were calculated by CPCM method in DMSO solution (Figure 1). In case of perfluorinated pyridinyl and benzoxazolyl radicals, in the resulting complexes **A1** and **A2**, the amine nitrogen is located out of plane of the heteroaromatic ring having relatively long C,N bond of 1.595 Å and 1.559 Å, respectively. Such a geometry can be considered as a weak Meisenheimer complex between the amine and the aromatic system. On the other hand, for the complexes originated from 2,4-dinitrophenyl and 3-nitropyridinyl radicals, the amine fragment is located within the plane of the aromatic ring, with the C,N bonds in **A3** and **A4** being equal to 1.507 Å and 1.500 Å, respectively. Of special note, for the latter complexes, the free energies of their formation are notably more negative compared to those of **A1** and **A2**.

## 3. Materials and Methods

### 3.1. General Information

All reactions were performed under an argon atmosphere. DMSO was distilled from CaH_2_ and stored over MS 4Å. Column chromatography was carried out employing silica gel (230–400 mesh). High resolution mass-spectra (HRMS) were measured using electrospray ionization (ESI) and a time-of-flight (TOF) mass analyzer (Bruker MicrOTOF II, Bruker, Billerica, USA). The measurements were done in a positive-ion mode (interface capillary voltage −4500 V) or in a negative-ion mode (3200 V); the mass ranged from m/z 50 to m/z 3000. For irradiation, a strip of 455 nm light-emitting diodes (SMD 2835–120 LED 1 M Blue, 12 V, 24 W/m; 70 cm strip length) was used. The distance between the reaction vessel and diodes was about 30 mm, and the reaction vial was cooled with a fan.

4-(Cyclohexylthio)-2,3,5,6-tetrafluoropyridine [34] and 2,3,5,6-tetrafluoro-4-(phenylthio)pyridine [36] were obtained according to literature procedures.

*2-[(2,3,5,6-Tetrafluoropyridin-4-yl)sulfanyl]-1,3-benzothiazole.* Potassium carbonate (1.74 g, 12.8 mmol) was added to a solution of 2-mercaptobenzothiazole (2.1 g, 12.6 mmol) in DMF (15 mL). The mixture was stirred for 15 min, then added pentafluoropyridine (2.3 g, 13.8 mmol), and the mixture was stirred for one hour at room temperature. The mixture was poured into water (100 mL), the precipitate was filtered, washed with water, and dried in air. The obtained material was purified vie short silica gel column eluting with dichloromethane. Yield 3.2 g (80%). Colorless solid. Mp 69–71 °C (EtOH). ^1^H NMR (300 MHz, DMSO-d_6_) δ 8.10 (d, *J* = 7.4 Hz, 1H), 7.92 (d, *J* = 7.6 Hz, 1H), 7.47–7.53 (m, 2H). ^13^C NMR (75 MHz, DMSO-d_6_) δ 149.4.7, 152.6, 143.4 (dm, *J* = 239 Hz), 142.5 (dm *J* = 258 Hz), 136.3, 127.2, 126.1, 125.2 (m), 122.6, 122.5. ^19^F NMR (282 MHz, DMSO-d_6_) δ −90.8 (d, *J* = 7 Hz), −134.1 (d, *J* = 7 Hz). HRMS (ESI): calcd for C_12_H_5_F_4_N_2_S_2_, 316.9825 [M + H]; found, 316.9814.

### 3.2. Reactions of Aryl Halides with Amines

#### 3.2.1. Reaction of 2,4-Dinitrofluorobenzene with Tertiary Amines. Synthesis of Amines 3. General Procedure 1 (GP 1)

*fac*-Ir(ppy)_3_ (2 mg, 0.003 mmol), EtN(*i*-Pr)_2_ (174 μL, 1 mmol) and amine (1.1 mmol) were added to a solution of 2,4-dinitrofluorobenezene (186 mg, 1 mmol) in DMSO (2 mL). The mixture was irradiated at room temperature with blue LED [for **3c,g,j,n**, 30 h; for **3a,b,d**–**f,h,i,k,m**, 40 h; for **3l,o,p**, 50 h]. For the work-up, the mixture was poured into 2% aqueous hydrochloric acid (20 mL) and extracted with EtOAc (4 × 15 mL). The combined organic phases were washed with water (10 mL), a 5% solution of potassium carbonate (10 mL), brine (10 mL), and dried over Na_2_SO_4_. The solvent was evaporated, and the residue was purified by column chromatography eluting with dichloromethane.

#### 3.2.2. Reactions of Aryl Halides with Tertiary Amines. Synthesis of Amines 4. General Procedure 2 (GP 2)

*fac*-Ir(ppy)_3_ (2 mg, 0.003 mmol) and NEt_3_ (282 μL, 2 mmol) were added to a solution of aromatic halide (1 mmol) in DMSO (2 mL). The mixture was irradiated at room temperature with blue LED for 15–40 h (the reaction time is shown in Scheme 3). The work-up is the same as in General Procedure 1.

*N,N-Diethyl-2,4-dinitroaniline* (**3a**) [37]. According to GP 1; yield 201 mg (84%). According to GP 2 from BnNEt_2_, yield 120 mg (50%); from PhCH_2_CH_2_NEt_2_, yield 72 mg (30%); from NEt_3_, yield 203 mg (85%). Yellow solid. Mp 75–77 °C. ^1^H NMR (300 MHz, CDCl_3_) δ 8.64 (d, *J* = 2.8 Hz, 1H), 8.21 (dd, *J* = 9.6 Hz, 2.8 Hz 1H), 7.08 (d, *J* = 9.6 Hz, 1H), 3.38 (q, *J* = 7.2 Hz, 4H), 1.26 (t, *J* = 7.2 Hz, 6H). ^13^C NMR (75 MHz, CDCl_3_) δ 148.0, 137.2, 136.4, 127.5, 123.8, 118.4, 46.3, 12.3.

*N,N-Dibutyl-2,4-dinitroaniline* (**3b**) [38]. Yield 201 mg (68%). Orange liquid. ^1^H NMR (300 MHz, CDCl_3_) δ 8.65 (d, *J* = 2.8 Hz, 1H), 8.19 (dd, *J* = 9.5 Hz, 2.8 Hz 1H), 7.08 (d, *J* = 9.5 Hz, 1H), 3.28 (t, *J* = 7.3 Hz, 2H), 1.59 (m, 2H), 1.31 (m, 2H), 0.93 (t, *J* = 7.7 Hz, 3H). ^13^C NMR (75 MHz, CDCl_3_) δ 148.7, 137.7, 136.8, 127.6, 124.0, 118.8, 52.0, 29.4, 20.0, 13.7.

*1-(2,4-Dinitrophenyl)pyrrolidine* (**3c**) [39]. Yield 168 mg (71%). Yellow solid. Mp 97–98 °C. ^1^H NMR (300 MHz, CDCl_3_) δ 8.68 (d, *J* = 2.6 Hz, 1H), 8.21 (dd, *J* = 9.5 Hz, 2.6 Hz 1H), 6.92 (d, *J* = 9.5 Hz, 1H), 3.37 (m, 4H), 1.26 (m, 4H). ^13^C NMR (75 MHz, CDCl_3_) δ 145.6, 135.3, 134.8, 127.5, 123.8, 115.6, 51.1, 25.6.

*4-(2,4-Dinitrophenyl)morpholine* (**3d**) [40]. Yield 220 mg (87%). Yellow solid. Mp 116–118 °C. ^1^H NMR (300 MHz, CDCl_3_) δ 8.70 (d, *J* = 2.8 Hz, 1H), 8.29 (dd, *J* = 9.4 Hz, 2.8 Hz 1H), 7.13 (d, *J* = 9.4 Hz, 1H), 3.88 (m, 4H), 3.29 (m, 4H). ^13^C NMR (75 MHz, CDCl_3_) δ 149.3, 139.9, 138.6, 128.4, 123.6, 119.2, 66.2, 50.9.

*1-(2,4-Dinitrophenyl)-4-methylpiperidine* (**3e**) [41]. Yield 228 mg (86%). Yellow solid. Mp 84–86 °C. ^1^H NMR (300 MHz, CDCl_3_) δ 8.70 (d, *J* = 2.7 Hz, 1H), 8.23 (dd, *J* = 9.4 Hz, 2.7 Hz 1H), 7.09 (d, *J* = 9.4 Hz, 1H), 3.45 (m, 2H), 3.11 (m, 2H), 1.76–1.82 (m, 3H), 1.39 (m, 2H), 1.03 (d, *J* = 6.4 Hz, 3H). ^13^C NMR (75 MHz, CDCl_3_) δ 149.7, 137.3, 137.2, 128.0, 123.9, 119.2, 51.2, 33.6, 30.2, 21.6.

*Ethyl 1-(2,4-dinitrophenyl)piperidine-4-carboxylate* (**3f**). Yield 284 mg (88%). Yellow solid. Mp 94–96 °C (EtOH). ^1^H NMR (300 MHz, CDCl_3_) δ 8.72 (d, *J* = 2.6 Hz, 1H), 8.26 (dd, *J* = 9.3 Hz, 2.6 Hz 1H), 7.12 (d, *J* = 9.3 Hz, 1H), 4.21 (q, *J* = 7.1 Hz, 2H), 3.48 (m, 2H), 3.18 (m, 2H), 2.63 (m, 1H), 2.06–2.61 (m, 4H), 1.97 (t, *J* = 7.1 Hz, 3H). ^13^C NMR (75 MHz, CDCl_3_) δ 173.8, 149.6, 138.1, 137.9, 128.2, 123.8, 119.5, 60.8, 50.2, 39.9, 27.6, 14.2. HRMS (ESI): calcd for C_14_H_18_N_3_O_6_ [M + H], 324.1190; found, 324.1188.

*N,N-Dimethyl-2,4-dinitroaniline* (**3g**) [42]. Yield 179 mg (85%) from Me_2_NCH_2_CH_2_CN. Yield 68 mg (32%) from AllylNMe_2_. Yield 42 mg (20%) from 4-FPhCH_2_NMe_2_. Yield 2 mg (1%) from CyclohexylNMe_2_. Yield 19 mg (9%) from C_8_H_17_NMe_2_. Yield 40 mg (19%) from BnNMe_2_. Yield 21 mg (10%) from MeOCH_2_CH_2_NMe_2_. Yield 6 mg (3%) from *i*-PrNMe_2_. Yellow solid. Mp 84–86 °C. ^1^H NMR (300 MHz, CDCl_3_) δ 8.60 (d, *J* = 2.7 Hz, 1H), 8.15 (dd, *J* = 9.5 Hz, 2.7 Hz 1H), 7.01 (d, *J* = 9.5 Hz, 1H), 3.06 (s, 6H). ^13^C NMR (75 MHz, CDCl_3_) δ 149.2, 136.2, 135.5, 127.7, 124.1, 116.7, 42.4.

*N-Cyclohexyl-N-methyl-2,4-dinitroaniline* (**3h**) [43]. Yield 240 mg (86%). Yellow solid. Mp 115–118 °C. ^1^H NMR (300 MHz, CDCl_3_) δ 8.64 (d, *J* = 2.7 Hz, 1H), 8.16 (dd, *J* = 9.6 Hz, 2.7 Hz 1H), 7.08 (d, *J* = 9.6 Hz, 1H), 3.57 (m, 1H), 2.77 (s, 3H), 1.91 (m, 4H), 1.21–1.76 (m, 6H). ^13^C NMR (75 MHz, CDCl_3_) δ 149.4, 136.3, 136.1, 127.5, 124.3, 117.9, 62.1, 34.6, 29.8, 25.6, 25.4.

*N-Methyl-N-(1-methylethyl)-2,4-dinitroaniline* (**3i**). Yield 153 mg (64%). Yellow solid. Mp 73–75 °C (EtOH). ^1^H NMR (300 MHz, CDCl_3_) δ 8.66 (d, *J* = 2.7 Hz, 1H), 8.19 (dd, *J* = 9.6 Hz, 2.7 Hz 1H), 7.09 (d, *J* = 9.6 Hz, 1H), 4.03 (hept, *J* = 6.6 Hz, 1H), 2.75 (s, 3H), 1.33 (d, *J* = 6.6 Hz, 6H). ^13^C NMR (75 MHz, CDCl_3_) δ 149.3, 136.4, 136.3, 127.7, 124.3, 117.7, 53.3, 33.0, 19.4. HRMS (ESI): calcd for C_10_H_14_N_3_O_4_ [M + H], 240.0979; found, 240.0975.

*N-Methyl-2,4-dinitro-N-octylaniline* (**3j**). Yield 256 mg (83%). Orange liquid. ^1^H NMR (300 MHz, CDCl_3_) δ 8.65 (d, *J* = 2.7 Hz, 1H), 8.17 (dd, *J* = 9.5 Hz, 2.7 Hz 1H), 7.05 (d, *J* = 9.5 Hz, 1H), 3.38 (t, *J* = 7.6 Hz, 2H), 2.93 (s, 3H), 1.31 (m, 2H), 1.26–1.30 (m, 10H), 0.87 (t, *J* = 7.0 Hz, 3H). ^13^C NMR (75 MHz, CDCl_3_) δ 148.8, 136.2, 136.0, 127.6, 124.2, 117.2, 54.2, 40.5, 31.7, 29.2, 29.1, 26.9, 26.6, 22.6, 14.0. HRMS (ESI): calcd for C_15_H_24_N_3_O_4_ [M + H] 310.1761; found, 310.1758.

*N-(2-Methoxyethyl)-N-methyl-2,4-dinitroaniline* (**3k**). Yield 194 mg (76%). Orange liquid. ^1^H NMR (300 MHz, CDCl_3_) δ 8.62 (d, *J* = 2.6 Hz, 1H), 8.17 (dd, *J* = 9.5 Hz, 2.6 Hz 1H), 7.19 (d, *J* = 9.5 Hz, 1H), 3.65 (t, *J* = 5.1 Hz, 2H), 3.58 (t, *J* = 5.1 Hz, 2H), 3.35 (s, 3H), 3.00 (s, 3H). ^13^C NMR (75 MHz, CDCl_3_) δ 149.2, 136.7, 136.4, 127.5, 123.9, 118.3, 69.5, 59.1, 54.0, 40.3. HRMS (ESI): calcd for C_10_H_14_N_3_O_5_ [M + H], 256.0928, found, 256.0926.

*N-Benzyl-N-methyl-2,4-dinitroaniline* (**3l**) [44]. Yield 201 mg (70%). Yellow solid. Mp 143–145 °C. ^1^H NMR (300 MHz, CDCl_3_) δ 8.70 (d, *J* = 2.6 Hz, 1H), 8.16 (dd, *J* = 9.5 Hz, 2.6 Hz 1H), 7.22–7.39 (m, 5H), 7.05 (d, *J* = 9.5 Hz, 1H), 4.66 (s, 2H), 2.97 (s, 3H). ^13^C NMR (75 MHz, CDCl_3_) δ 149.1, 137.0, 136.4, 134.9, 129.2, 128.1, 127.7, 126.9, 124.0, 118.0, 57.8, 41.0.

*N-(4-Fluorobenzyl)-N-methyl-2,4-dinitroaniline* (**3m**). Yield 214 mg (70%). Orange liquid. ^1^H NMR (300 MHz, CDCl_3_) δ 8.68 (d, *J* = 2.7 Hz, 1H), 8.17 (dd, *J* = 9.5 Hz, 2.7 Hz 1H), 7.22 (m, 2H), 7.03–7.10 (m, 3H), 4.61 (s, 2H), 2.95 (s, 3H). ^13^C NMR (75 MHz, CDCl_3_) δ 162.4 (d, *J* = 245.5 Hz), 149.0, 137.3, 136.7, 130.7.8, 128.9 (d, *J* = 22.7 Hz), 127.8, 123.9, 118.1, 116.1 (d, *J* = 7.6 Hz), 57.2, 40.9. ^19^F NMR (282 MHz, CDCl_3_) δ −114.54. HRMS (ESI): calcd for C_14_H_12_FN_3_O_4_Na [M + Na], 328.0704; found, 328.0693.

*N-Methyl-2,4-dinitro-N-prop-2-en-1-ylaniline* (**3n**) [45]. Yield 130 mg (55%). Yellow solid. Mp 60–62 °C. ^1^H NMR (300 MHz, CDCl_3_) δ 8.65 (d, *J* = 2.7 Hz, 1H), 8.18 (dd, *J* = 9.5 Hz, 2.7 Hz 1H), 7.04 (d, *J* = 9.5 Hz, 1H), 5.88 (m, 1H), 5.35 (dd, *J* = 17.1 Hz, 10.2 Hz, 2H), 4.02 (d, *J* = 5.1 Hz, 2H), 2.94 (s, 3H). ^13^C NMR (75 MHz, CDCl_3_) δ 148.8, 136.7, 136.1, 130.7, 127.6, 124.0, 118.9, 117.7, 56.6, 40.1.

*N-Benzyl-N-ethyl-2,4-dinitroaniline* (**3o**) [46]. Yield 123 mg (41%). Yellow solid. Mp 72–73 °C. ^1^H NMR (300 MHz, CDCl_3_) δ 8.66 (d, *J* = 2.7 Hz, 1H), 8.18 (dd, *J* = 9.5 Hz, 2.7 Hz 1H), 7.24–7.36 (m, 5H), 7.09 (d, *J* = 9.5 Hz, 1H), 4.54 (s, 2H), 3.37 (q, *J* = 7.1 Hz, 2H), 1.26 (t, *J* = 7.1 Hz, 3H). ^13^C NMR (75 MHz, CDCl_3_) δ 148.7, 135.6, 129.0, 128.0, 127.6, 127.4, 123.7, 119.5, 55.2, 47.8, 12.4.

*N-(2,4-Dinitrophenyl)-N-ethyl-N-(2-phenylethyl)amine* (**3p**). Yield 178 mg (59%). Yellow solid. Mp 74–76 °C (EtOH). ^1^H NMR (300 MHz, CDCl_3_) δ 8.62 (d, *J* = 2.8 Hz, 1H), 8.17 (dd, *J* = 9.5 Hz, 2.8 Hz, 1H), 7.05–7.29 (m, 5H), 7.03 (d, *J* = 9.5 Hz, 1H), 3.54 (t, *J* = 7.4 Hz, 2H), 3.51 (q, *J* = 7.1 Hz, 2H), 3.41 (t, *J* = 7.4 Hz, 2H), 1.25 (t, *J* = 7.1 Hz, 3H). ^13^C NMR (75 MHz, CDCl_3_) δ 148.0, 137.9, 137.8, 137.0, 128.8, 128.7, 127.5, 126.9, 123.8, 118.8, 53.4, 47.2, 33.7, 12.5. HRMS (ESI): calcd for C_16_H_18_N_3_O_4_ [M + H], 316.1292; found, 316.1288.

*4-(Diethylamino)-2,3,5,6-tetrafluorobenzonitrile* (**4a**) [47]. Yield 112 mg (45%). Colorless solid. Mp 47–49 °C. ^1^H NMR (300 MHz, CDCl_3_) δ 3.41 (qm, *J* = 7.1 Hz, 4H), 1.20 (t, *J* = 7.1 Hz, 6H). ^13^C NMR (75 MHz, CDCl_3_) δ 148.2 (ddm, *J* = 256 Hz, 19 Hz), 140.6 (dm, *J* = 247 Hz), 134.3 (m), 108.7 (t, *J* = 4 Hz), 82.5 (t, *J* = 18 Hz), 46.8 (t, *J* = 5 Hz), 13.6 (t, *J* = 2 Hz). ^19^F NMR (282 MHz, CDCl3) δ −136.5 (q, *J* = 14 Hz), −151.1 (q, *J* = 14 Hz).

*N,N-Diethyl-2,3,5,6-tetrafluoropyridin-4-amine* (**4b**) [47]. Yield 164 mg (74%). Colorless oil. ^1^H NMR (300 MHz, CDCl_3_) δ 3.44 (q, *J* = 7.1 Hz, 4H), 1.24 (tm, *J* = 7.1 Hz, 6H). ^13^C NMR (75 MHz, CDCl_3_) δ 145.2 (ddd, *J* = 236 Hz, 32 Hz, 2 Hz), 139.4 (m), 134.3 (dm, *J* = 249 Hz), 46.6 (t, *J* = 5 Hz), 13.8. ^19^F NMR (282 MHz, CDCl_3_) δ −96.7 (m), −158.2 (m).

*4-(Cyclohexylsulfanyl)-N,N-diethyl-3,5,6-trifluoropyridin-2-amine* (**4c**). Yield 162 mg (51%). Colorless oil. ^1^H NMR (300 MHz, CDCl_3_) δ 3.44 (dq, *J* = 7.0 Hz, 1.7 Hz, 4H), 1.63–1.95 (m, 4H), 1.30–1.59 (m, 7H), 1.17 (t, *J* = 7.0 Hz, 6H). ^13^C NMR (75 MHz, CDCl_3_) δ 144.5 (ddd, *J* = 247 Hz, 16 Hz, 4 Hz), 143.6 (dm, *J* = 239 Hz), 141.3 (m), 135.2 (dd, *J* = 240 Hz, 21 Hz), 124.9 (ddd, *J* = 21 Hz, 18 Hz, 4 Hz), 46.0 (d, *J* = 3 Hz), 44.2 (d, *J* = 6 Hz), 33.5, 25.8, 25.5, 13.6 (d, *J* = 1 Hz). ^19^F NMR (282 MHz, CDCl_3_) δ −93.3 (m), −130.7 (m), −152.6 (m). HRMS (ESI): calcd for C_15_H_23_F_3_N_2_S [M + H], 319.1450; found, 319.1462.

*N,N-Diethyl-3,5,6-trifluoro-4-(phenylsulfanyl)pyridin-2-amine* (**4d**). Yield 203 mg (65%). Colorless oil. ^1^H NMR (300 MHz, CDCl_3_) δ 7.26–7.29 (m, 2H), 7.12–7.17 (m, 3H), 3.30 (dq, *J* = 7.1 Hz, 1.7 Hz, 4H), 1.04 (t, *J* = 7.1 Hz, 6H). ^13^C NMR (75 MHz, CDCl_3_) δ 144.8 (ddd, *J* = 232 Hz, 16 Hz, 3 Hz), 142.2 (dm, *J* = 241 Hz), 141.3 (m), 134.6 (ddd, *J* = 248 Hz, 30 Hz, 2 Hz), 133.0, 132.4, 130.8, 127.8, 125.0 (dm, *J* = 22 Hz), 44.2 (d, *J* = 6 Hz), 13.6 (d, *J* = 1 Hz). ^19^F NMR (282 MHz, CDCl3) δ −92.4 (m), -130.6 (m), −152.7 (m). HRMS (ESI): calcd for C_15_H_16_F_3_N_2_S [M + H], 313.0971; found, 313.0972.

*4-(1,3-Benzothiazol-2-ylsulfanyl)-N,N-diethyl-3,5,6-trifluoropyridin-2-amine* (**4e**). Yield 207 mg (56%). Colorless solid. Mp 87–88 °C (CCl_4_). ^1^H NMR (300 MHz, CDCl_3_) δ 7.89 (d, *J* = 8.1 Hz, 1H), 7.73 (d, *J* = 7.9 Hz, 1H), 7.41 (m, 1H), 7.33 (m, 1H), 3.47 (dq, *J* = 7.1 Hz, 1.8 Hz, 4H), 1.21 (t, *J* = 7.1 Hz, 6H). ^13^C NMR (75 MHz, CDCl_3_) δ 161.7, 153.2, 144.9 (ddd, *J* = 232 Hz, 16 Hz, 3 Hz), 142.6 (dm *J* = 259 Hz), 141.1 (m), 135.9, 134.2 (ddd, *J* = 253 Hz, 32 Hz, 2 Hz), 126.5, 125.2, 122.4, 121.0, 120.2 (dm, *J* = 26 Hz), 44.3 (d, *J* = 6 Hz), 13.6 (d, *J* = 2 Hz). ^19^F NMR (282 MHz, CDCl_3_) δ −90.0 (m), −126.9 (m), −148.9 (m). HRMS (ESI): calcd for C_16_H_15_F_3_N_3_S_2_ [M + H], 370.0654; found, 370.0649.

*N,N-Diethyl-1,3-benzoxazol-2-amine* (**4f**) [48]. Yield 179 mg (87%). Colorless oil. ^1^H NMR (300 MHz, CDCl_3_) δ 7.39 (d, *J* = 7.8 Hz, 1H), 7.27 (d, *J* = 8.1 Hz, 1H), 7.15 (m, 1H), 7.03 (m, 1H), 3.62 (q, *J* = 7.0 Hz, 4H), 1.31 (t, *J* = 7.0 Hz, 6H). ^13^C NMR (75 MHz, CDCl_3_) δ 162.1, 148.8, 143.5, 123.8, 120.0, 115.7, 108.5, 42.9, 13.4.

*N,N-Diethyl-5-methyl-1,3-benzoxazol-2-amine* (**4g**) [49]. Yield 171 mg (84%). Colorless oil. ^1^H NMR (300 MHz, CDCl_3_) δ 7.19 (d, *J* = 0.6 Hz, 1H), 7.13 (d, *J* = 8.1 Hz, 1H), 6.81 (dd, *J* = 8.1 Hz, 0.6 Hz, 1H), 3.61 (q, *J* = 7.1 Hz, 4H), 2.40 (s, 3H), 1.29 (t, *J* = 7.1 Hz, 6H). ^13^C NMR (75 MHz, CDCl_3_) δ 162.3, 146.9, 143.6, 133.4, 120.6, 116.2, 107.8, 42.9, 21.5, 13.6.

*N,N-Diethyl-5-phenyl-1,3,4-thiadiazol-2-amine* (**4h**) [50]. Yield 179 mg (77%). Colorless solid. Mp 40–42 °C. ^1^H NMR (300 MHz, CDCl_3_) δ 7.80–7.83 (m, 2H), 7.41–7.44 (m, 3H), 3.62 (q, *J* = 7.1 Hz, 4H), 1.33 (t, *J* = 7.0 Hz, 6H). ^13^C NMR (75 MHz, CDCl_3_) δ 170.6, 153.2, 131.7, 129.4, 129.3, 127.6, 46.2, 11.3.

*2-Chloro-N,N-diethylpyrimidin-4-amine* (**4i**) [51]. Yield 133 mg (72%). Colorless oil. ^1^H NMR (300 MHz, CDCl_3_) δ 7.90 (d, *J* = 6.2 Hz, 1H), 6.19 (d, *J* = 6.2 Hz, 1H), 3.43 (m, 4H), 1.13 (t, *J* = 7.1 Hz, 6H). ^13^C NMR (75 MHz, CDCl_3_) δ 161.8, 160.7, 156.5, 101.0, 42.5, 12.5.

*3-Chloro-N,N-diethylpyrazin-2-amine* (**4j**). Yield 154 mg (83%). Colorless oil. ^1^H NMR (300 MHz, CDCl_3_) δ 8.03 (d, *J* = 2.5 Hz, 1H), 7.73 (d, *J* = 2.5 Hz, 1H), 3.53 (q, *J* = 7.1 Hz, 4H), 1.22 (t, *J* = 7.1 Hz, 6H). ^13^C NMR (75 MHz, CDCl_3_) δ 162.4, 153.8, 139.4, 132.8, 44.3, 13.2. HRMS (ESI): calcd for C_8_H_13_ClN_3_ [M + H], 186.0793; found, 186.0783.

*N,N-Diethyl-3-phenyl-1,2,4-oxadiazol-5-amine* (**4k**) [52]. Yield 193 mg (89%). Colorless oil. ^1^H NMR (300 MHz, CDCl_3_) δ 8.01–8.04 (m, 2H), 7.44–7.47 (m, 3H), 3.58 (q, *J* = 7.1 Hz, 4H), 1.31 (t, *J* = 7.0 Hz, 6H). ^13^C NMR (75 MHz, CDCl_3_) δ 171.0, 168.7, 130.5, 128.5, 128.0, 127.2, 43.5, 13.3.

*N,N-Diethyl-2,6-dinitro-4-(trifluoromethyl)aniline* (**4l**) [53]. Yield 236 mg (77%). Yellow solid. Mp 95–96 °C. ^1^H NMR (300 MHz, CDCl_3_) δ 8.09 (s, 2H), 3.13 (q, *J* = 7.0 Hz, 4H), 1.19 (t, *J* = 7.0 Hz, 6H). ^13^C NMR (75 MHz, CDCl_3_) δ 145.9, 141.2, 126.5, 123.5 (q, *J* = 271 Hz), 122.2, 46.2, 12.7. ^19^F NMR (282 MHz, CDCl_3_) δ −63.8.

*N,N-Diethyl-5-nitropyridin-2-amine* (**4m**) [54]. Yield 193 mg (99%). Yellow solid. Mp 72–75 °C. ^1^H NMR (300 MHz, CDCl_3_) δ 9.00 (d, 1.5 Hz, 1H), 8.12 (dd, *J* = 9.5 Hz, 1.5 Hz, 1H), 6.41 (d, *J* = 9.5 Hz, 1H), 3.60 (q, *J* = 7.1 Hz, 4H), 1.21 (t, *J* = 7.2 Hz, 6H). ^13^C NMR (75 MHz, CDCl_3_) δ 159.5, 146.9, 134.2, 132.6, 104.1, 43.5, 12.7.

*N,N-Diethyl-3-nitropyridin-2-amine* (**4n**) [55]. Yield 189 mg (97%). Orange oil. ^1^H NMR (300 MHz, CDCl_3_) δ 8.28 (dd, *J* = 4.5 Hz, 1.7 Hz, 1H), 8.01 (dd, *J* = 8.0 Hz, 1.7 Hz, 1H), 6.63 (dd, *J* = 8.1 Hz, 4.5 Hz, 1H), 3.42 (q, *J* = 7.2 Hz, 4H), 1.20 (t, *J* = 7.2 Hz, 6H). ^13^C NMR (75 MHz, CDCl_3_) δ 152.1, 151.2, 135.2, 132.7, 117.8, 44.1, 12.6.

*5-Bromo-N,N-diethyl-3-nitropyridin-2-amine* (**4o**). Yield 262 mg (96%). Orange oil. ^1^H NMR (300 MHz, CDCl_3_) δ 8.31 (d, *J* = 2.2 Hz, 1H), 8.15 (d, *J* = 2.2 Hz, 1H), 3.42 (q, *J* = 7.1 Hz, 4H), 1.20 (t, *J* = 7.1 Hz, 6H). ^13^C NMR (75 MHz, CDCl_3_) δ 151.9, 150.6, 136.8, 104.3, 44.3, 12.5. HRMS (ESI): calcd for C_9_H_13_BrN_3_O_2_ [M + H], 274.0186; found, 274.0194.

*N,N-Diethyl-3-(1-methylethyl)-1,2,4-oxadiazol-5-amine* (**4p**). Yield 118 mg (86%). Colorless oil. ^1^H NMR (300 MHz, CDCl_3_) δ 3.43 (q, *J* = 7.1 Hz, 4H), 2.82 (h, *J* = 7.0 Hz, 1H), 1.23 (d, *J* = 7.0 Hz, 6H), 1.19 (t, *J* = 7.1 Hz, 6H). ^13^C NMR (75 MHz, CDCl_3_) δ 175.6, 170.6, 43.3, 27.0, 20.4, 13.2. HRMS (ESI): calcd for C_9_H_18_N_2_O [M + H], 184.1444; found, 184.1451.

#### 3.2.3. Mechanistic Experiment

*fac*-Ir(ppy)_3_ (2 mg, 0.003 mmol) and *N*-methylpyrrolidine (170 mg, 2 mmol) were added to a solution of pentafluoropyridine (169 mg, 1 mmol) in acetonitrile (2 mL). The mixture was irradiated at room temperature with blue LED for 20 h. An aliquot was withdrawn and analyzed by ^1^H, ^13^C, ^19^F NMR. The solvent was evaporated under vacuum, the residue was washed with methyl *tert*-butyl ether (4 × 5 mL). The obtained solid was dried under vacuum and dissolved in DMSO-d_6_. NMR analysis indicated the presence of *N,N*-dimethylpyrrolidinium salt. ^1^H NMR (300 MHz, DMSO-d_6_) δ 3.47 (m, 4H), 3.11 (s, 6H), 2.10 (m, 4H). ^13^C NMR (75 MHz, DMSO-d_6_) δ 65.3, 51.6, 21.8. ^19^F NMR (282 MHz, DMSO-d_6_) δ −138.4 (br).

## 4. Conclusions

A method for the dealkylative arylation of tertiary amines by means of electron deficient aromatic and heteroaromatic halides under photoredox conditions is described. The reaction proceeds through the generation of aryl radicals, which interact with the amines followed by the dealkylation of the tryalkylamino fragment.

## Figures and Tables

**Table 1:**
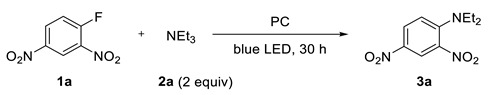
Optimization studies.

Entry	PC ^1^	Solv.	Ratio 1a:3a
1 ^2^	–	DMSO	100:0
2 ^2,3^	–	DMSO	95:5
3	–	DMSO	90:10
4	Ru(phen)_3_(PF_6_)_2_ (1%)	DMSO	68:32
5	4CzIPN (2%)	DMSO	33:67
6	3DPA2FBN (2%)	DMSO	28:72
7	Ir(ppy)_2_dtbbpy PF_6_ (0.5%)	DMSO	29:71
8	Ir(ppy)_3_ (0.3%)	DMSO	15:85 (84) ^4^
9	Ir(ppy)_3_ (0.3%), 1.1 equiv of NEt_3_	DMSO	61:39
10	Ir(ppy)_3_ (0.3%)	DMF	35:65
11	Ir(ppy)_3_ (0.3%)	MeCN	57:43
12	Ir(ppy)_3_ (0.3%)	CH_2_Cl_2_	85:15

^1^ Abbreviations: 4CzIPN, 1,2,3,5-tetrakis(carbazol-9-yl)-4,6-dicyanobenzene; 3DPA2FBN, 2,4,6-tris(diphenylamino)-3,5-difluorobenzonitrile. ^2^ In the dark. ^3^ At 50 °C for 60 h. ^4^ Isolated yield.

**Table 2:**
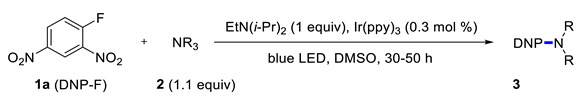
Reaction of fluoride **1a** with amines.

Amine	Product		Y., % ^1^	Amine	Major Product		Y., % ^1^	Minor Product		Y., % ^1^
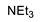	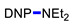	**3a**	84	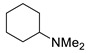	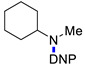	**3h**	86	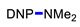	**3g**	1
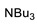	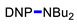	**3b**	68	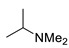	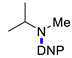	**3i**	64	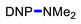	**3g**	3
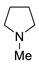	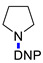	**3c**	71	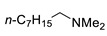	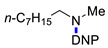	**3j**	83	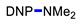	**3g**	9
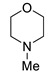	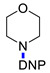	**3d**	87	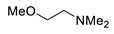	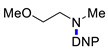	**3k**	76	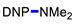	**3g**	10
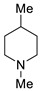	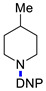	**3e**	86	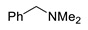	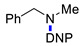	**3l**	70	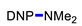	**3g**	19
				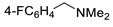	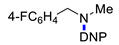	**3m**	70	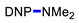	**3g**	20
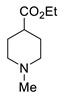	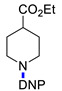	**3f**	88	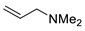	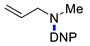	**3n**	55	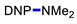	**3g**	32
				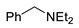	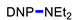	**3a**	50	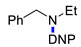	**3o**	41
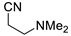	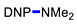	**3g**	85	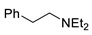	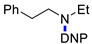	**3p**	59	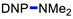	**3g**	30

^1^ Isolated yield.

## Data Availability

The data presented in this study are available in this article.

## Supplementary Materials

The following are available online, Figures S1–S4: Cyclic voltammetry curves; Cartesian coordinates and energies; Copies of NMR spectra.
