# Supplementary material for: Photoredox Catalyzed Dealkylative Aromatic Halogen Substitution with Tertiary Amines
Source: Molecules. 2021 Jun 1;26(11):3323. doi: 10.3390/molecules26113323 (PMC8199326; doi:10.3390/molecules26113323)
Supplement: Supplementary file 1 [file molecules-26-03323-s001.zip › molecules-1223021-supplementary.pdf]

Supporting Information

Photoredox Catalyzed Dealkylative Aromatic Halogen Substitution with Tertiary Amines

Dmitry L. Lipilin [1], Alexander E. Frumkin [1], Alexey Yu. Tyurin [1], Vitalij V. Levin [1], Alexander D. Dilman [1],*

[1] N. D. Zelinsky Institute of Organic Chemistry, 119991 Moscow, Leninsky prosp. 47, Russian Federation

Content

	Page
Cyclic voltammetry	S2
Quantum chemical calculations	S3-S9
NMR spectra	S10-S83

Cyclic voltammetry

Voltammetric studies were carried out using potentiostat P30JM with a scan rate of 0.1 V·s^{-1} in a temperature-controlled (25 °C) glass cell (V = 10 mL) under an argon atmosphere. A glassy carbon disk (*d* = 2.5 mm) was used as the working electrode (carefully polished before each measurement). A saturated calomel electrode (SCE) separated from the solution being studied by a salt bridge filled with the supporting electrolyte (0.1 M Et_4NClO_4 in DMSO) was used as the reference electrode. A platinum plate (*S* = 3 cm^2) was used as the counter electrode. All experiments were performed with the concentration of studied compounds of 1 mM.

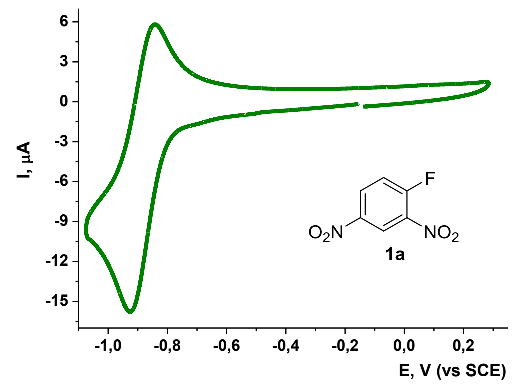

Figure S1. Compound **1a** (initial cathodic scan). E_{onset}^{red} = -0.76 V, E_{p1}^{red} = -0.93 V, i_{p1}^{red} = 13.66 µA; E_{p1}^{reox} = -0.84 V

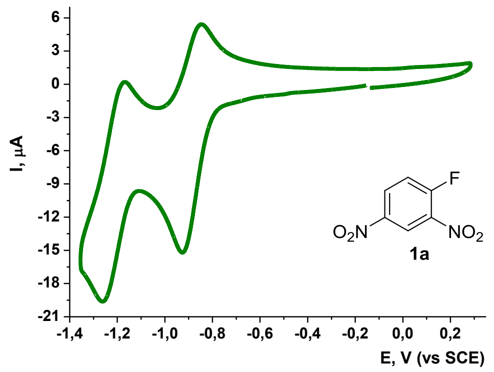

Figure S2. Compound **1a** (initial cathodic scan). E_{onset}^{red} = -0.76 V, E_{p1}^{red} = -0.93 V, i_{p1}^{red} = 13.66 µA; E_{p2}^{red} = -1.26 V, E_{p2}^{reox} = -1.17 V, E_{p1}^{reox} = -0.84 V

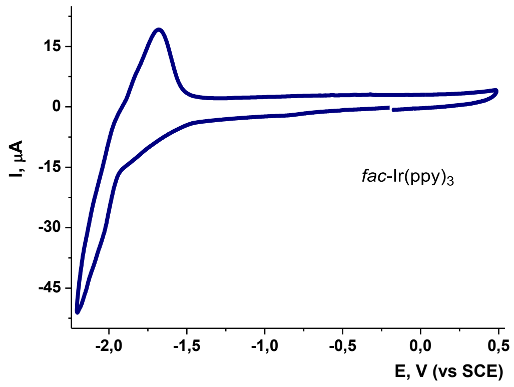

Figure S3. Compound *fac*-Ir(ppy)$_3$ (initial cathodic scan). E_p^{red} > 2.19 V (supporting electrolyte discharge)

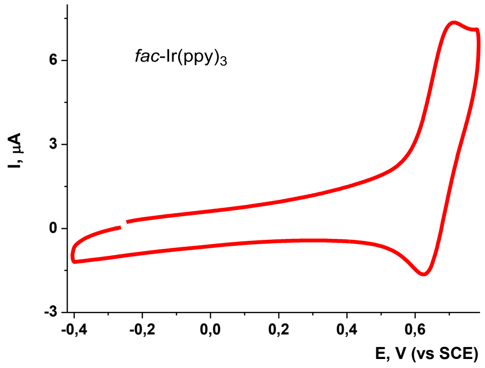

Figure S4. Compound *fac*-Ir(ppy)$_3$ (initial anodic scan). E_{onset}^{ox} = +0.52 V, E_p^{ox} = +0.70 V, i_p^{ox} = 5.26 µA; E_p^{rered} = +0.63 V

For *fac*-Ir(ppy)$_3$, CV measurements provide potentials of ground states. For comparison, literature values of ground states, as well as of the excited state, are given below (vs SCE):[1]

Ir(IV)/Ir(III)	+0.77 V	Ir(IV)/Ir(III)*	-1.73 V
Ir(III)/Ir(II)	-2.19 V	Ir(III)*/Ir(II)	+0.31 V

[1] Prier, C. K.; Rankic, D. A.; MacMillan, D. W. C. Visible light photoredox catalysis with transition metal complexes: applications in organic synthesis. *Chem. Rev.* **2013**, *113*, 5322–5363.

Quantum chemical calculations

Calculations were performed using Gaussian program[2] at the M06-2X/6-31+G(d) level. Stationary points were verified by frequency calculations. Then, for optimized structures, single point energies were calculated by CPCM method, DMSO as solvent. The free energy in solution was obtained as a sum of electronic energy in solution and thermal correction to Gibbs free energy.

NMe_3

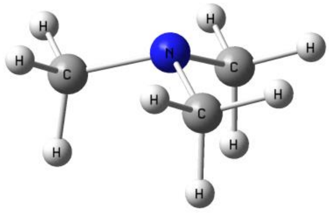

C	0.00000000	1.37944600	-0.06249600
H	0.88705100	1.89328700	0.32036100
H	0.00000000	1.46213000	-1.16737300
H	-0.88705100	1.89328700	0.32036100
C	1.19463500	-0.68972300	-0.06249600
H	2.08316000	-0.17843400	0.32036100
H	1.19610800	-1.71485200	0.32036100
H	1.26624200	-0.73106500	-1.16737300
C	-1.19463500	-0.68972300	-0.06249600
H	-1.19610800	-1.71485200	0.32036100
H	-2.08316000	-0.17843400	0.32036100
H	-1.26624200	-0.73106500	-1.16737300
N	0.00000000	0.00000000	0.38641200

E(RM062X) = -174.380781123

Zero-point correction= 0.121831 (Hartree/Particle)
Thermal correction to Energy= 0.127279
Thermal correction to Enthalpy= 0.128223
Thermal correction to Gibbs Free Energy= 0.095521
Sum of electronic and zero-point Energies= -174.258950
Sum of electronic and thermal Energies= -174.253502
Sum of electronic and thermal Enthalpies= -174.252558
Sum of electronic and thermal Free Energies= -174.285260

SCRF CPCM (in DMSO): SCF Done: E(RM062X) = -174.383601984

[2] Gaussian 09, Revision D.01, M. J. Frisch, G. W. Trucks, H. B. Schlegel, G. E. Scuseria, M. A. Robb, J. R. Cheeseman, G. Scalmani, V. Barone, B. Mennucci, G. A. Petersson, H. Nakatsuji, M. Caricato, X. Li, H. P. Hratchian, A. F. Izmaylov, J. Bloino, G. Zheng, J. L. Sonnenberg, M. Hada, M. Ehara, K. Toyota, R. Fukuda, J. Hasegawa, M. Ishida, T. Nakajima, Y. Honda, O. Kitao, H. Nakai, T. Vreven, J. A. Montgomery, Jr., J. E. Peralta, F. Ogliaro, M. Bearpark, J. J. Heyd, E. Brothers, K. N. Kudin, V. N. Staroverov, T. Keith, R. Kobayashi, J. Normand, K. Raghavachari, A. Rendell, J. C. Burant, S. S. Iyengar, J. Tomasi, M. Cossi, N. Rega, J. M. Millam, M. Klene, J. E. Knox, J. B. Cross, V. Bakken, C. Adamo, J. Jaramillo, R. Gomperts, R. E. Stratmann, O. Yazyev, A. J. Austin, R. Cammi, C. Pomelli, J. W. Ochterski, R. L. Martin, K. Morokuma, V. G. Zakrzewski, G. A. Voth, P. Salvador, J. J. Dannenberg, S. Dapprich, A. D. Daniels, O. Farkas, J. B. Foresman, J. V. Ortiz, J. Cioslowski, and D. J. Fox, Gaussian, Inc., Wallingford CT, 2013.

2,3,5,6-Tetrafluoropyridin-4-yl radical

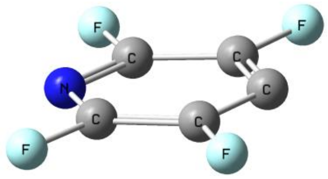

C	0.00000000	1.20477000	-0.73434800
C	0.00000000	1.12917500	0.65952200
C	0.00000000	-1.12917500	0.65952200
C	0.00000000	-1.20477000	-0.73434800
C	0.00000000	0.00000000	-1.39115100
F	0.00000000	2.37829900	-1.35882500
F	0.00000000	-2.37829900	-1.35882500
F	0.00000000	-2.25822400	1.35663400
F	0.00000000	2.25822400	1.35663400
N	0.00000000	0.00000000	1.32632100

SCF Done: E(UM062X) = -644.328662541

Zero-point correction= 0.045011 (Hartree/Particle)
Thermal correction to Energy= 0.052342
Thermal correction to Enthalpy= 0.053286
Thermal correction to Gibbs Free Energy= 0.012542
Sum of electronic and zero-point Energies= -644.283652
Sum of electronic and thermal Energies= -644.276321
Sum of electronic and thermal Enthalpies= -644.275377
Sum of electronic and thermal Free Energies= -644.316120

SCRF CPCM (in DMSO): SCF Done: E(UM062X) = -644.333355151

Complex A1

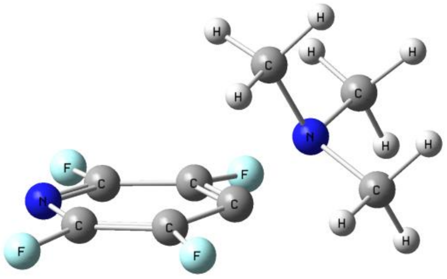

C	-0.27696800	-1.16348600	-0.33855400
C	-1.59147600	-1.19102000	0.05071400
C	-1.72807600	1.03297700	0.08513800
C	-0.41490100	1.19078100	-0.30009900
C	0.42625300	0.06182700	-0.51784200
F	0.35560200	-2.34538500	-0.59833100
F	0.00242200	2.44165900	-0.64568200
F	-2.48401700	2.13089100	0.20881900
F	-2.20266900	-2.37795000	0.15519000
N	-2.33085100	-0.12153400	0.30968600
C	1.79521500	-0.55710600	1.52220900
H	1.51035900	-1.60346200	1.40939200

H	2.77220300	-0.47930000	2.00661500
H	1.03649600	-0.02844200	2.10286000
C	2.81362800	-0.67903600	-0.69974100
H	2.44827400	-1.69599000	-0.82678400
H	2.84754800	-0.17901800	-1.66812900
H	3.80045000	-0.67860800	-0.22843400
C	2.36735700	1.46733900	0.31620200
H	2.36654500	1.94444000	-0.66367500
H	1.70980600	2.01711800	0.98953900
H	3.37963200	1.42423400	0.72477400
N	1.86520100	0.07331100	0.17113200

SCF Done: E(UM062X) = -818.732018438

Zero-point correction=   0.171357 (Hartree/Particle)
Thermal correction to Energy=   0.184317
Thermal correction to Enthalpy=   0.185261
Thermal correction to Gibbs Free Energy=   0.131897
Sum of electronic and zero-point Energies=   -818.560661
Sum of electronic and thermal Energies=   -818.547702
Sum of electronic and thermal Enthalpies=   -818.546758
Sum of electronic and thermal Free Energies=   -818.600121
SCRF CPCM (in DMSO): SCF Done: E(UM062X) = -818.751638317

Benzoxazol-2-yl radical

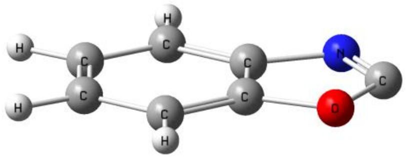

C	-0.60223500	-0.52151100	0.00000000
C	0.00000000	0.73855400	0.00000000
C	1.36579000	0.94737400	0.00000000
C	2.14399400	-0.21048700	0.00000000
C	1.56476400	-1.48907000	0.00000000
C	0.18358100	-1.66914900	0.00000000
C	-2.11887500	0.90698700	0.00000000
H	1.79919600	1.94123200	0.00000000
H	3.22538100	-0.11754100	0.00000000
H	2.21238200	-2.36007800	0.00000000
H	-0.26794300	-2.65550300	0.00000000
N	-2.00491500	-0.35089100	0.00000000
O	-1.01959100	1.67899300	0.00000000

SCF Done: E(UM062X) = -398.880526387

Zero-point correction=   0.094239 (Hartree/Particle)
Thermal correction to Energy=   0.099960
Thermal correction to Enthalpy=   0.100904
Thermal correction to Gibbs Free Energy=   0.063616
Sum of electronic and zero-point Energies=   -398.786287
Sum of electronic and thermal Energies=   -398.780566
Sum of electronic and thermal Enthalpies=   -398.779622
Sum of electronic and thermal Free Energies=   -398.816910

SCRF CPCM (in DMSO): SCF Done: E(UM062X) = -398.885517705

Complex A2

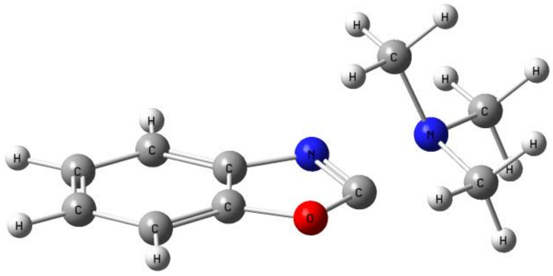

C	-1.09873300	0.74721001	-0.22220796
C	-1.11036999	-0.66241699	-0.21216306
C	-2.24062399	-1.42438102	-0.00330113
C	-3.43483400	-0.72187004	0.21818190
C	-3.45529901	0.67752396	0.21702100
C	-2.30009401	1.43095698	-0.00168293
C	0.89386601	0.09352506	-0.72470998
H	-2.20398098	-2.50912702	-0.01062420
H	-4.35452599	-1.27322006	0.38783385
H	-4.39788201	1.19085494	0.38613703
H	-2.32216002	2.51624298	-0.00879285
N	0.17671500	1.20348803	-0.45093191
O	0.16714101	-1.09590797	-0.45540207
C	1.91393098	-0.02295409	1.56213202
H	1.29523698	-0.89786011	1.76684295
H	2.84901697	-0.07264613	2.12623603
H	1.36030297	0.88674489	1.79884608
C	3.01609499	1.20641504	-0.23453888
H	3.95448599	1.17101301	0.32439813
H	3.20074601	1.20025112	-1.30973287
H	2.42667898	2.08452402	0.02693018
C	2.94676801	-1.22679095	-0.29823005
H	3.09647503	-1.19305088	-1.37845504
H	3.90417101	-1.25731598	0.22793996
H	2.33811802	-2.09301698	-0.04065012
N	2.21356400	0.00087801	0.09997503

SCF Done: E(UM062X) = -573.286916961

Zero-point correction= 0.220546 (Hartree/Particle)
Thermal correction to Energy= 0.231833
Thermal correction to Enthalpy= 0.232777
Thermal correction to Gibbs Free Energy= 0.183371
Sum of electronic and zero-point Energies= -573.066371
Sum of electronic and thermal Energies= -573.055084
Sum of electronic and thermal Enthalpies= -573.054140
Sum of electronic and thermal Free Energies= -573.103546

SCRF CPCM (in DMSO): SCF Done: E(UM062X) = -573.304933469

2,4-Dinitrophenyl radical

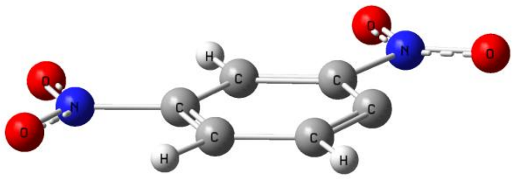

C	0.10725800	-2.26245300	0.00000000
C	-1.12525500	-1.60281600	0.00000000
C	0.00000000	0.57411100	0.00000000
C	1.21295200	-0.10240900	0.00000000
C	1.23595100	-1.47706200	0.00000000
C	-1.14652400	-0.21018300	0.00000000
N	2.47886400	0.64710200	0.00000000
O	3.50118600	-0.01040900	0.00000000
O	2.40866200	1.86067300	0.00000000
N	-2.45572300	0.47293600	0.00000000
O	-2.45007500	1.68842700	0.00000000
O	-3.44996600	-0.22832400	0.00000000
H	0.16114900	-3.34604500	0.00000000
H	-2.06148600	-2.15014200	0.00000000
H	-0.04638500	1.65785100	0.00000000

SCF Done: E(UM062X) = -640.319005647

Zero-point correction= 0.094420 (Hartree/Particle)
Thermal correction to Energy= 0.103620
Thermal correction to Enthalpy= 0.104565
Thermal correction to Gibbs Free Energy= 0.058076
Sum of electronic and zero-point Energies= -640.224585
Sum of electronic and thermal Energies= -640.215385
Sum of electronic and thermal Enthalpies= -640.214441
Sum of electronic and thermal Free Energies= -640.260930

SCRF CPCM (in DMSO): SCF Done: E(UM062X) = -640.331014352

Complex A3

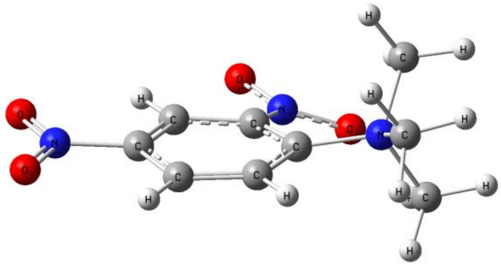

C	-0.04610500	-1.67440500	-0.27819200
C	-1.42862500	-1.60023100	-0.27673700
C	-1.24853000	0.79727200	0.09642500
C	0.16432600	0.74791800	-0.01885500
C	0.77553100	-0.55592100	-0.10655300
C	2.52792900	-2.22360000	0.49676000
H	2.34006800	-2.88443400	-0.34712800
H	3.58347600	-2.28387300	0.76197500

H	1.90826200	-2.49815800	1.35029100
C	3.05505700	-0.53149900	-1.13732200
H	2.69263000	-1.21656400	-1.90508200
H	2.89872300	0.50505300	-1.42431600
H	4.10438300	-0.73326800	-0.90852800
C	2.74796900	0.03015800	1.27534700
H	2.68772600	1.08322000	1.01205600
H	2.12717100	-0.21215300	2.13919700
H	3.78584800	-0.25434700	1.45815200
N	2.24652700	-0.79854800	0.11288300
C	-1.99715100	-0.33934400	-0.03713400
N	0.79461300	1.98510400	-0.13743000
O	1.99639700	2.03462600	-0.55714100
O	0.16156800	3.02048600	0.16399000
N	-3.46118600	-0.23096100	0.04986400
O	-3.94493800	0.85902600	0.29329200
O	-4.10171700	-1.25487100	-0.12736000
H	0.38298500	-2.65970600	-0.40098500
H	-2.04875900	-2.47514300	-0.41666600
H	-1.71508100	1.76398700	0.23913100

SCF Done: E(UM062X) = -814.736417033

Zero-point correction= 0.222686 (Hartree/Particle)
Thermal correction to Energy= 0.236946
Thermal correction to Enthalpy= 0.237891
Thermal correction to Gibbs Free Energy= 0.180847
Sum of electronic and zero-point Energies= -814.513731
Sum of electronic and thermal Energies= -814.499471
Sum of electronic and thermal Enthalpies= -814.498526
Sum of electronic and thermal Free Energies= -814.555570

SCRF CPCM (in DMSO): SCF Done: E(UM062X) = -814.771594087

3-Nitropyridine-6-yl radical

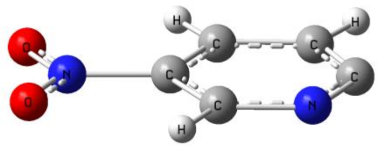

C	1.18006500	-1.90146800	0.00000000
C	1.21638200	-0.51228000	0.00000000
C	-1.21240500	-0.50672000	0.00000000
C	-0.09173600	-2.46566200	0.00000000
C	0.00000000	0.17027800	0.00000000
N	-0.00202600	1.63995500	0.00000000
O	-1.08384100	2.19756300	0.00000000
O	1.07864200	2.20057900	0.00000000
H	2.08268000	-2.50036200	0.00000000
H	2.14814900	0.04377100	0.00000000
H	-2.15879900	0.02282900	0.00000000
N	-1.22429800	-1.84799100	0.00000000

SCF Done: E(UM062X) = -451.932264419

Zero-point correction= 0.079891 (Hartree/Particle)
Thermal correction to Energy= 0.086511

Thermal correction to Enthalpy= 0.087455
Thermal correction to Gibbs Free Energy= 0.047484
Sum of electronic and zero-point Energies= -451.852374
Sum of electronic and thermal Energies= -451.845753
Sum of electronic and thermal Enthalpies= -451.844809
Sum of electronic and thermal Free Energies= -451.884780

SCRF CPCM (in DMSO): SCF Done: E(UM062X) = -451.940630581

Complex A4

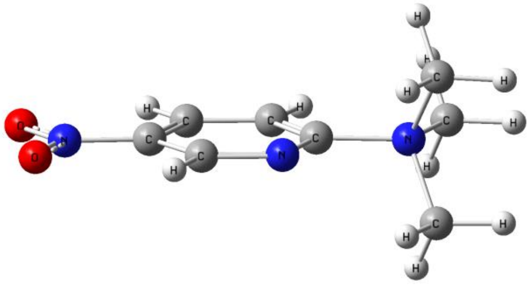

C	0.26613500	1.33001400	0.00001300
C	-1.11383900	1.30011800	0.00005300
C	-0.97050800	-1.13828900	0.00014700
C	0.94274200	0.10338600	-0.00011100
C	3.11678000	1.30340600	-0.00060900
H	2.82550000	1.85408000	0.89415000
H	4.19478100	1.13441200	-0.00094400
H	2.82481100	1.85362300	-0.89543700
C	2.86656000	-0.78303400	1.22112900
H	2.57017400	-0.20720400	2.09863400
H	2.34535900	-1.73922900	1.21137600
H	3.95022100	-0.91961400	1.19171400
C	2.86663700	-0.78410400	-1.22049500
H	2.34552400	-1.74034800	-1.20994100
H	2.57027500	-0.20912600	-2.09857100
H	3.95030800	-0.92053200	-1.19083300
N	2.43793100	-0.02051300	-0.00005100
C	-1.76693900	0.03788300	0.00003900
N	-3.14900200	-0.05448900	-0.00002300
O	-3.67250400	-1.19535700	-0.00015100
O	-3.81160200	1.00983700	-0.00000600
H	0.77758600	2.28599000	-0.00011700
H	-1.71005200	2.20379200	0.00008500
H	-1.45319900	-2.10964700	0.00026100
N	0.34480900	-1.09904500	0.00005800

SCF Done: E(UM062X) = -626.347559766

Zero-point correction= 0.207256 (Hartree/Particle)
Thermal correction to Energy= 0.219170
Thermal correction to Enthalpy= 0.220114
Thermal correction to Gibbs Free Energy= 0.168595
Sum of electronic and zero-point Energies= -626.140304
Sum of electronic and thermal Energies= -626.128390
Sum of electronic and thermal Enthalpies= -626.127445
Sum of electronic and thermal Free Energies= -626.178964

SCRF CPCM (in DMSO): SCF Done: E(UM062X) = -626.330181714

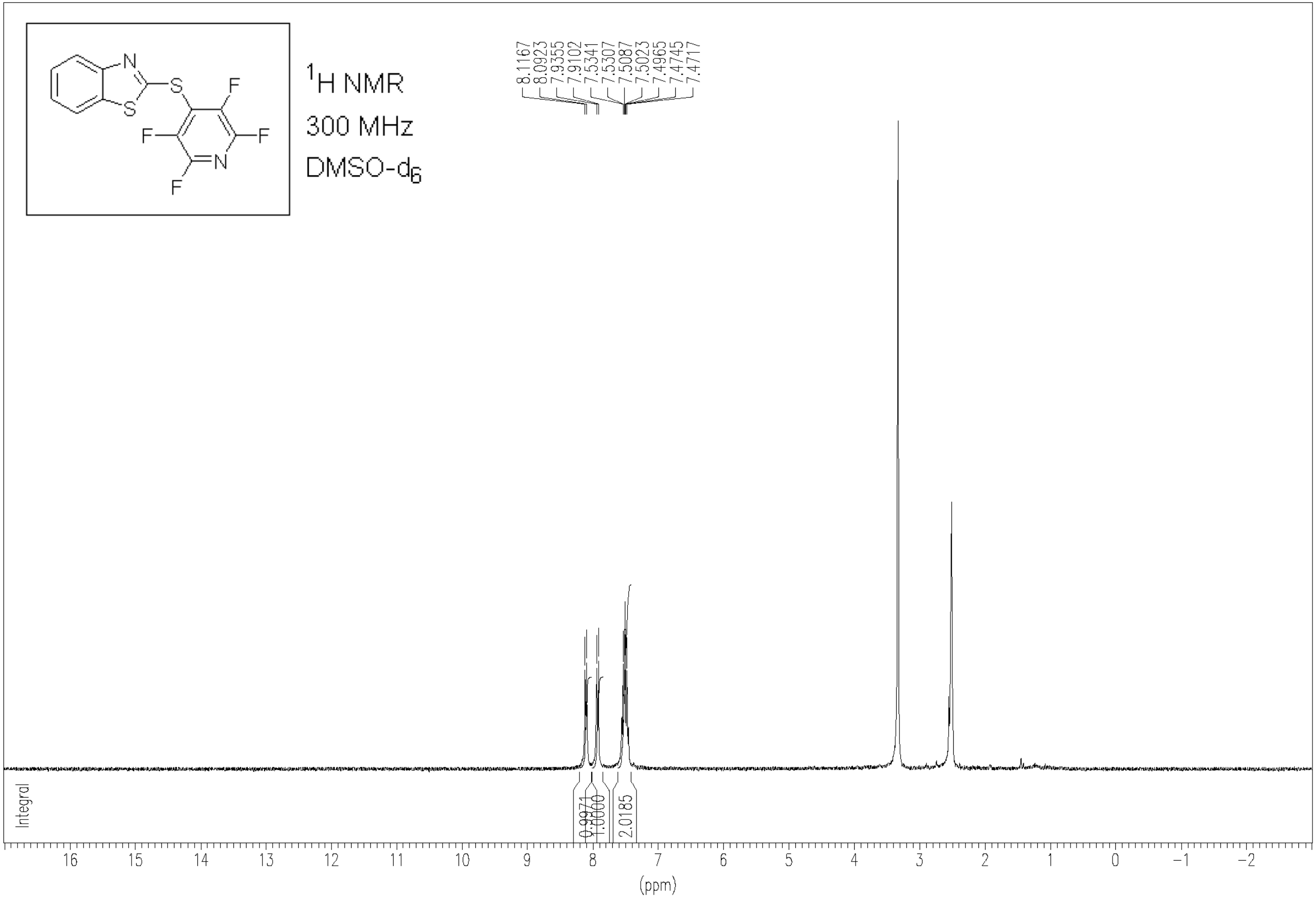

^{1}H NMR
300 MHz
DMSO-d_6
8.1167
8.0923
7.9355
7.9102
7.5341
7.5307
7.5087
7.5023
7.4965
7.4745
7.4717
0.9971
1.0000
2.0185
Integral
(ppm)

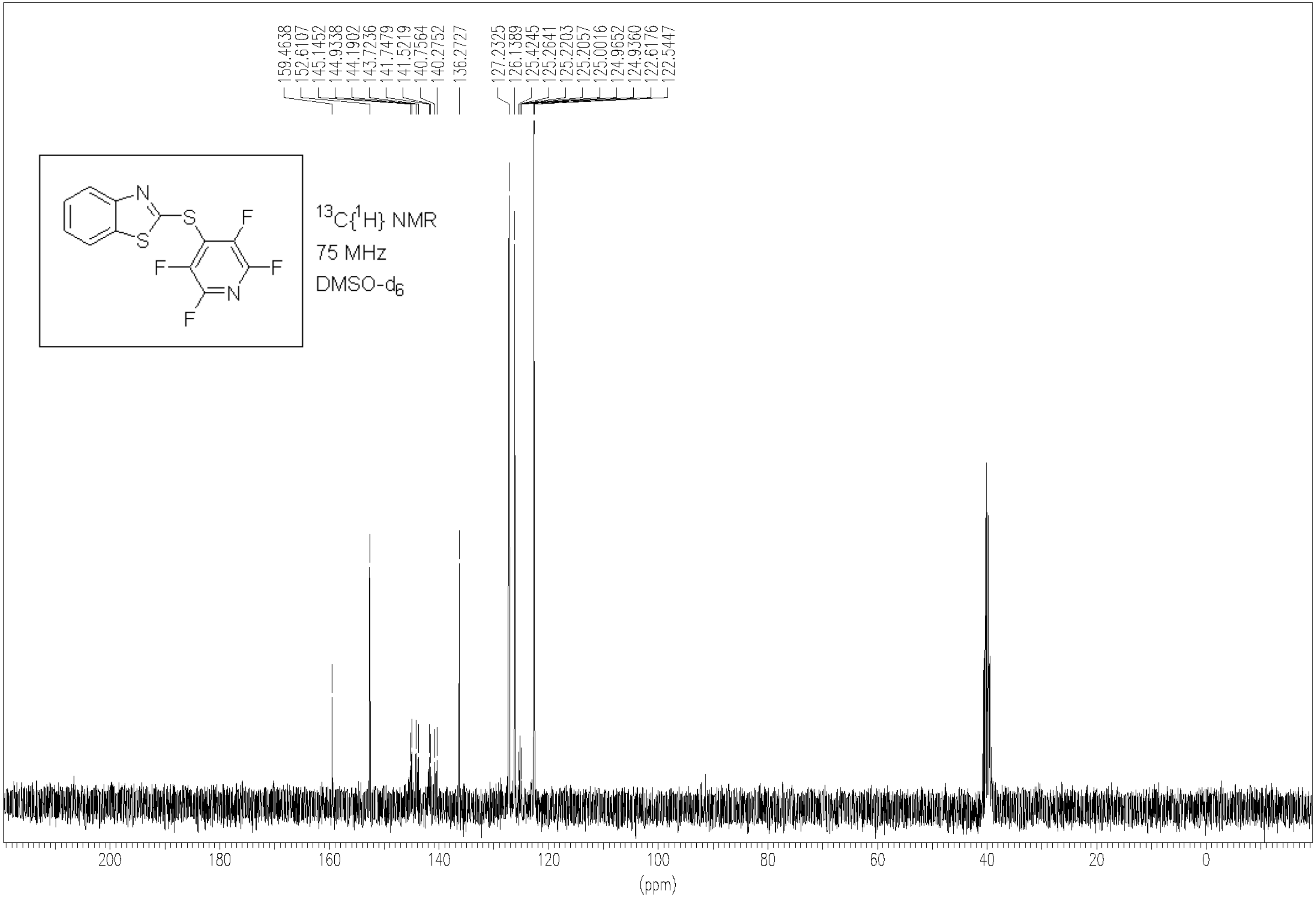

159.4638
152.6107
145.1452
144.9338
144.1902
143.7236
141.7479
141.5219
140.7564
140.2752
136.2727
127.2325
126.1389
125.4245
125.2641
125.2203
125.2057
125.0016
124.9652
124.9360
122.6176
122.5447
13C{1H} NMR
75 MHz
DMSO-d6
200
180
160
140
120
100
80
60
40
20
0
(ppm)

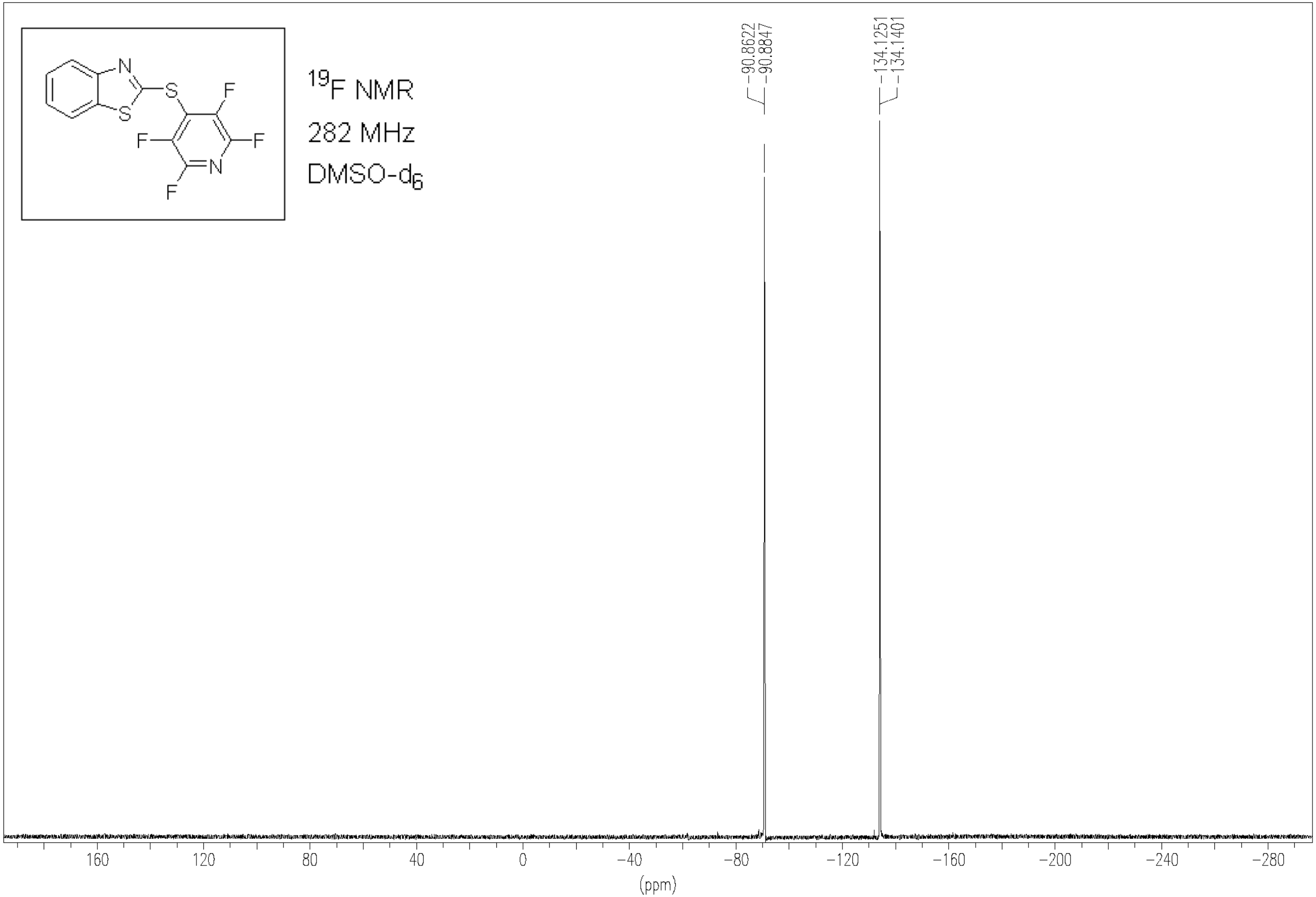

N
S
S
F
F
F
N
F
19F NMR
282 MHz
DMSO-d6
-90.8622
-90.8847
-134.1251
-134.1401
160
120
80
40
0
−40
−80
−120
−160
−200
−240
−280
(ppm)

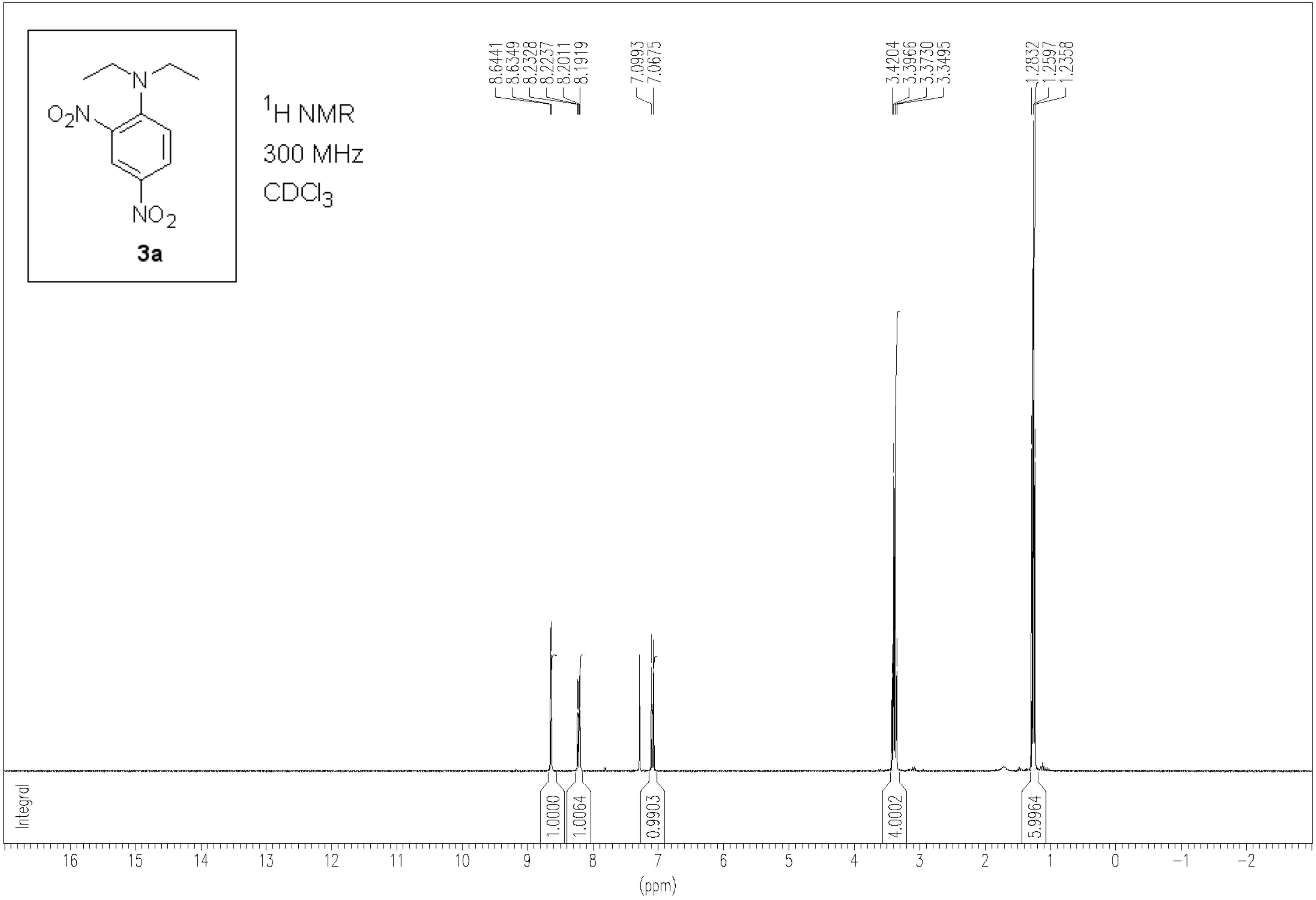

O2N
NO2
3a
1H NMR
300 MHz
CDCl3
8.6441
8.6349
8.2328
8.2237
8.2011
8.1919
7.0993
7.0675
3.4204
3.3966
3.3730
3.3495
1.2832
1.2597
1.2358
Integral
1.0000
1.0064
0.9903
4.0002
5.9964
(ppm)

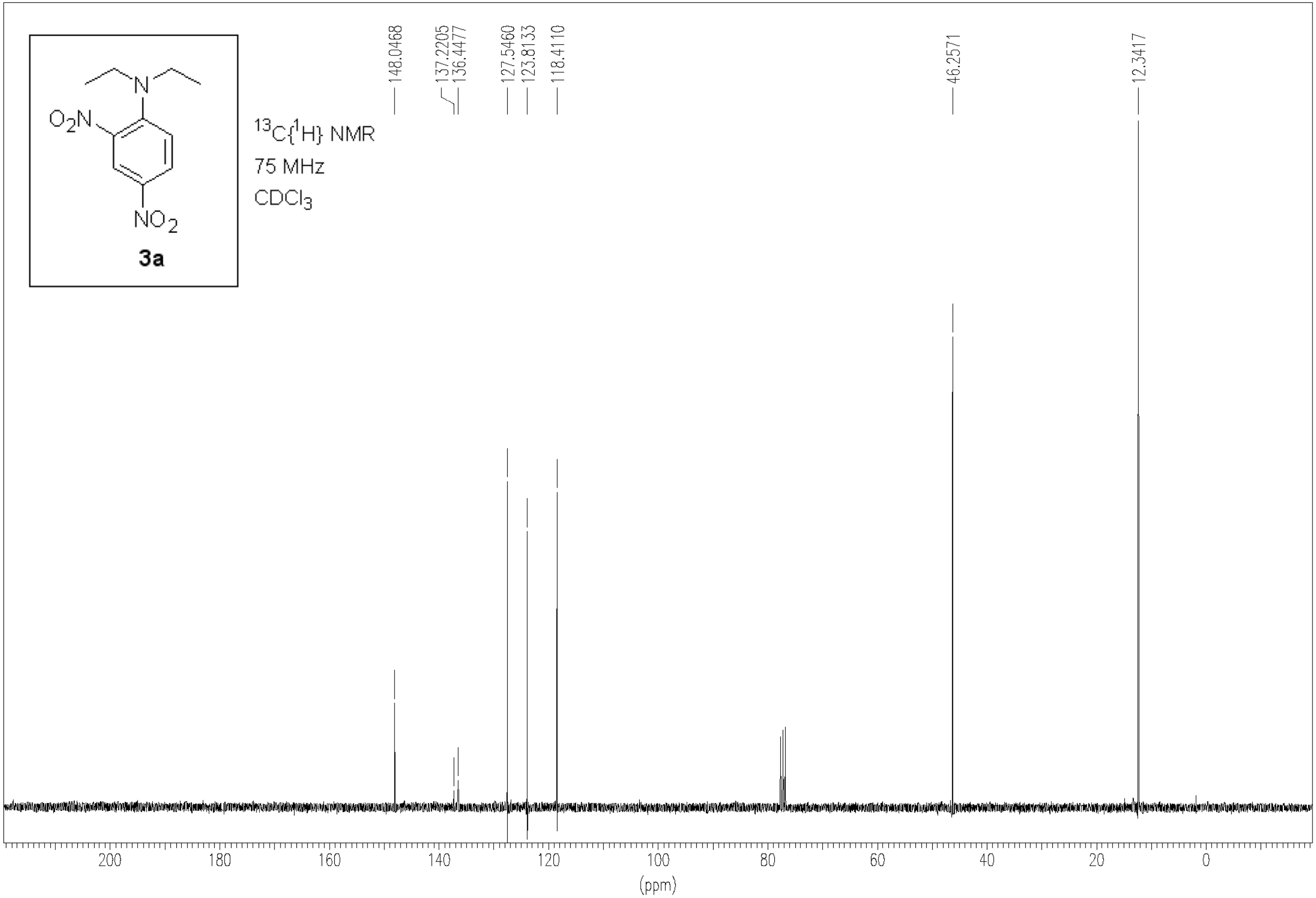

3a
$^{13}C\{^1H\}$ NMR
75 MHz
$CDCl_3$
148.0468
137.2205
136.4477
127.5460
123.8133
118.4110
46.2571
12.3417
200
180
160
140
120
100
80
60
40
20
0
(ppm)

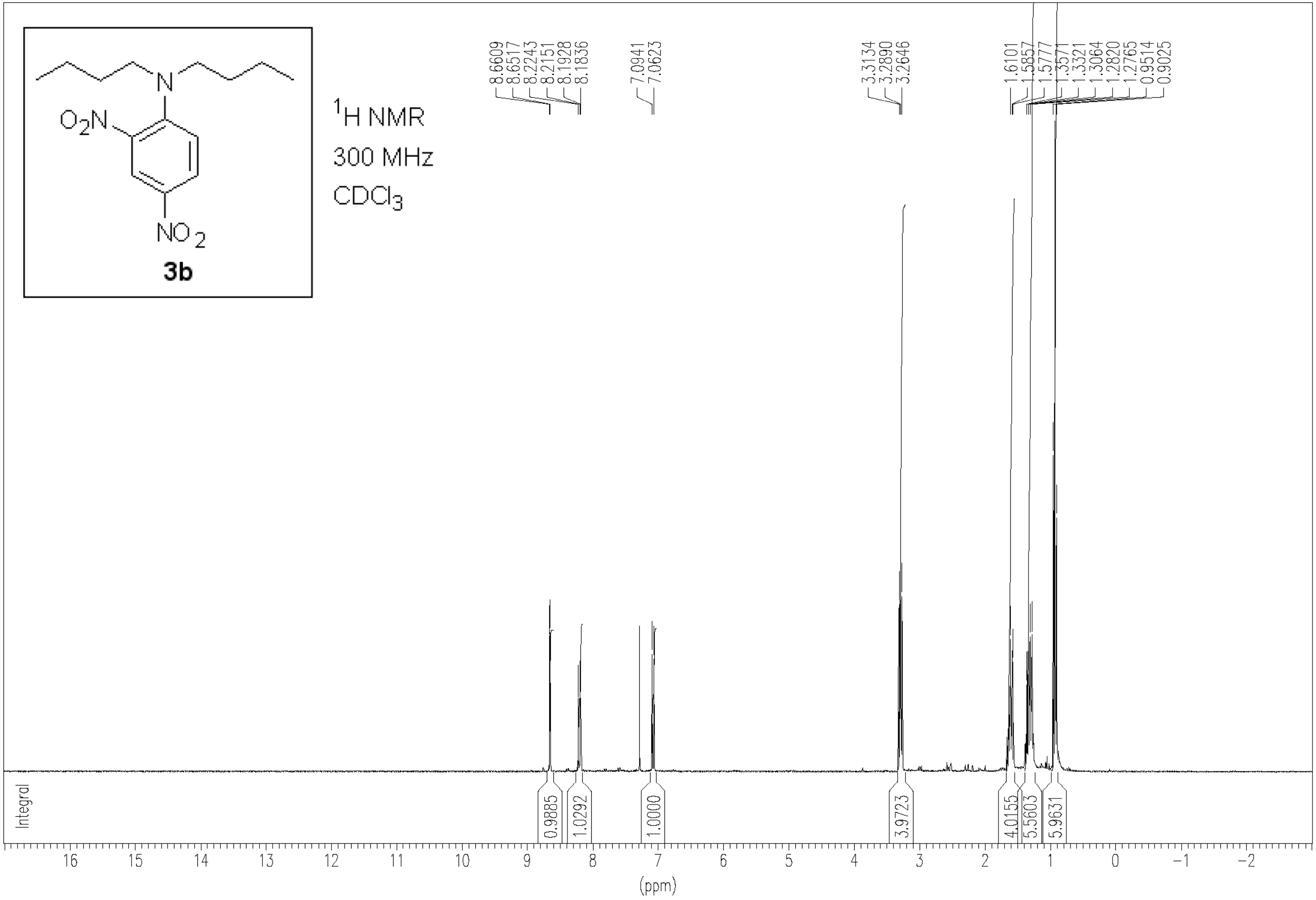

3b
1H NMR
300 MHz
CDCl3
8.6609
8.6517
8.2243
8.2151
8.1928
8.1836
7.0941
7.0623
3.3134
3.2890
3.2646
1.6101
1.5857
1.5777
1.3571
1.3321
1.3064
1.2820
1.2765
0.9514
0.9025
Integral
0.9885
1.0292
1.0000
3.9723
4.0155
5.5603
5.9631
(ppm)

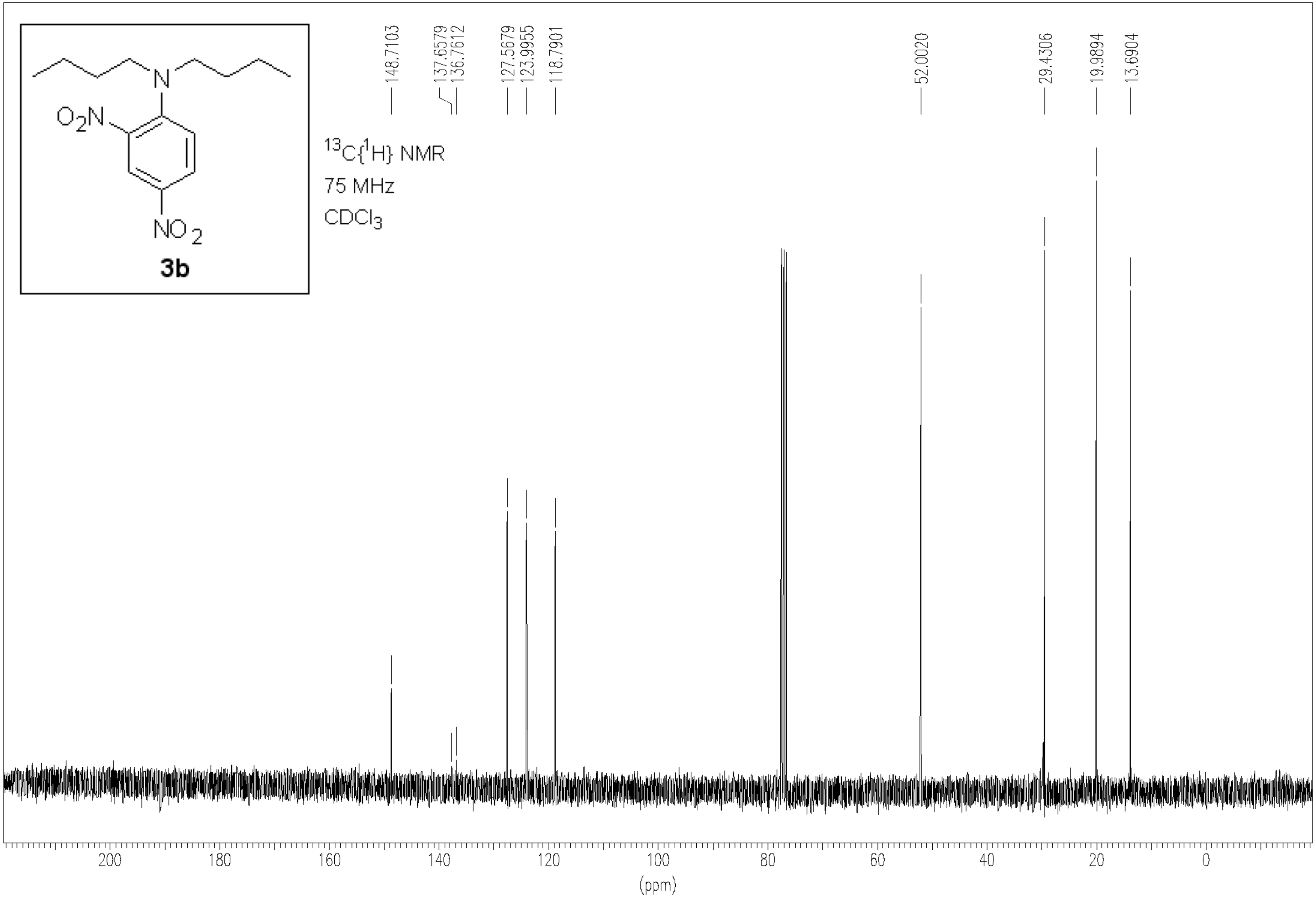

O_2N
N
NO_2
3b
$^{13}C\{^{1}H\}$ NMR
75 MHz
$CDCl_3$
148.7103
137.6579
136.7612
127.5679
123.9955
118.7901
52.0020
29.4306
19.9894
13.6904
200
180
160
140
120
100
80
60
40
20
0
(ppm)

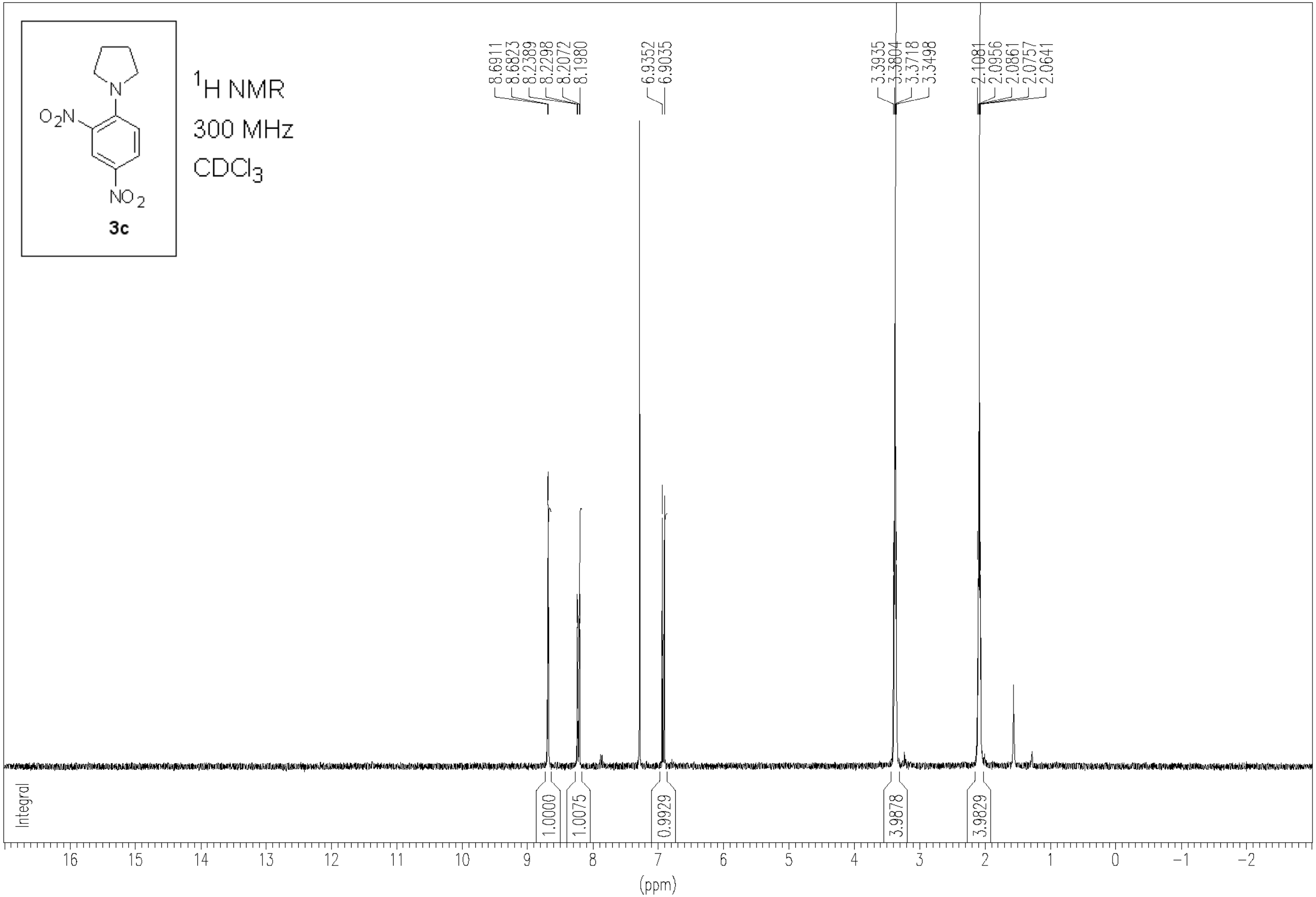

3c
1H NMR
300 MHz
CDCl3
8.6911
8.6823
8.2389
8.2298
8.2072
8.1980
6.9352
6.9035
3.3935
3.3804
3.3718
3.3498
2.1081
2.0956
2.0861
2.0757
2.0641
Integral
1.0000
1.0075
0.9929
3.9878
3.9829
(ppm)

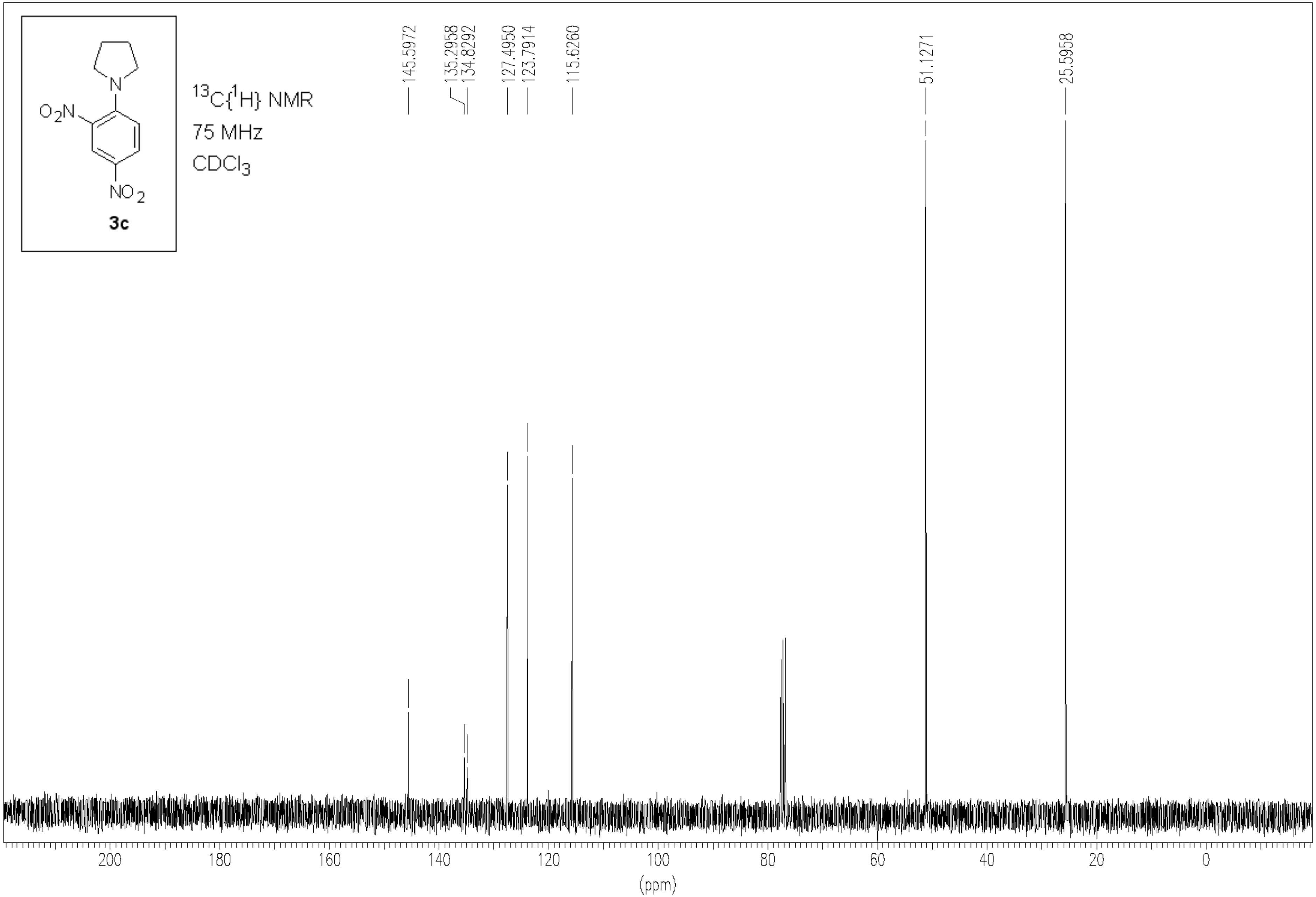

3c
$^{13}C\{^1H\}$ NMR
75 MHz
$CDCl_3$
145.5972
135.2958
134.8292
127.4950
123.7914
115.6260
51.1271
25.5958
200
180
160
140
120
100
80
60
40
20
0
(ppm)

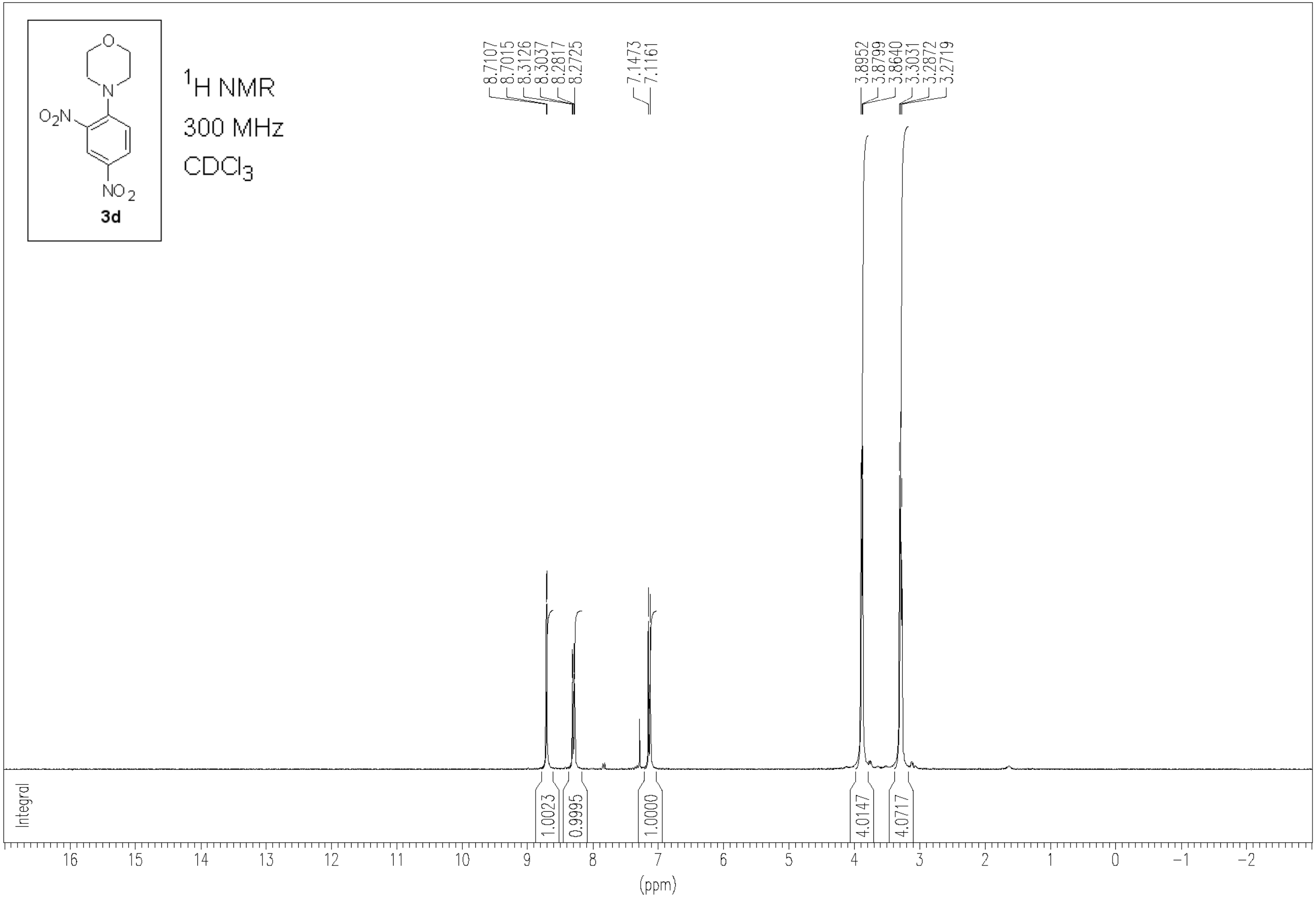

O
N
O_2N
NO_2
3d
^1H NMR
300 MHz
$CDCl_3$
8.7107
8.7015
8.3126
8.3037
8.2817
8.2725
7.1473
7.1161
3.8952
3.8799
3.8640
3.3031
3.2872
3.2719
Integral
1.0023
0.9995
1.0000
4.0147
4.0717
16 15 14 13 12 11 10 9 8 7 6 5 4 3 2 1 0 −1 −2
(ppm)

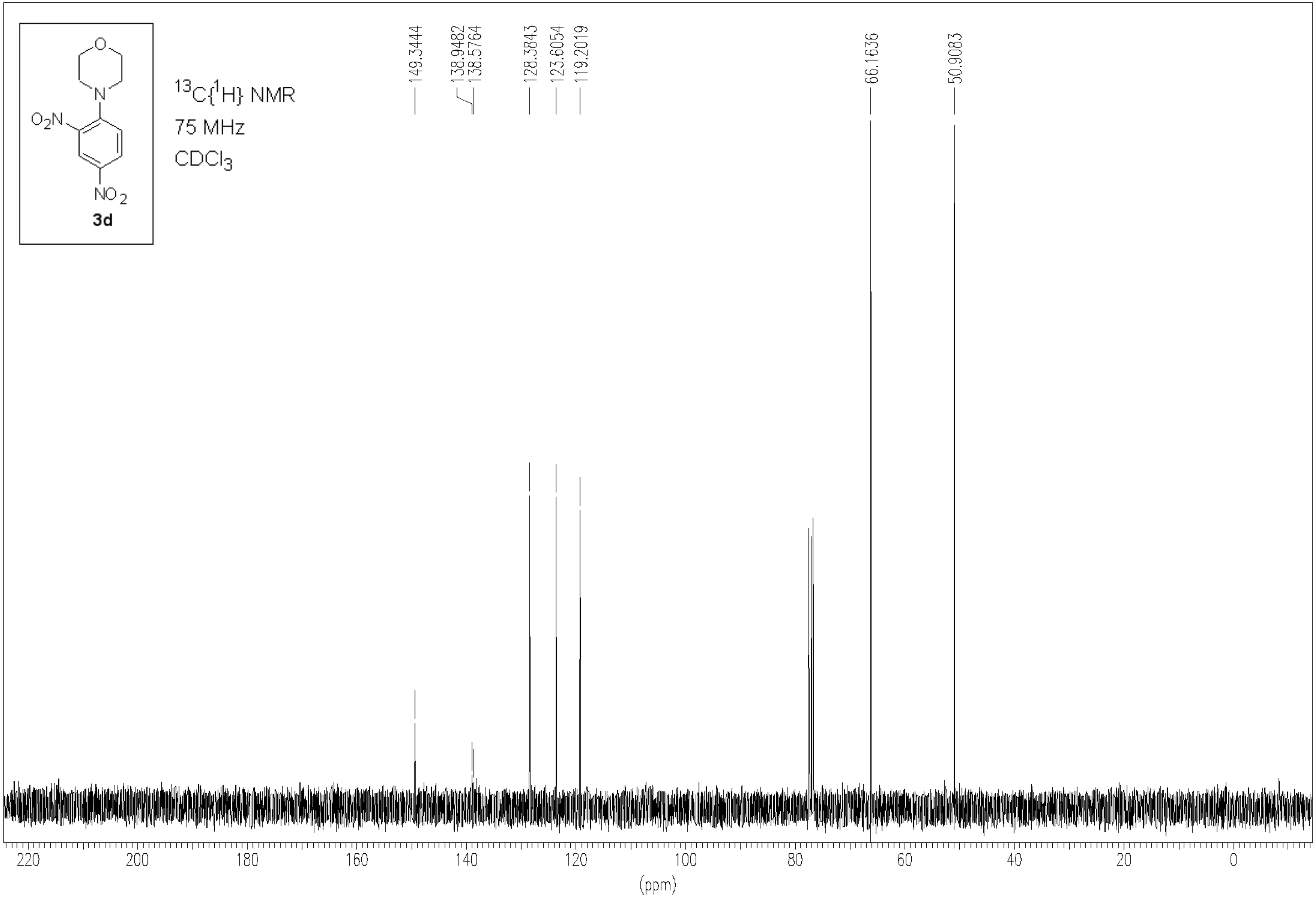
3d
13C{1H} NMR
75 MHz
CDCl3
149.3444
138.9482
138.5764
128.3843
123.6054
119.2019
66.1636
50.9083
220
200
180
160
140
120
100
80
60
40
20
0
(ppm)

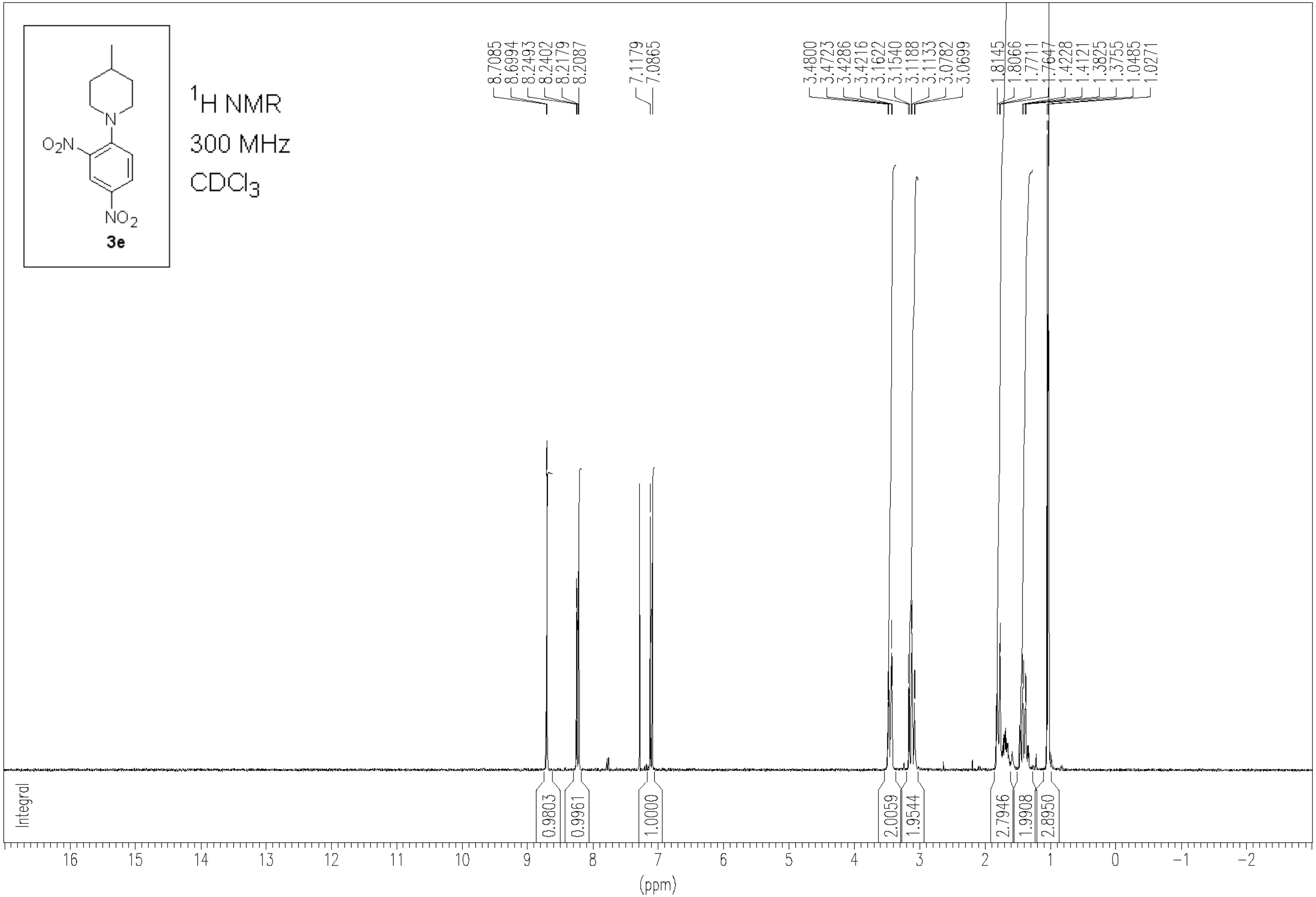
3e
1H NMR
300 MHz
CDCl3
8.7085
8.6994
8.2493
8.2402
8.2179
8.2087
7.1179
7.0865
3.4800
3.4723
3.4286
3.4216
3.1622
3.1540
3.1188
3.1133
3.0782
3.0699
1.8145
1.8066
1.7711
1.7647
1.4228
1.4121
1.3825
1.3755
1.0485
1.0271
Integral
0.9803
0.9961
1.0000
2.0059
1.9544
2.7946
1.9908
2.8950
(ppm)

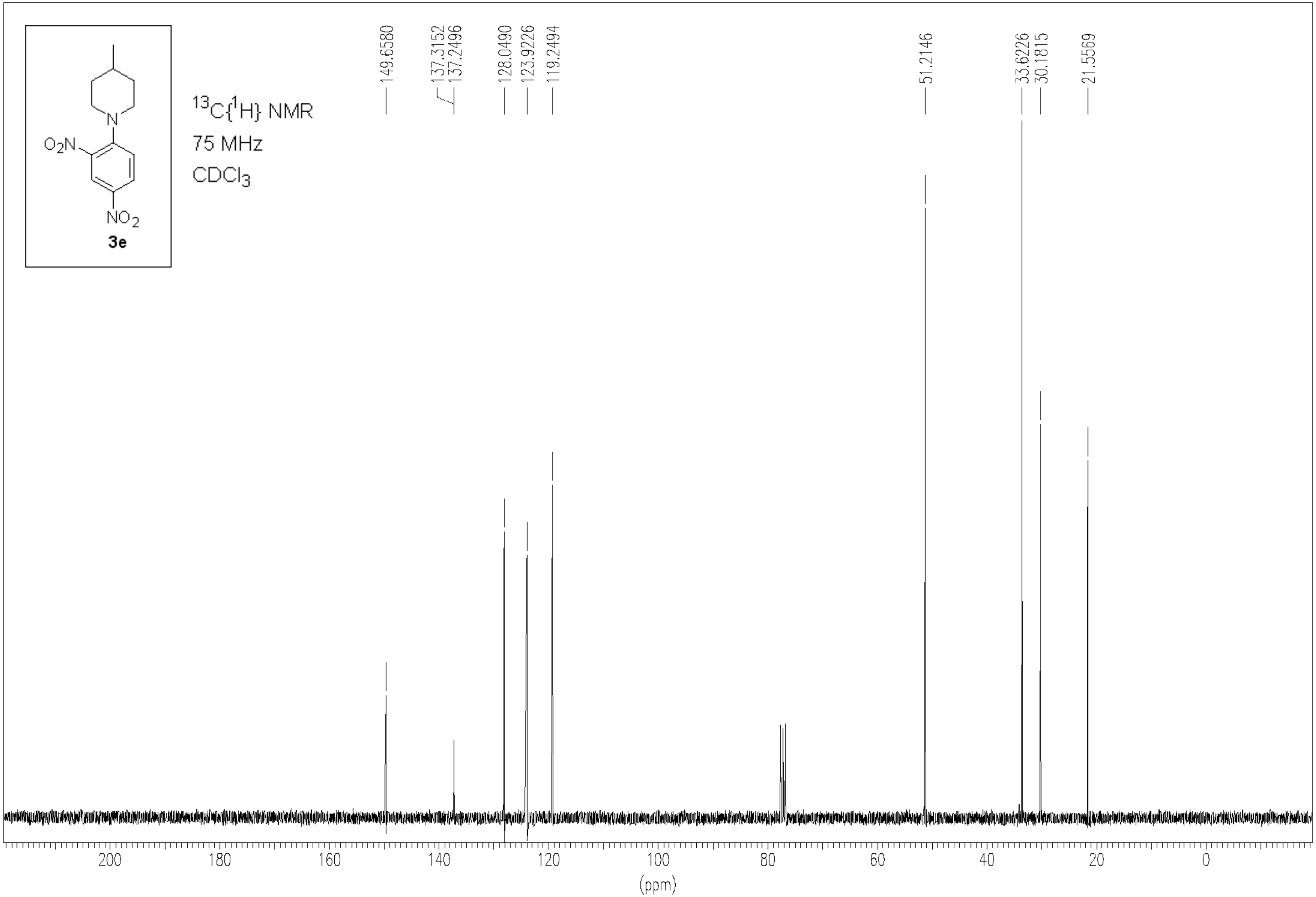

3e
$^{13}C\{^1H\}$ NMR
75 MHz
$CDCl_3$
149.6580
137.3152
137.2496
128.0490
123.9226
119.2494
51.2146
33.6226
30.1815
21.5569
200
180
160
140
120
100
80
60
40
20
0
(ppm)

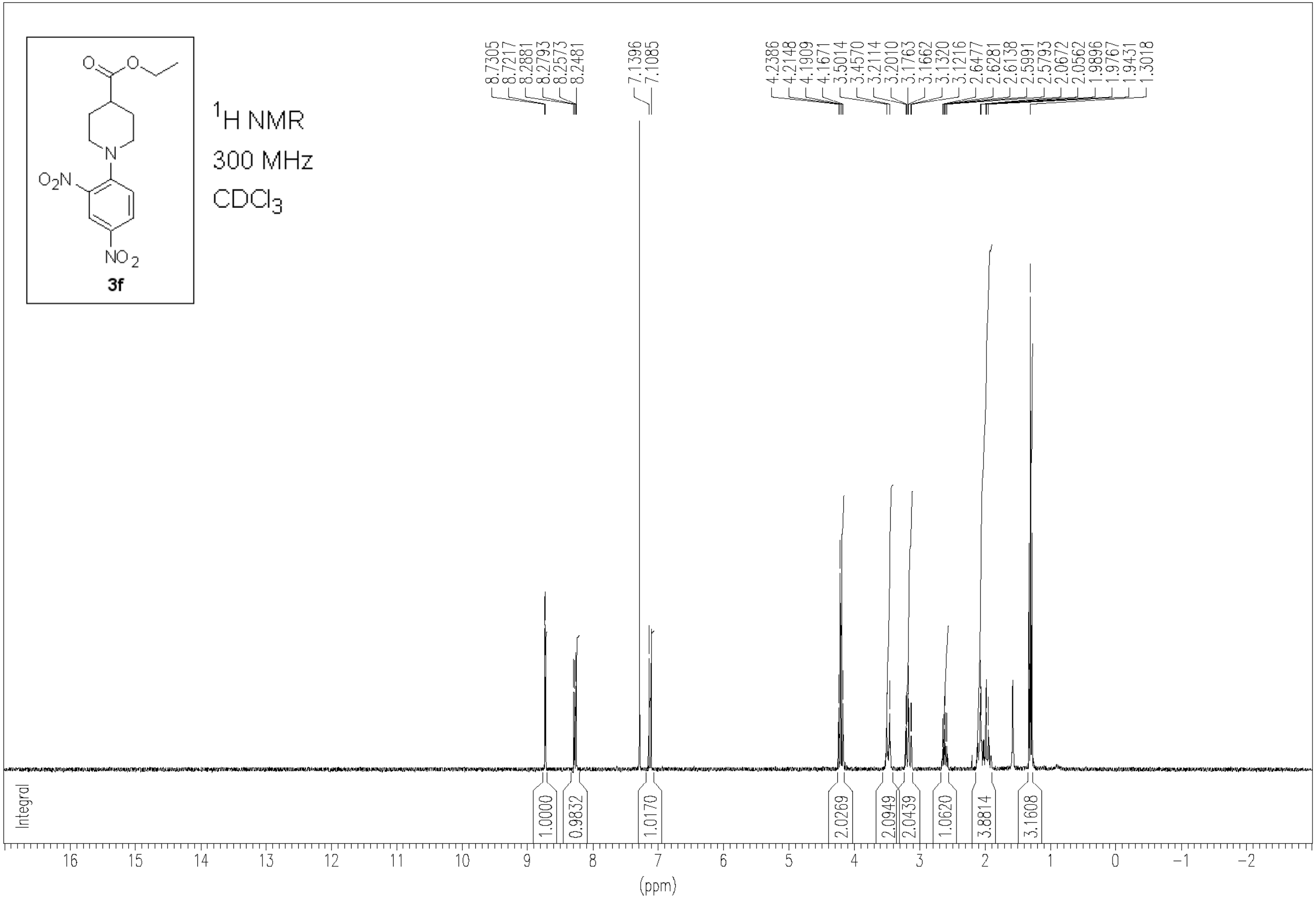

3f
1H NMR
300 MHz
CDCl3
8.7305
8.7217
8.2881
8.2793
8.2573
8.2481
7.1396
7.1085
4.2386
4.2148
4.1909
4.1671
3.5014
3.4570
3.2114
3.2010
3.1763
3.1662
3.1320
3.1216
2.6477
2.6281
2.6138
2.5991
2.5793
2.0672
2.0562
1.9896
1.9767
1.9431
1.3018
Integral
1.0000
0.9832
1.0170
2.0269
2.0949
2.0439
1.0620
3.8814
3.1608
16 15 14 13 12 11 10 9 8 7 6 5 4 3 2 1 0 −1 −2
(ppm)

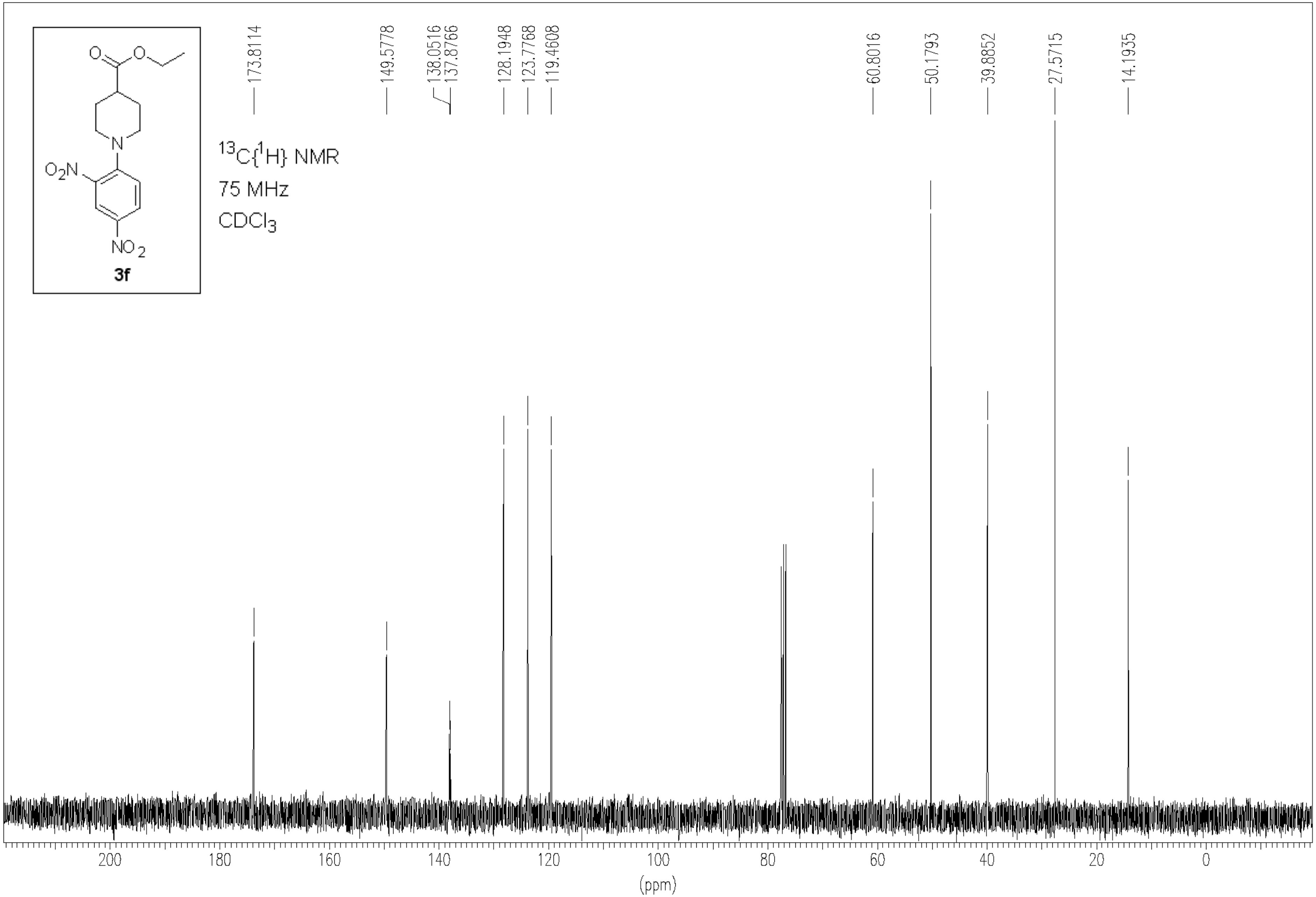

3f
13C{1H} NMR
75 MHz
CDCl3
173.8114
149.5778
138.0516
137.8766
128.1948
123.7768
119.4608
60.8016
50.1793
39.8852
27.5715
14.1935
200
180
160
140
120
100
80
60
40
20
0
(ppm)

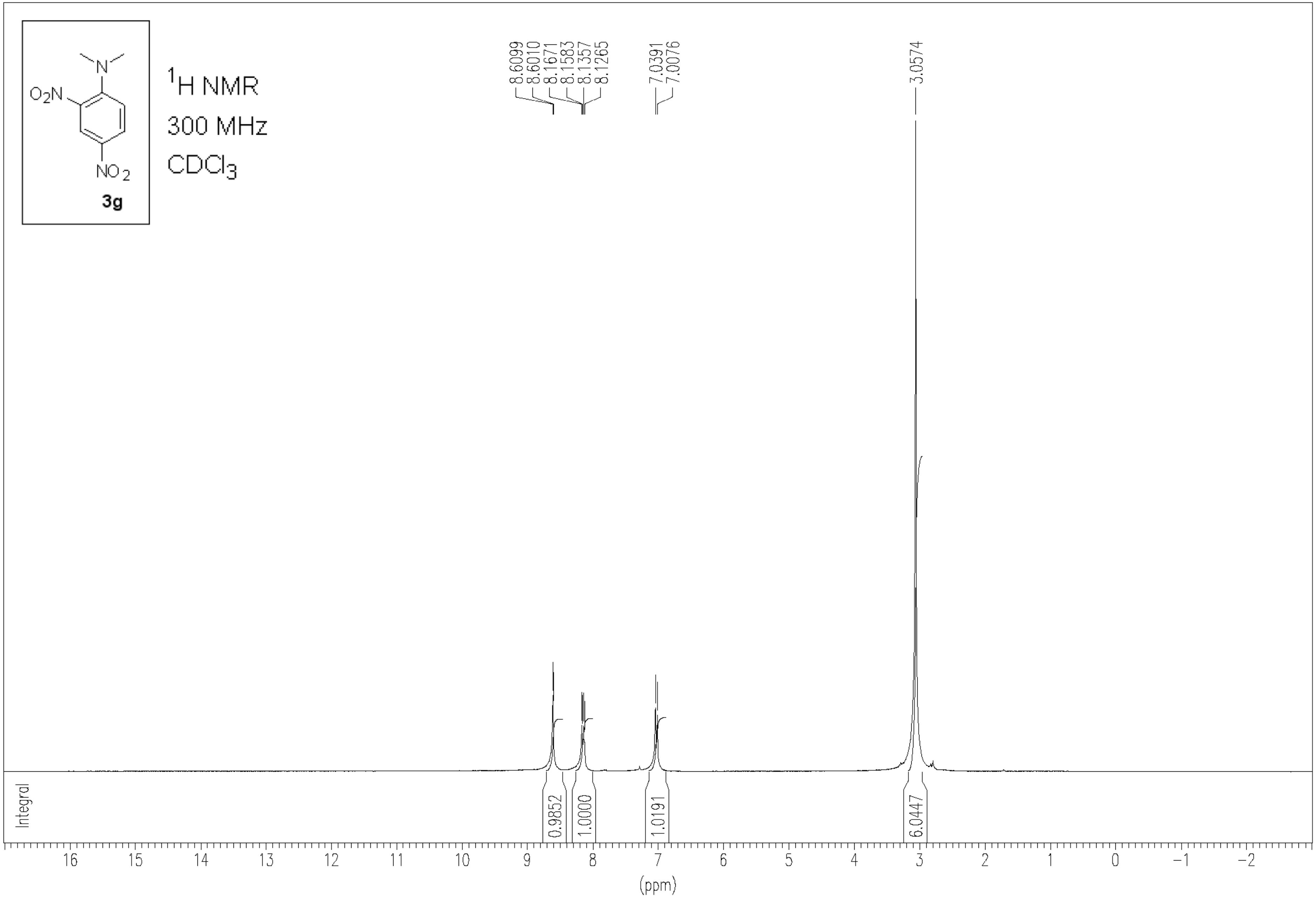

O_2N
N
NO_2
3g
^1H NMR
300 MHz
$CDCl_3$
8.6099
8.6010
8.1671
8.1583
8.1357
8.1265
7.0391
7.0076
3.0574
Integral
0.9852
1.0000
1.0191
6.0447
16 15 14 13 12 11 10 9 8 7 6 5 4 3 2 1 0 −1 −2
(ppm)

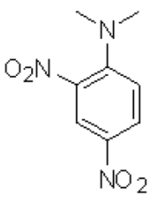

3g

$^{13}C\{^1H\}$ NMR
75 MHz
$CDCl_3$

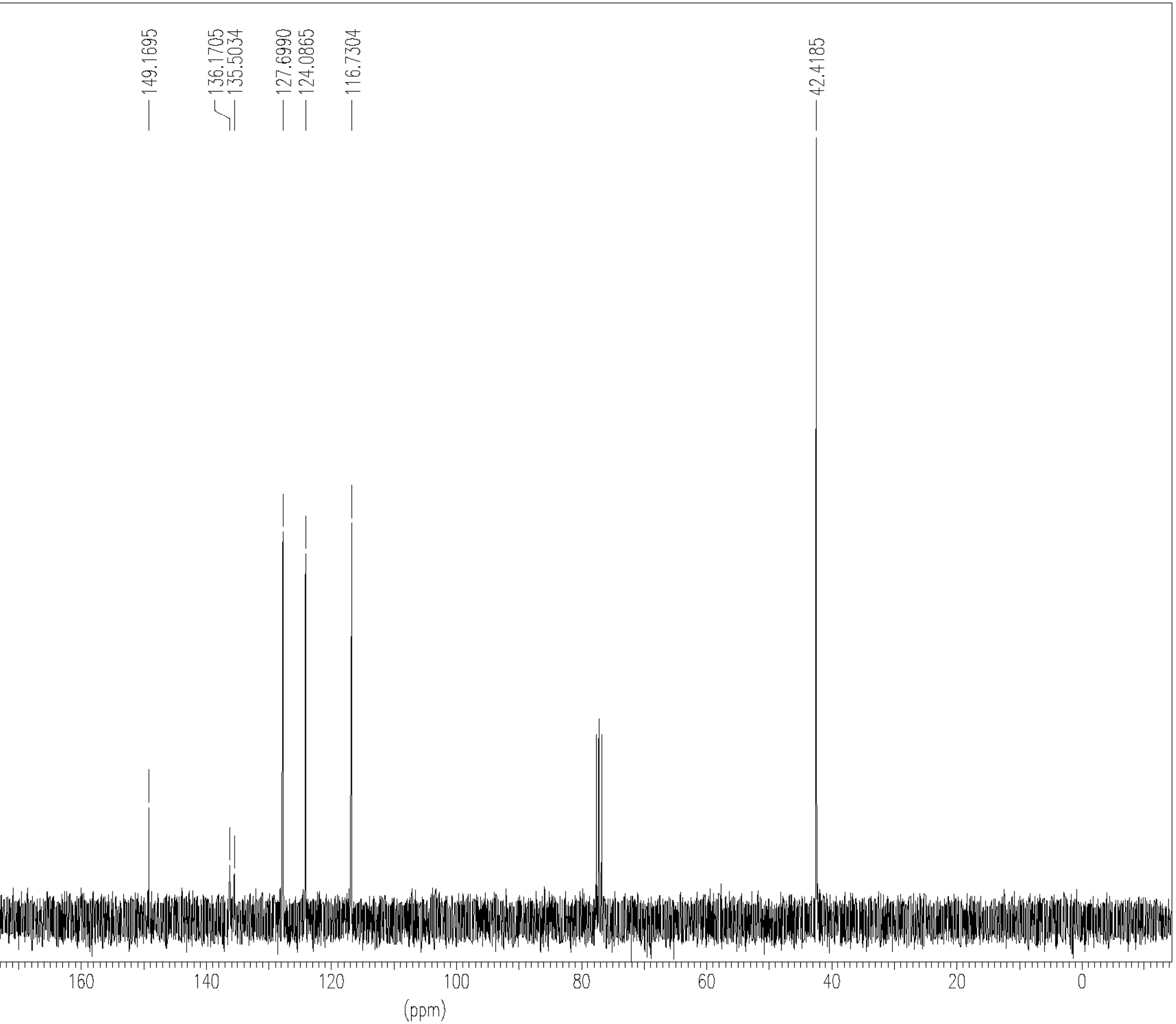

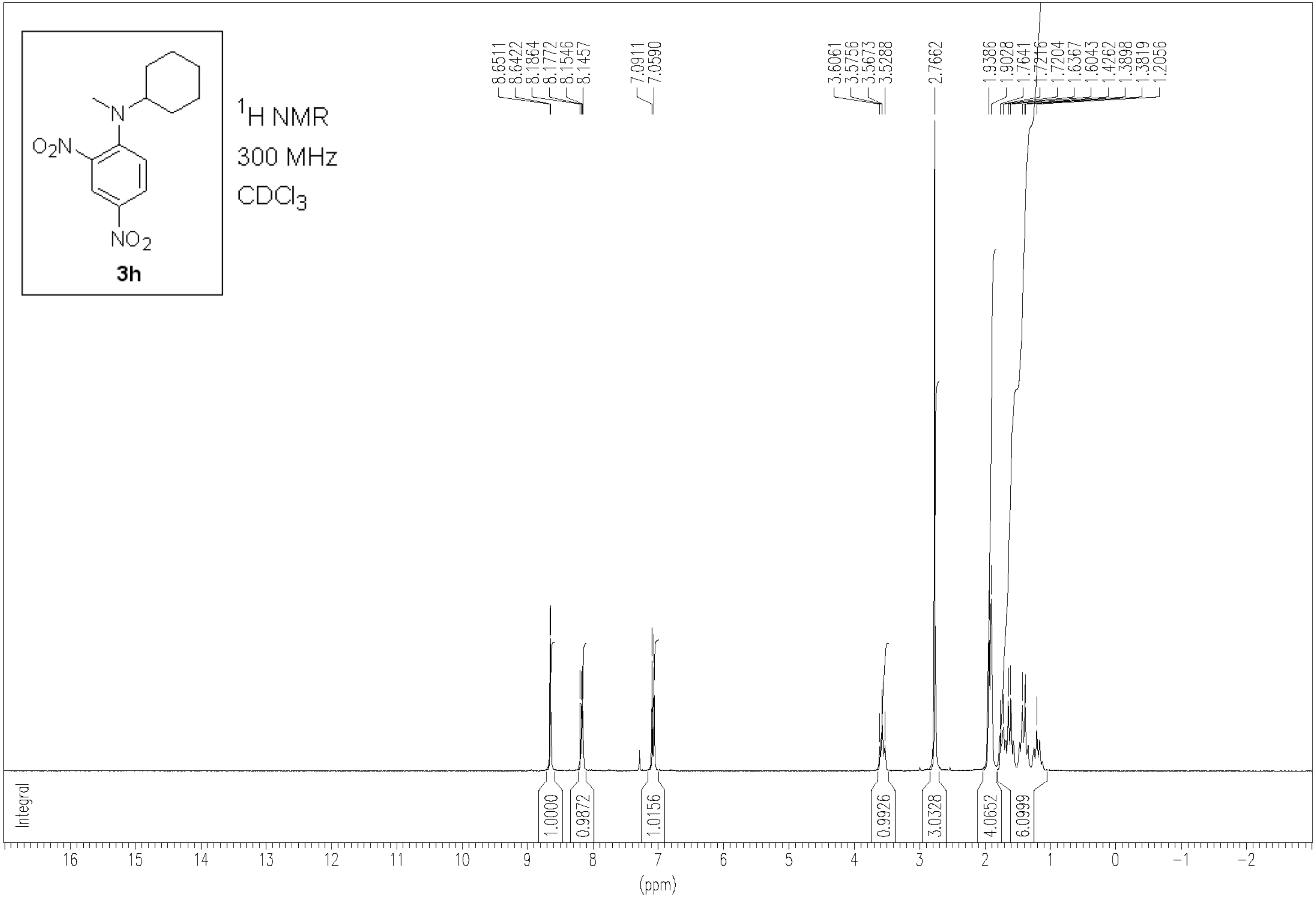
3h
1H NMR
300 MHz
CDCl3
8.6511
8.6422
8.1864
8.1772
8.1546
8.1457
7.0911
7.0590
3.6061
3.5756
3.5673
3.5288
2.7662
1.9386
1.9028
1.7641
1.7216
1.7204
1.6367
1.6043
1.4262
1.3898
1.3819
1.2056
Integral
1.0000
0.9872
1.0156
0.9926
3.0328
4.0652
6.0999
(ppm)

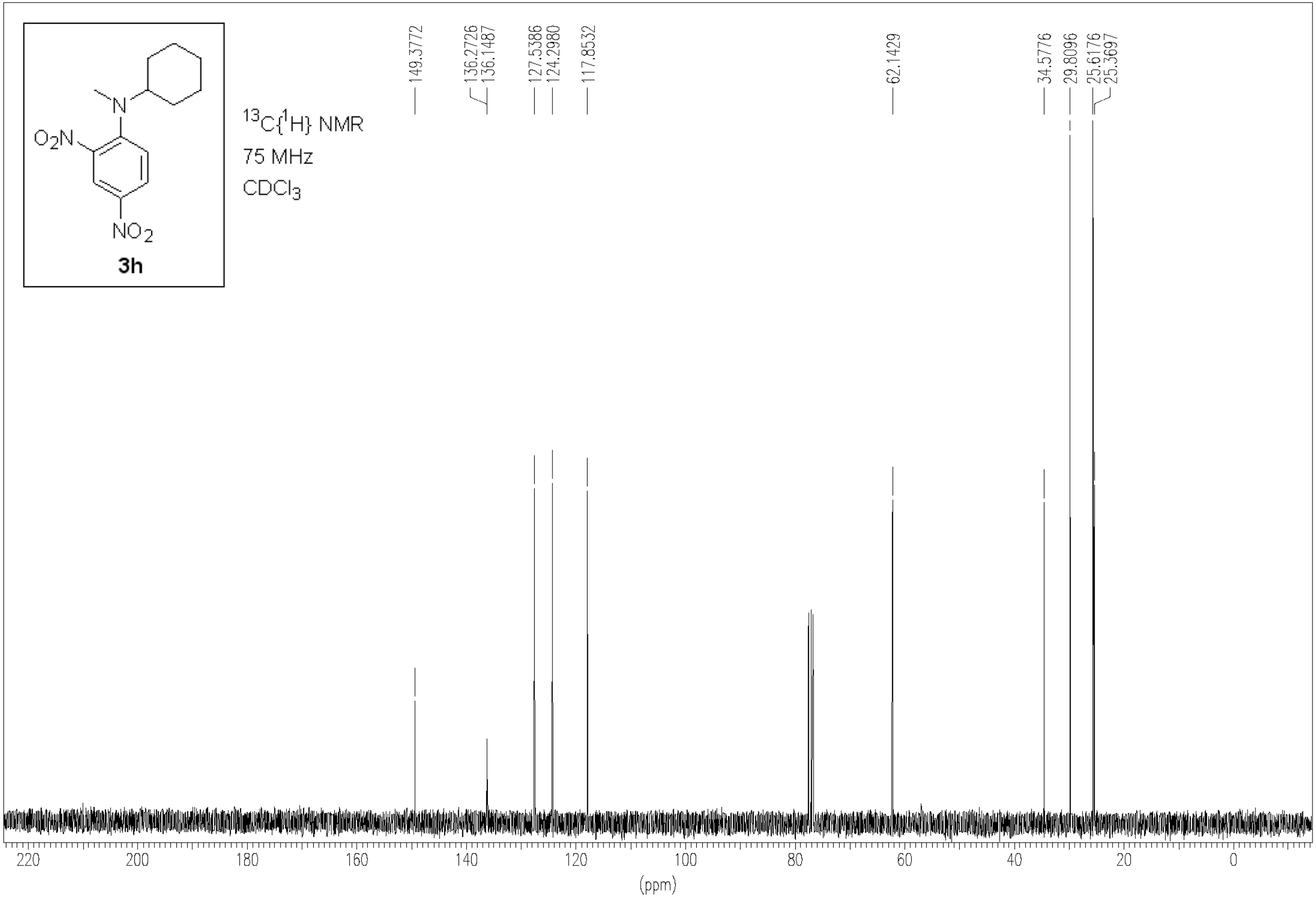

3h
13C{1H} NMR
75 MHz
CDCl3
149.3772
136.2726
136.1487
127.5386
124.2980
117.8532
62.1429
34.5776
29.8096
25.6176
25.3697
220
200
180
160
140
120
100
80
60
40
20
0
(ppm)

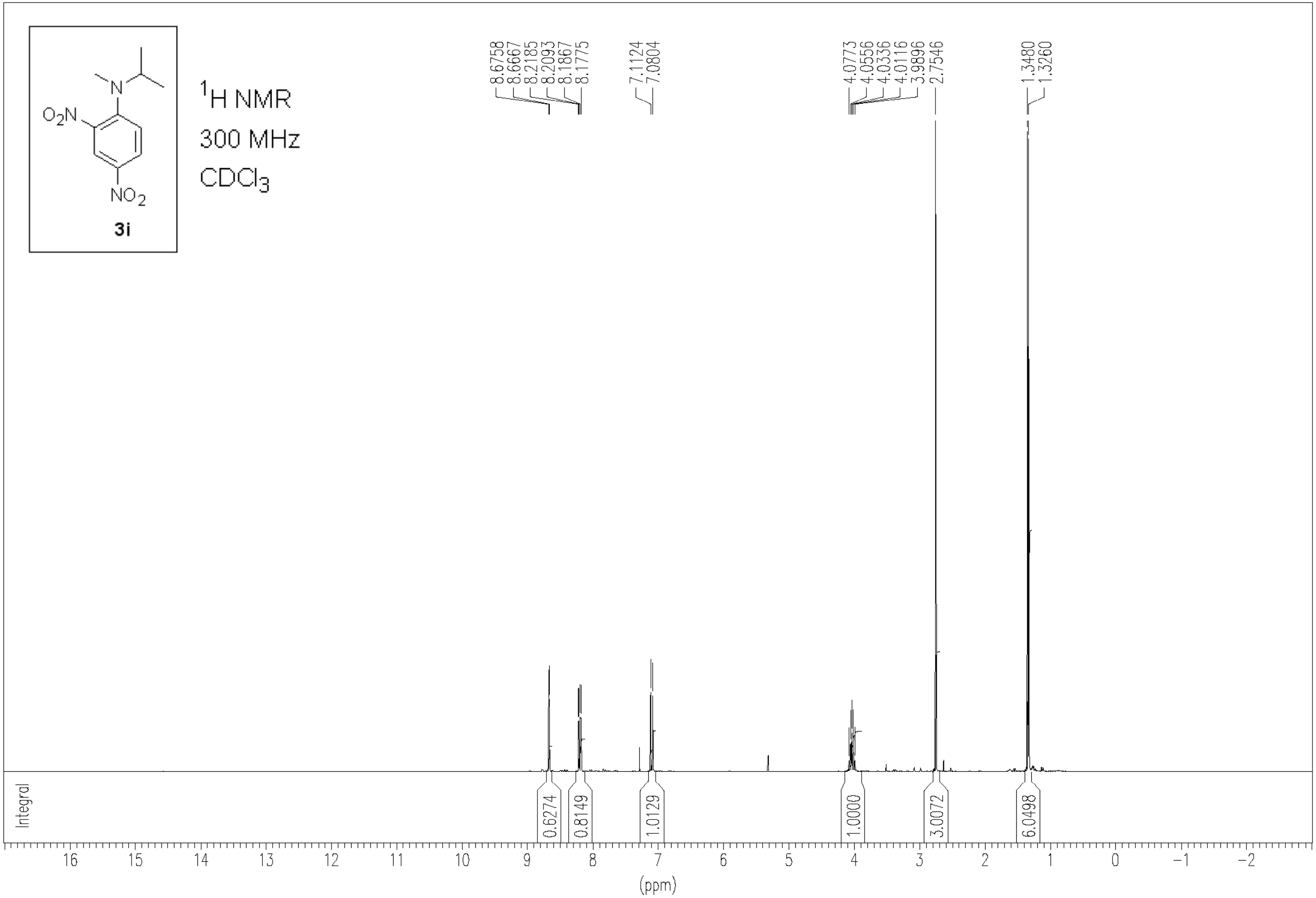

3i
1H NMR
300 MHz
CDCl3
8.6758
8.6667
8.2185
8.2093
8.1867
8.1775
7.1124
7.0804
4.0773
4.0556
4.0336
4.0116
3.9896
2.7546
1.3480
1.3260
Integral
0.6274
0.8149
1.0129
1.0000
3.0072
6.0498
(ppm)

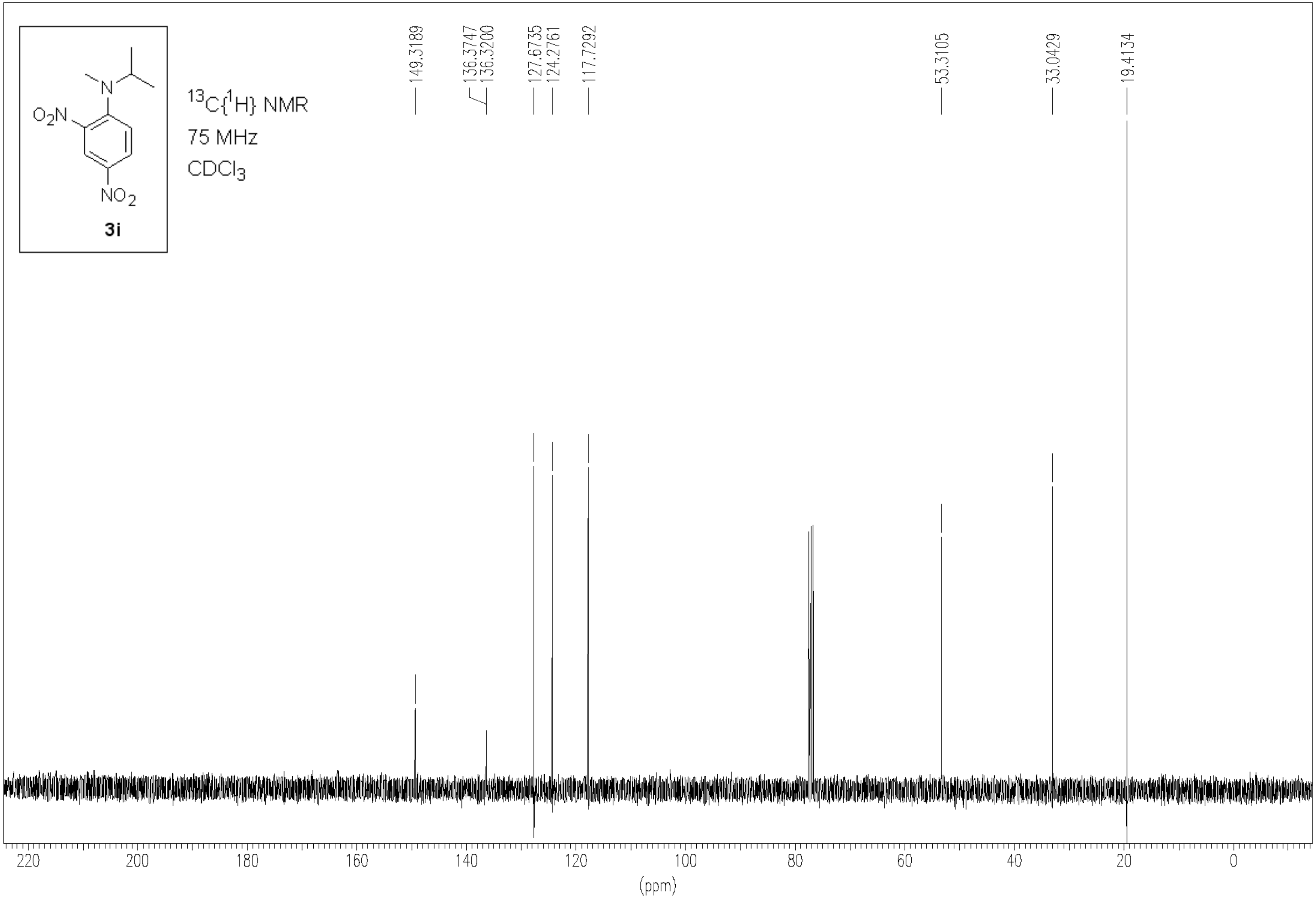
O2N
NO2
3i
13C{1H} NMR
75 MHz
CDCl3
149.3189
136.3747
136.3200
127.6735
124.2761
117.7292
53.3105
33.0429
19.4134
220
200
180
160
140
120
100
80
60
40
20
0
(ppm)

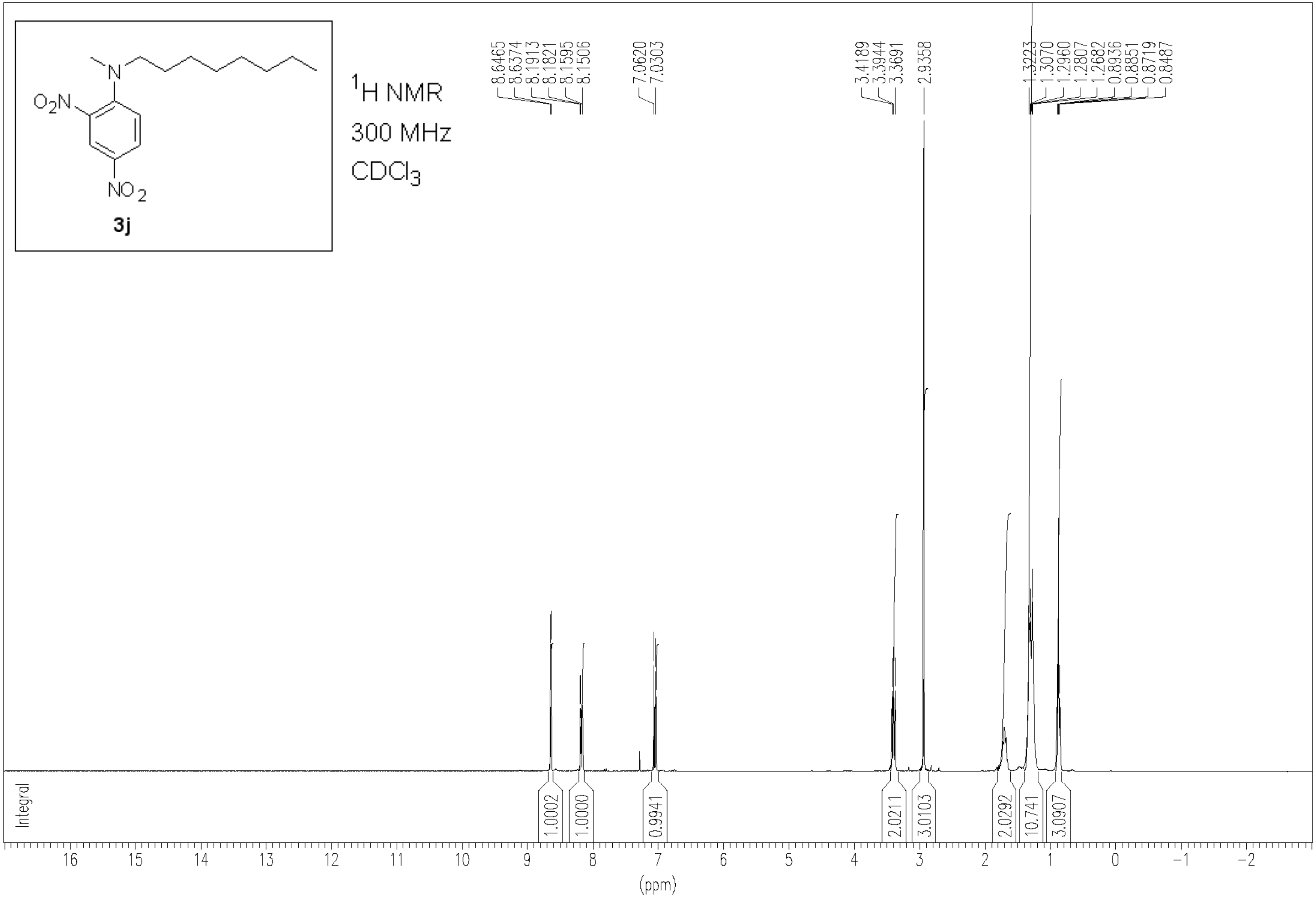

O_2N
N
NO_2
3j
1H NMR
300 MHz
$CDCl_3$
8.6465
8.6374
8.1913
8.1821
8.1595
8.1506
7.0620
7.0303
3.4189
3.3944
3.3691
2.9358
1.3223
1.3070
1.2960
1.2807
1.2682
0.8936
0.8851
0.8719
0.8487
Integral
1.0002
1.0000
0.9941
2.0211
3.0103
2.0292
10.741
3.0907
16 15 14 13 12 11 10 9 8 7 6 5 4 3 2 1 0 −1 −2
(ppm)

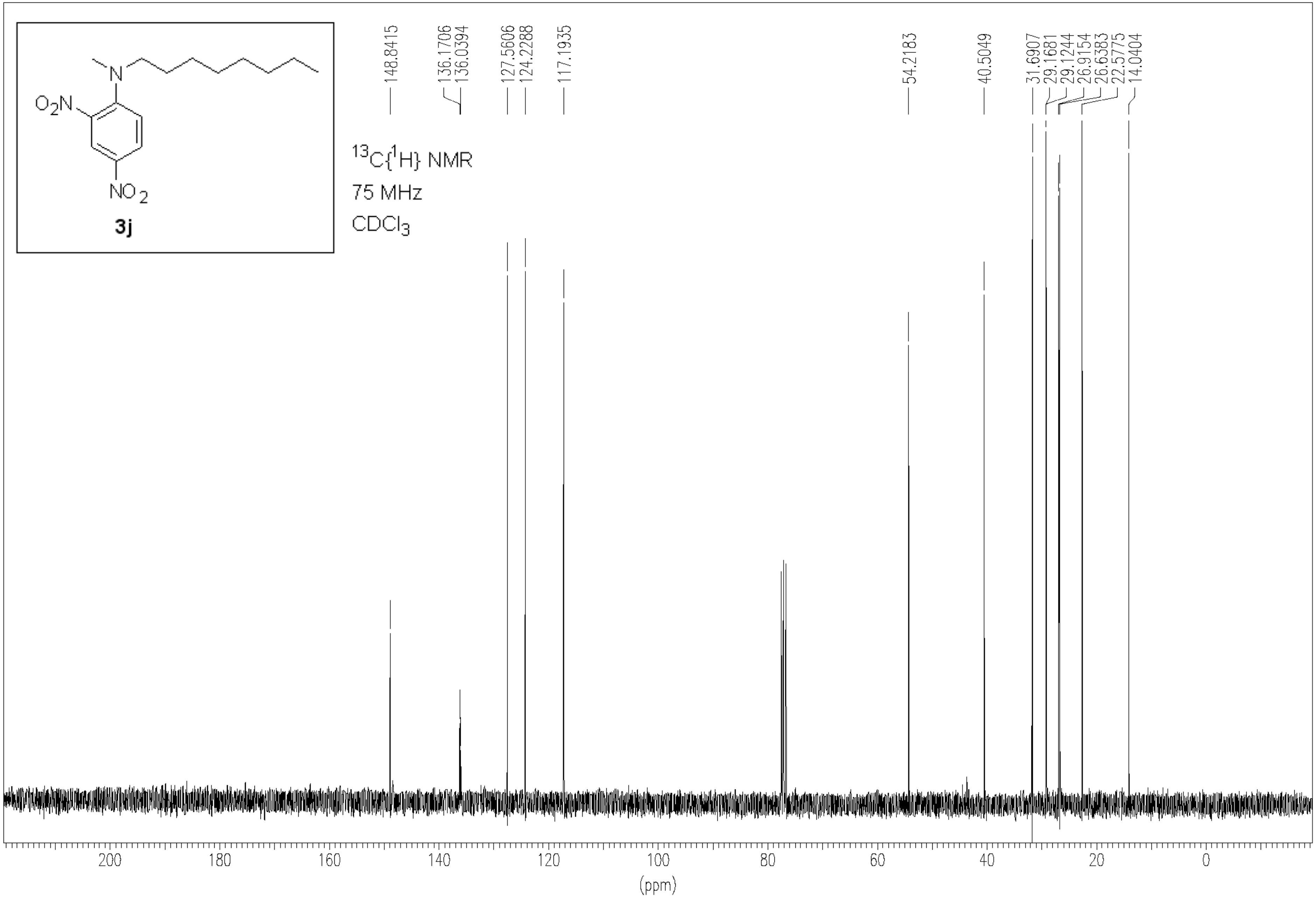

O2N
NO2
3j
13C{1H} NMR
75 MHz
CDCl3
148.8415
136.1706
136.0394
127.5606
124.2288
117.1935
54.2183
40.5049
31.6907
29.1681
29.1244
26.9154
26.6383
22.5775
14.0404
200
180
160
140
120
100
80
60
40
20
0
(ppm)

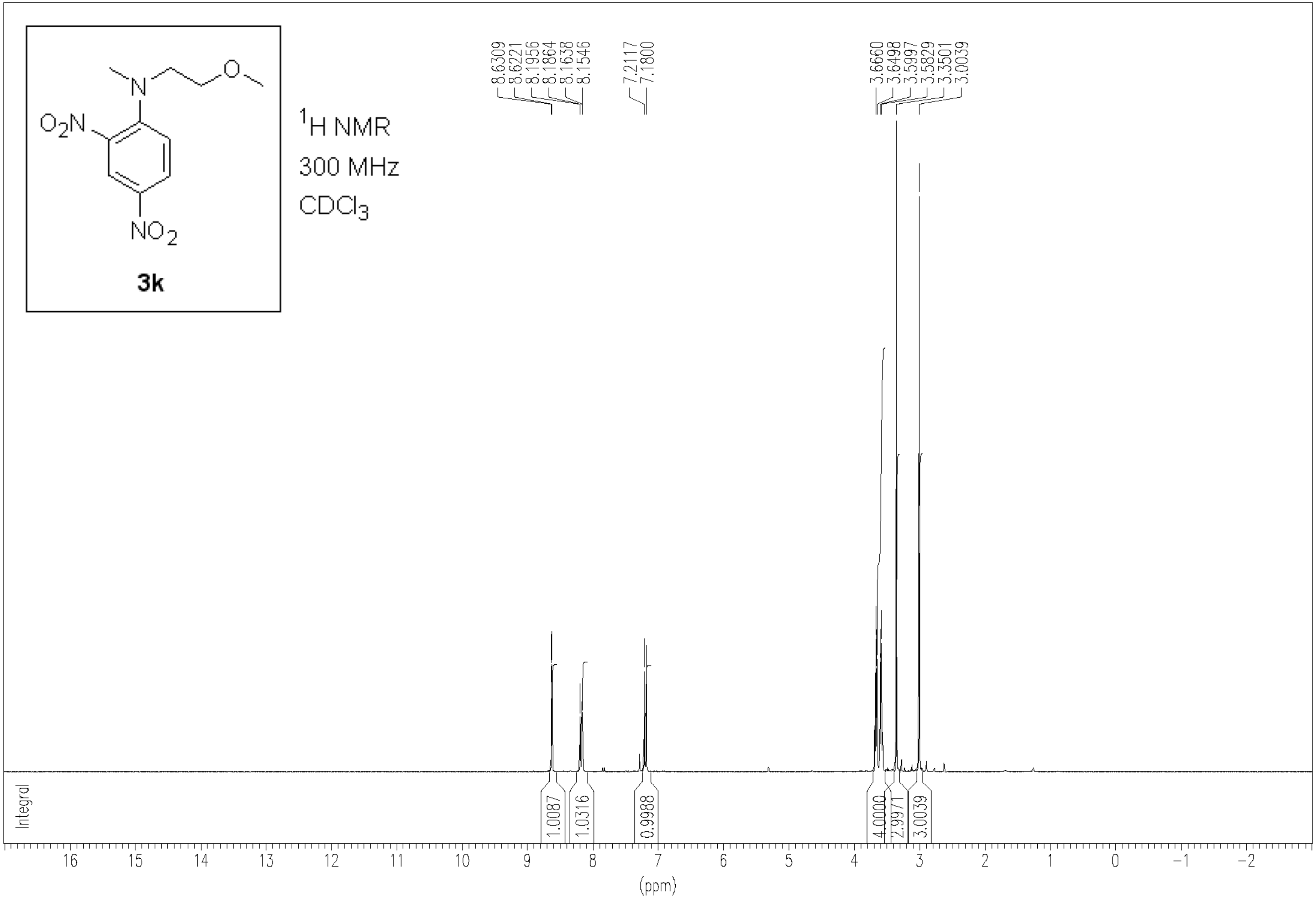

O2N
NO2
3k
1H NMR
300 MHz
CDCl3
8.6309
8.6221
8.1956
8.1864
8.1638
8.1546
7.2117
7.1800
3.6660
3.6498
3.5997
3.5829
3.3501
3.0039
Integral
1.0087
1.0316
0.9988
4.0000
2.9971
3.0039
(ppm)

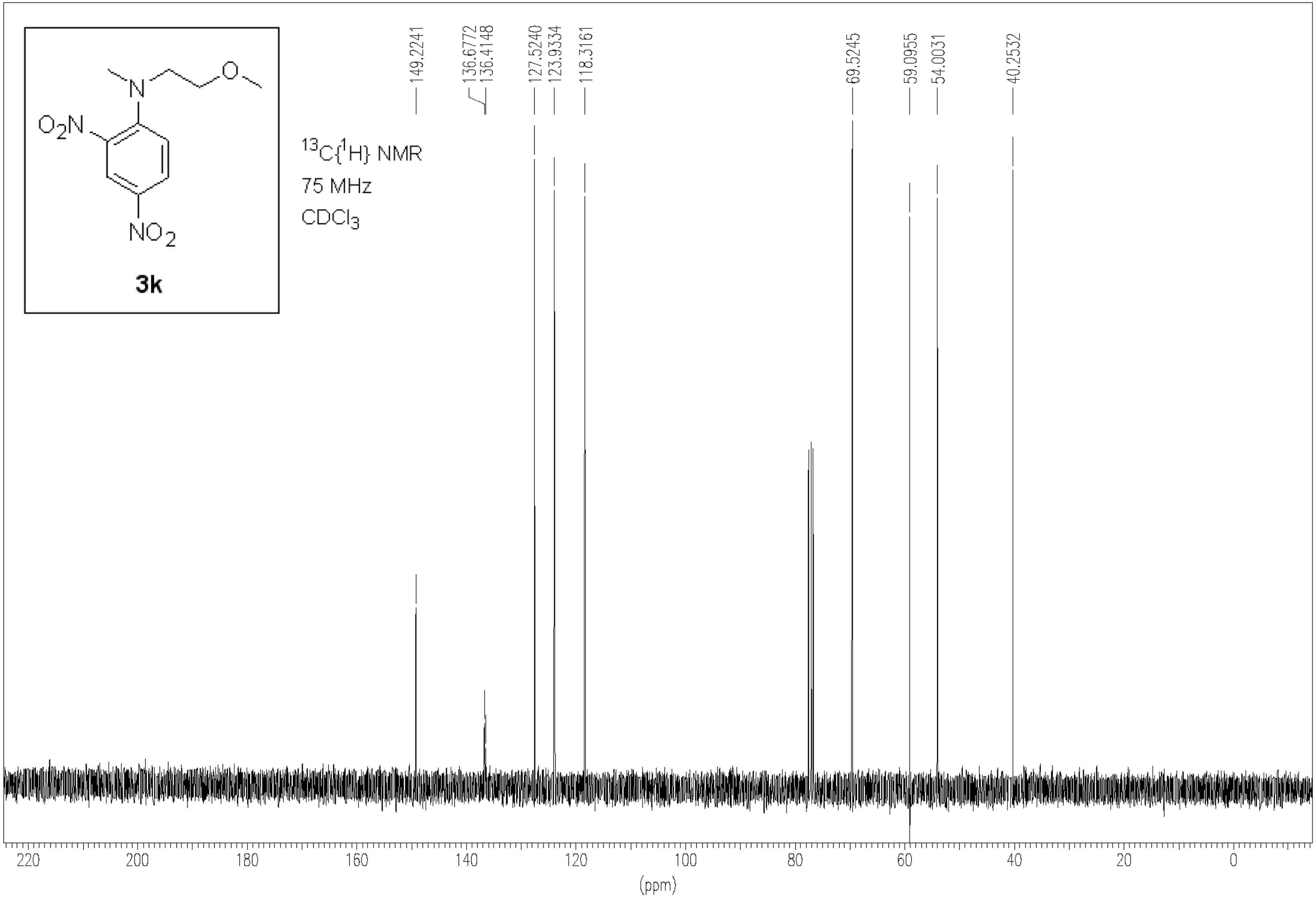

3k
$^{13}C\{^1H\}$ NMR
75 MHz
$CDCl_3$
149.2241
136.6772
136.4148
127.5240
123.9334
118.3161
69.5245
59.0955
54.0031
40.2532
(ppm)

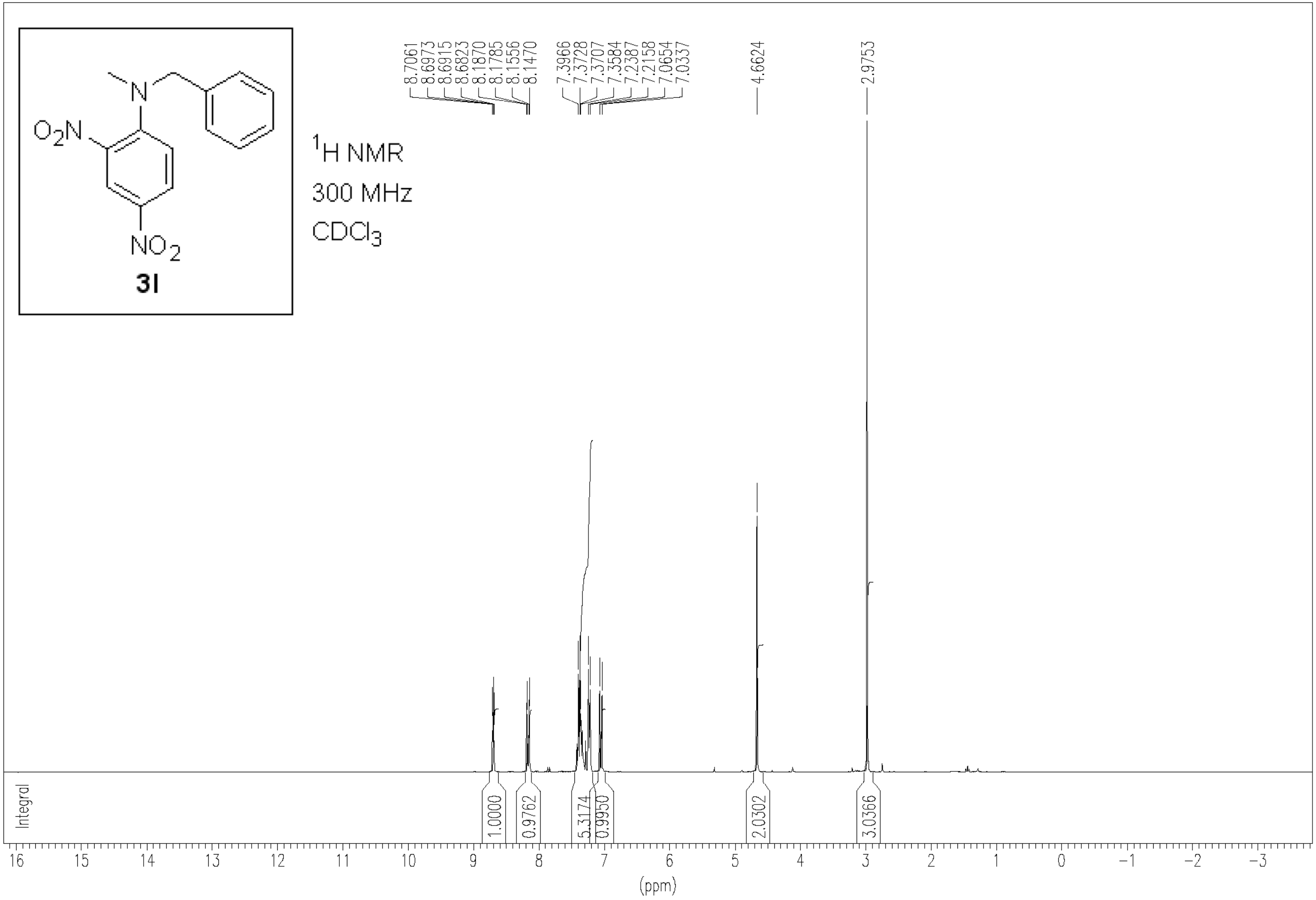

O_2N
N
NO_2
3l
^1H NMR
300 MHz
$CDCl_3$
8.7061
8.6973
8.6915
8.6823
8.1870
8.1785
8.1556
8.1470
7.3966
7.3728
7.3707
7.3584
7.2387
7.2158
7.0654
7.0337
4.6624
2.9753
Integral
1.0000
0.9762
5.3174
0.9950
2.0302
3.0366
16 15 14 13 12 11 10 9 8 7 6 5 4 3 2 1 0 −1 −2 −3
(ppm)

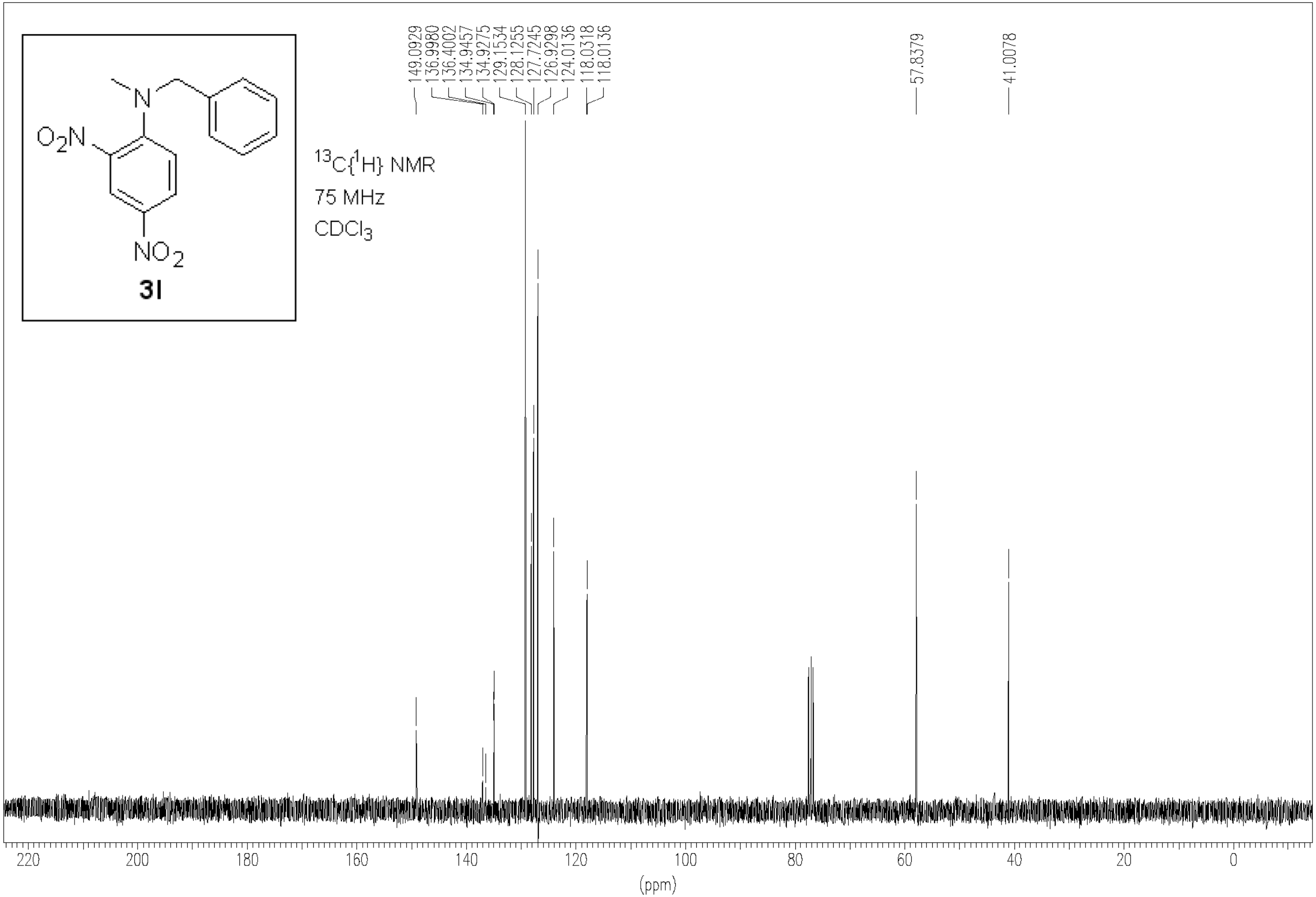

O2N
NO2
3l
13C{1H} NMR
75 MHz
CDCl3
149.0929
136.9980
136.4002
134.9457
134.9275
129.1534
128.1255
127.7245
126.9298
124.0136
118.0318
118.0136
57.8379
41.0078
220
200
180
160
140
120
100
80
60
40
20
0
(ppm)

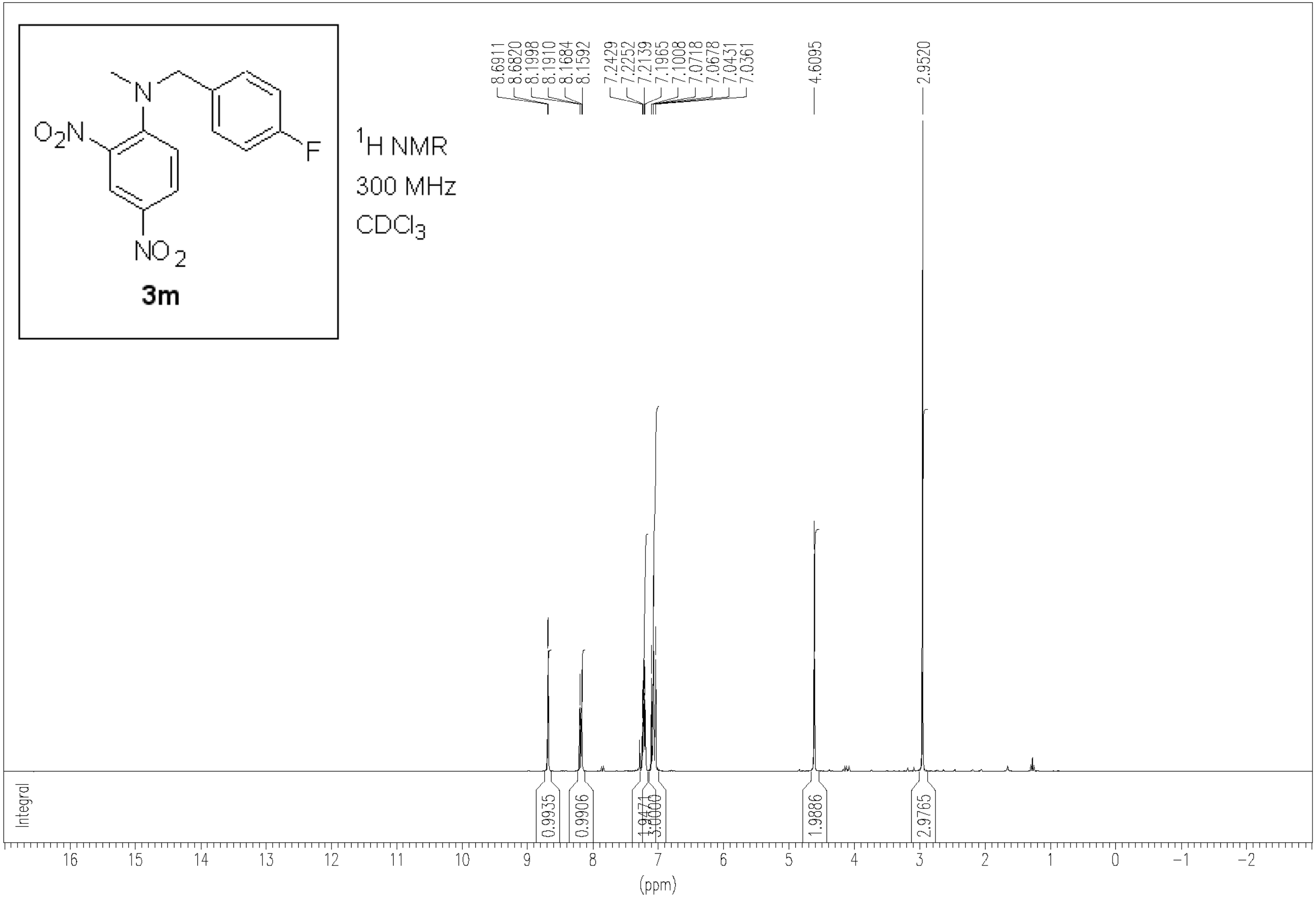
O2N
NO2
F
N
3m
1H NMR
300 MHz
CDCl3
8.6911
8.6820
8.1998
8.1910
8.1684
8.1592
7.2429
7.2252
7.2139
7.1965
7.1008
7.0718
7.0678
7.0431
7.0361
4.6095
2.9520
Integral
0.9935
0.9906
1.9471
3.0000
1.9886
2.9765
(ppm)

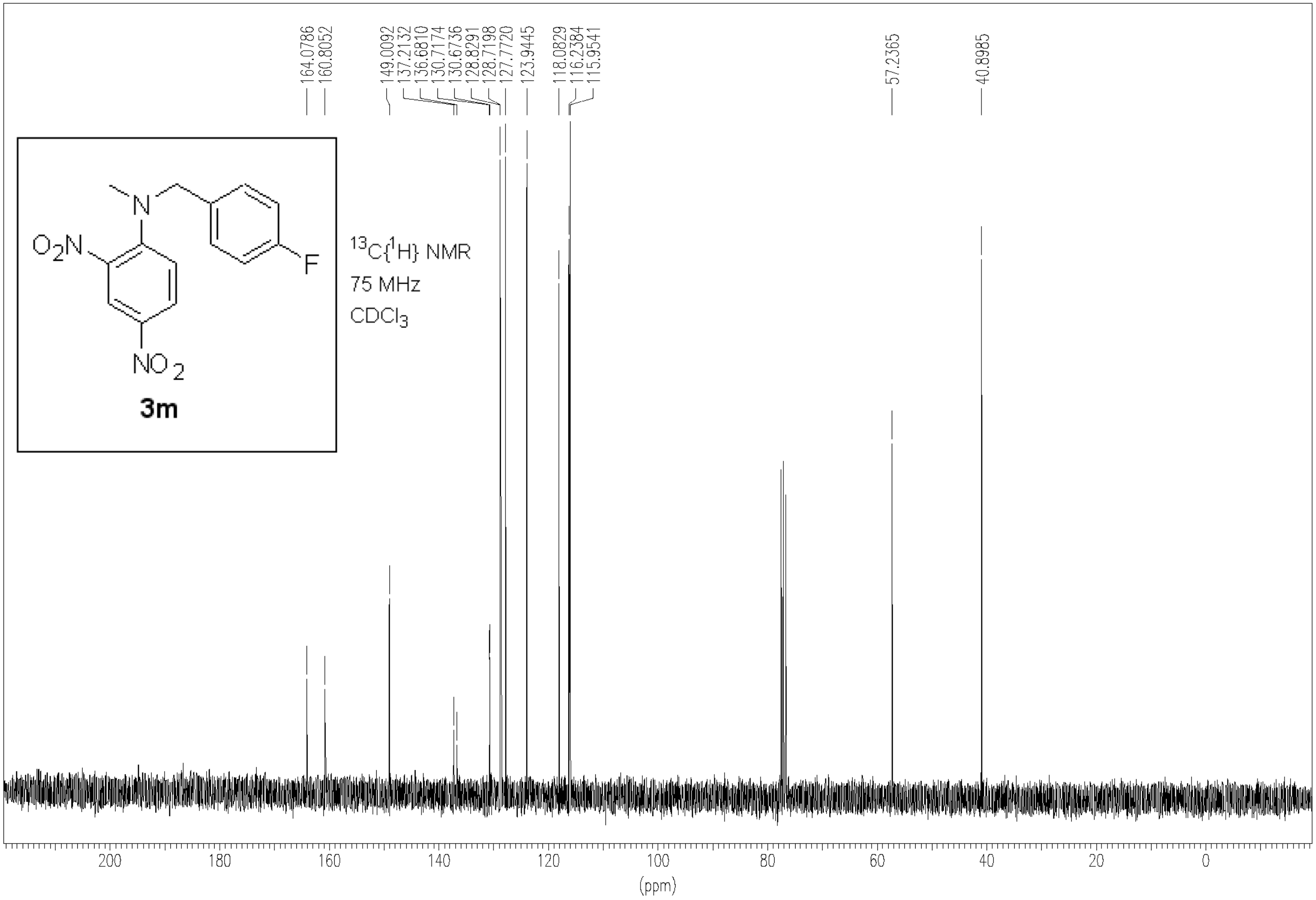
164.0786
160.8052
149.0092
137.2132
136.6810
130.7174
130.6736
128.8291
128.7198
127.7720
123.9445
118.0829
116.2384
115.9541
57.2365
40.8985
O2N
N
F
NO2
3m
13C{1H} NMR
75 MHz
CDCl3
200
180
160
140
120
100
80
60
40
20
0
(ppm)

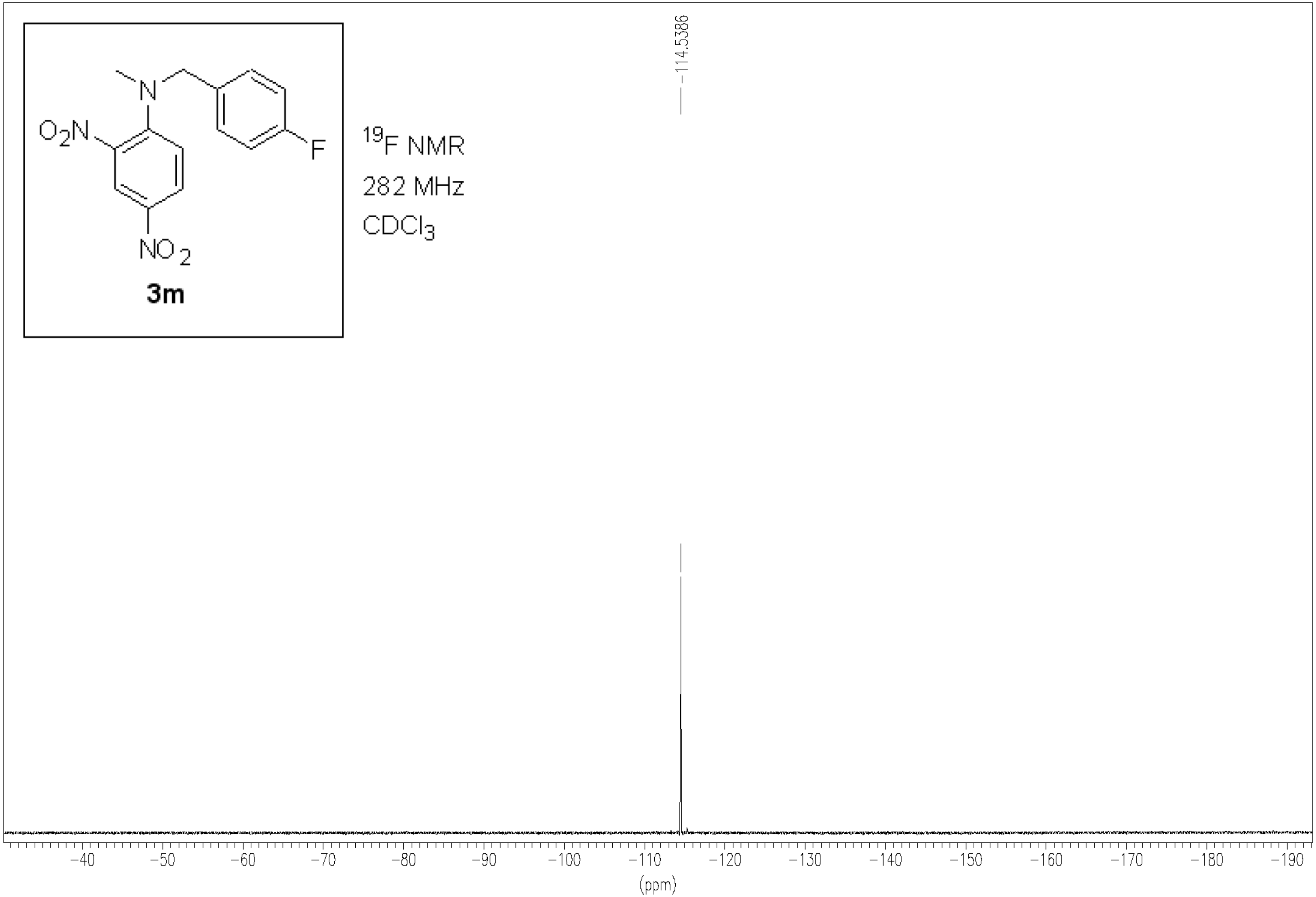

O2N
N
F
NO2
3m
19F NMR
282 MHz
CDCl3
-114.5386
-40
-50
-60
-70
-80
-90
-100
-110
-120
-130
-140
-150
-160
-170
-180
-190
(ppm)

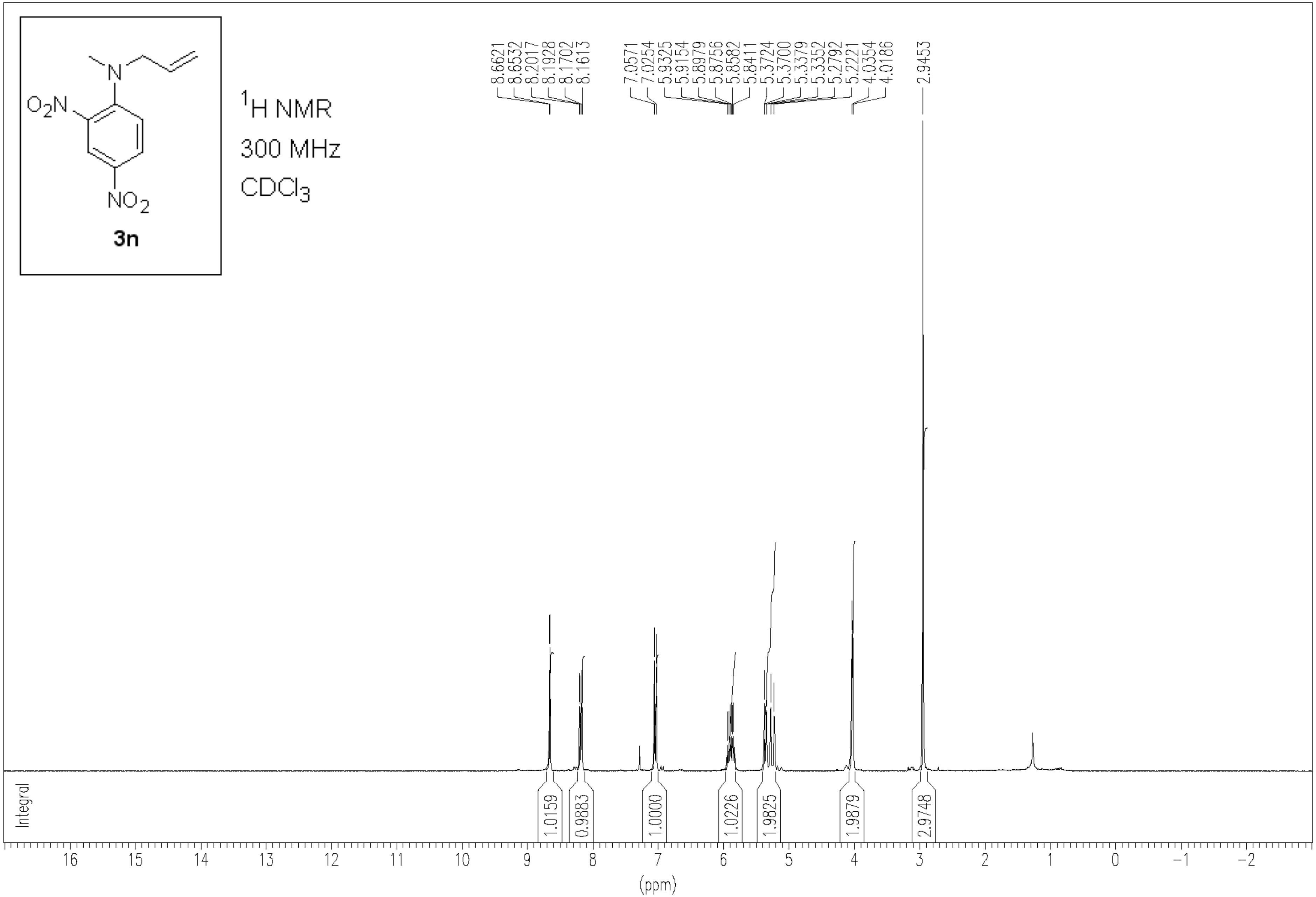

^{1}H NMR
300 MHz
$CDCl_3$
3n
8.6621
8.6532
8.2017
8.1928
8.1702
8.1613
7.0571
7.0254
5.9325
5.9154
5.8979
5.8756
5.8582
5.8411
5.3724
5.3700
5.3379
5.3352
5.2792
5.2221
4.0354
4.0186
2.9453
Integral
1.0159
0.9883
1.0000
1.0226
1.9825
1.9879
2.9748
(ppm)

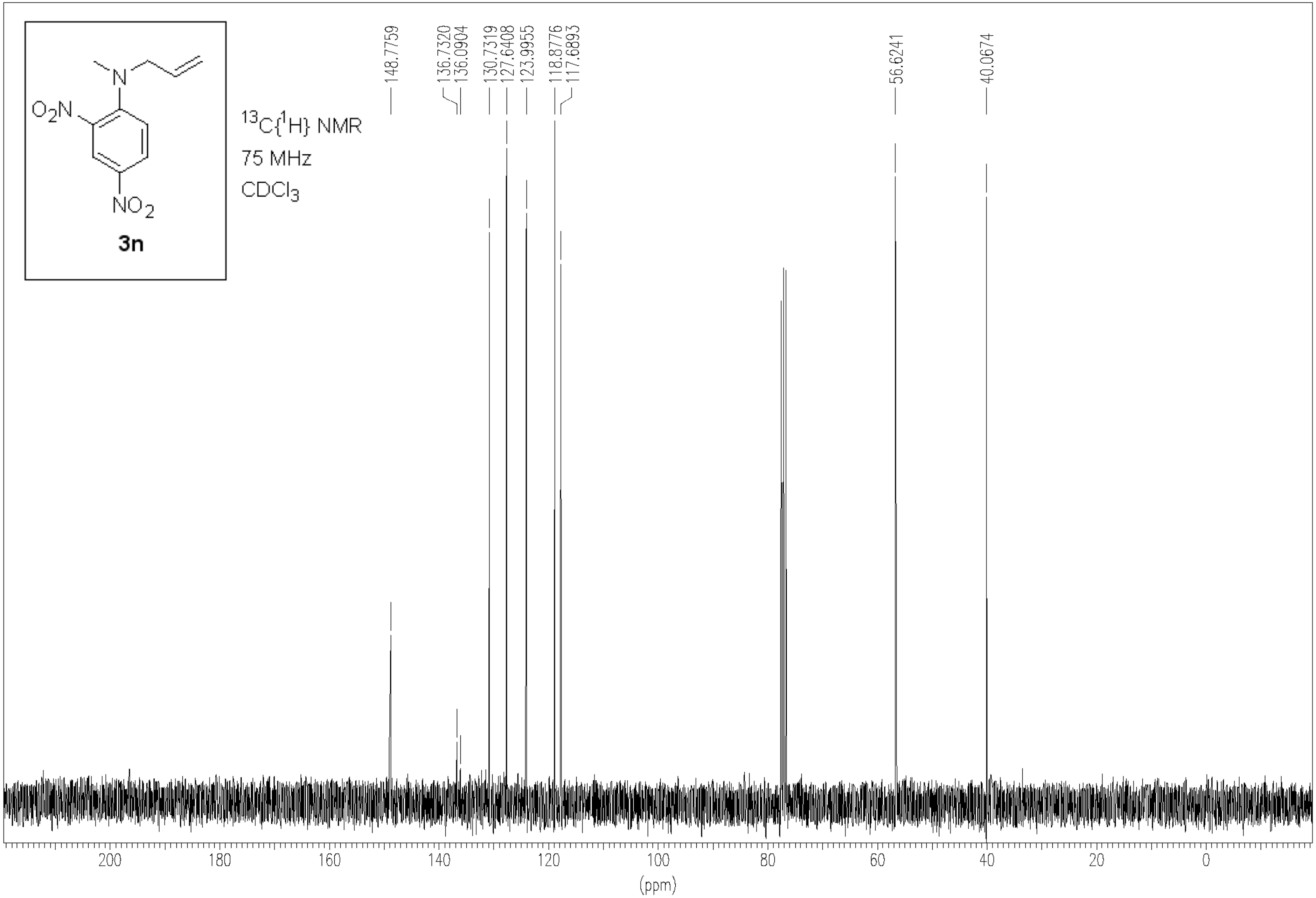

O_2N
N
NO_2
3n
$^{13}C\{^1H\}$ NMR
75 MHz
$CDCl_3$
148.7759
136.7320
136.0904
130.7319
127.6408
123.9955
118.8776
117.6893
56.6241
40.0674
200
180
160
140
120
100
80
60
40
20
0
(ppm)

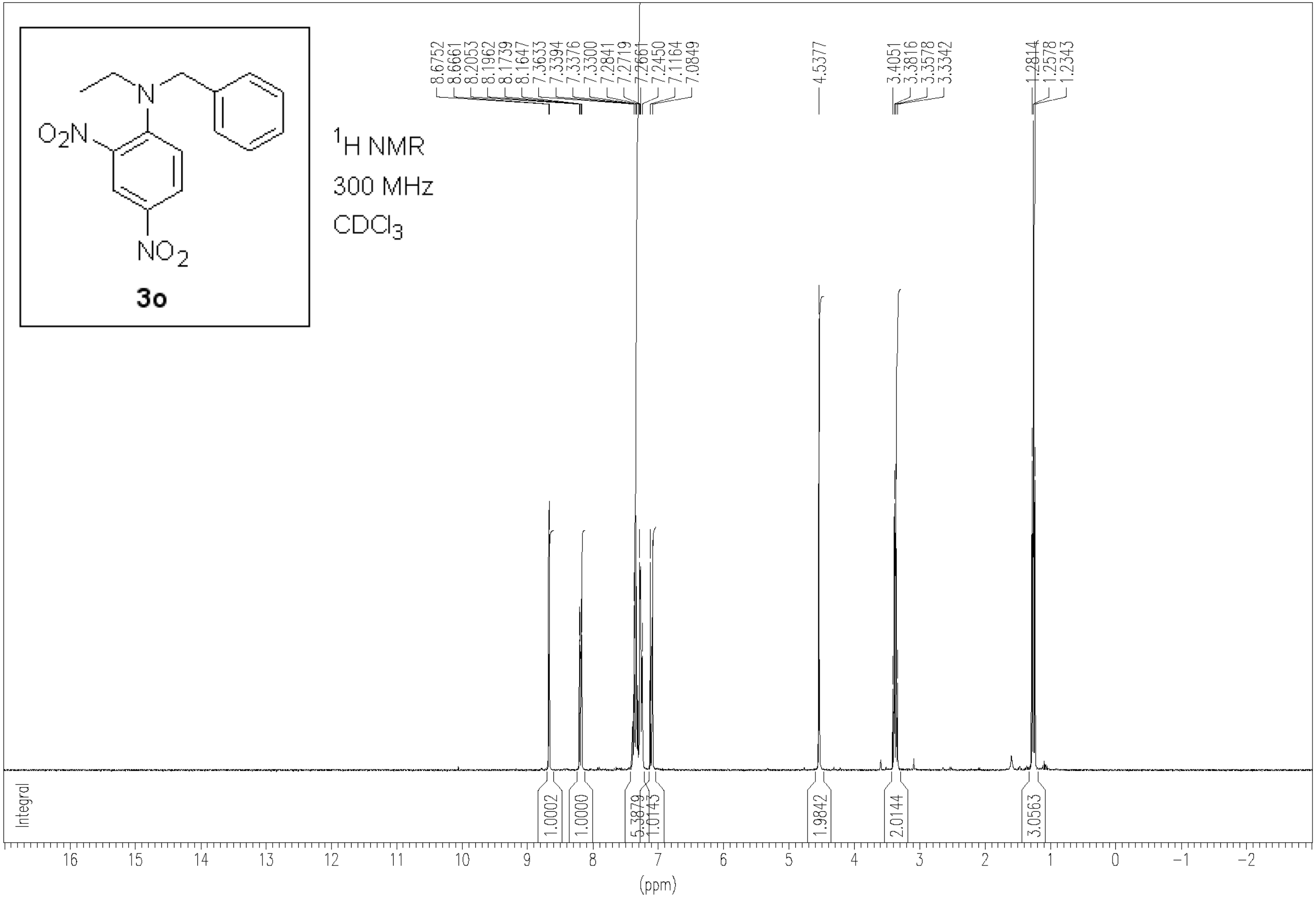

O2N
NO2
3o
1H NMR
300 MHz
CDCl3
8.6752
8.6661
8.2053
8.1962
8.1739
8.1647
7.3633
7.3394
7.3376
7.3300
7.2841
7.2719
7.2661
7.2450
7.1164
7.0849
4.5377
3.4051
3.3816
3.3578
3.3342
1.2814
1.2578
1.2343
Integral
1.0002
1.0000
5.3879
1.0143
1.9842
2.0144
3.0563
16 15 14 13 12 11 10 9 8 7 6 5 4 3 2 1 0 −1 −2
(ppm)

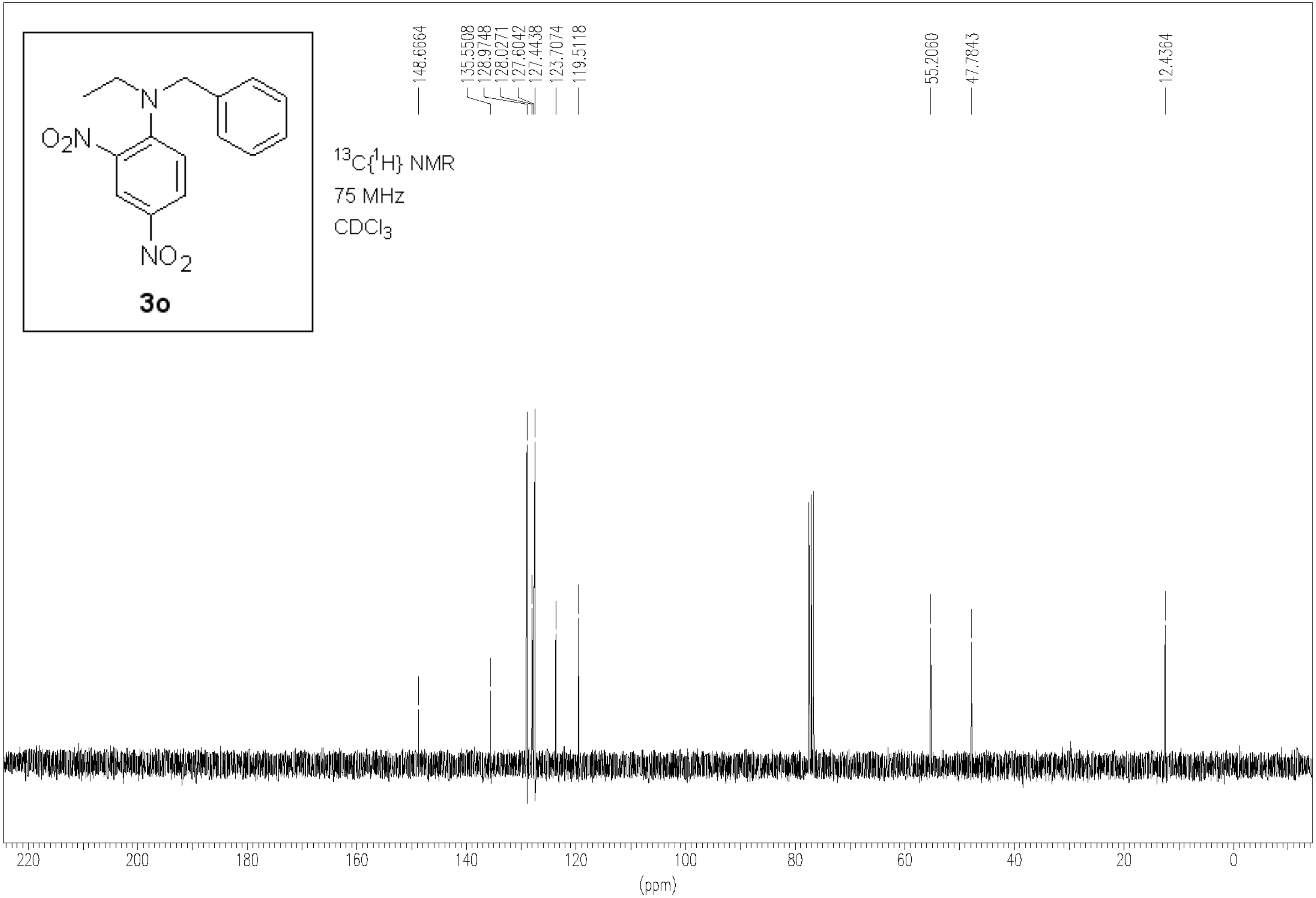

3o
13C{1H} NMR
75 MHz
CDCl3
148.6664
135.5508
128.9748
128.0271
127.6042
127.4438
123.7074
119.5118
55.2060
47.7843
12.4364
220
200
180
160
140
120
100
80
60
40
20
0
(ppm)

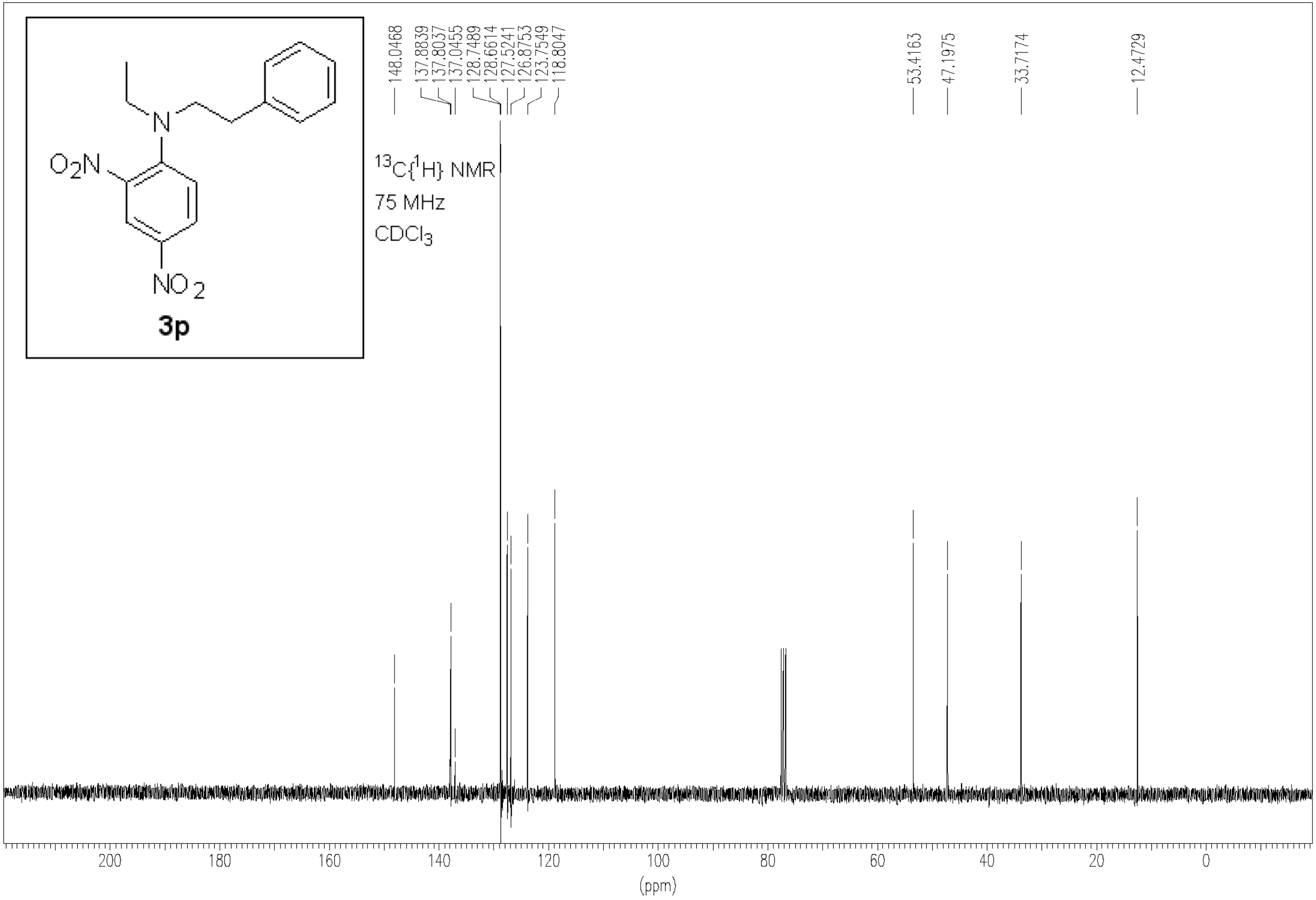
3p
13C{1H} NMR
75 MHz
CDCl3
148.0468
137.8839
137.8037
137.0455
128.7489
128.6614
127.5241
126.8753
123.7549
118.8047
53.4163
47.1975
33.7174
12.4729
(ppm)

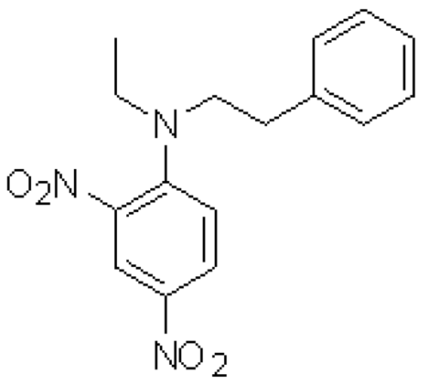

3p

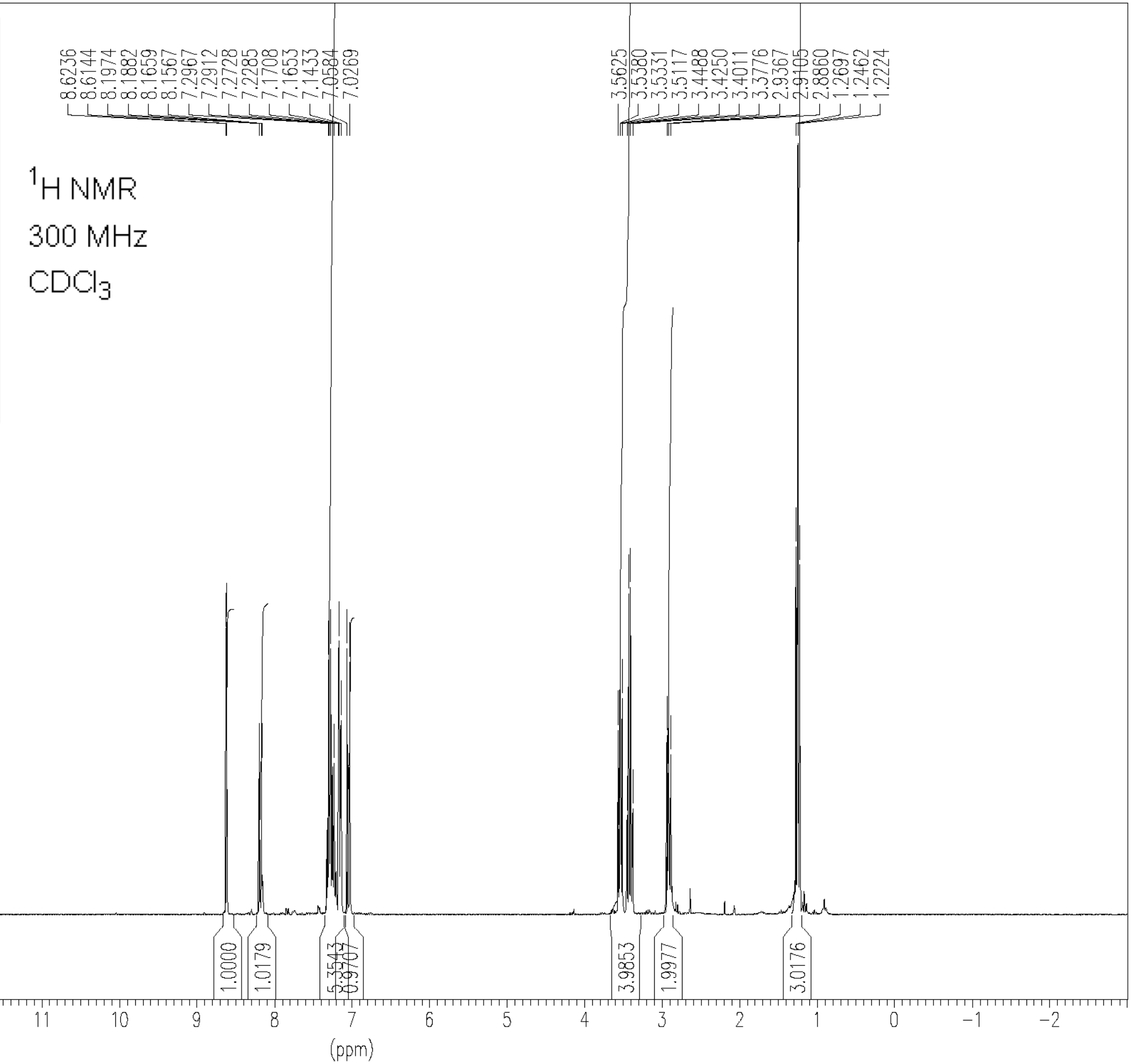

8.6236
8.6144
8.1974
8.1882
8.1659
8.1567
7.2967
7.2912
7.2728
7.2285
7.1708
7.1653
7.1433
7.0584
7.0269
3.5625
3.5380
3.5331
3.5117
3.4488
3.4250
3.4011
3.3776
2.9367
2.9105
2.8860
1.2697
1.2462
1.2224
1H NMR
300 MHz
CDCl3
Integral
1.0000
1.0179
5.3543
0.9707
3.9853
1.9977
3.0176
16
15
14
13
12
11
10
9
8
7
6
5
4
3
2
1
0
−1
−2
(ppm)

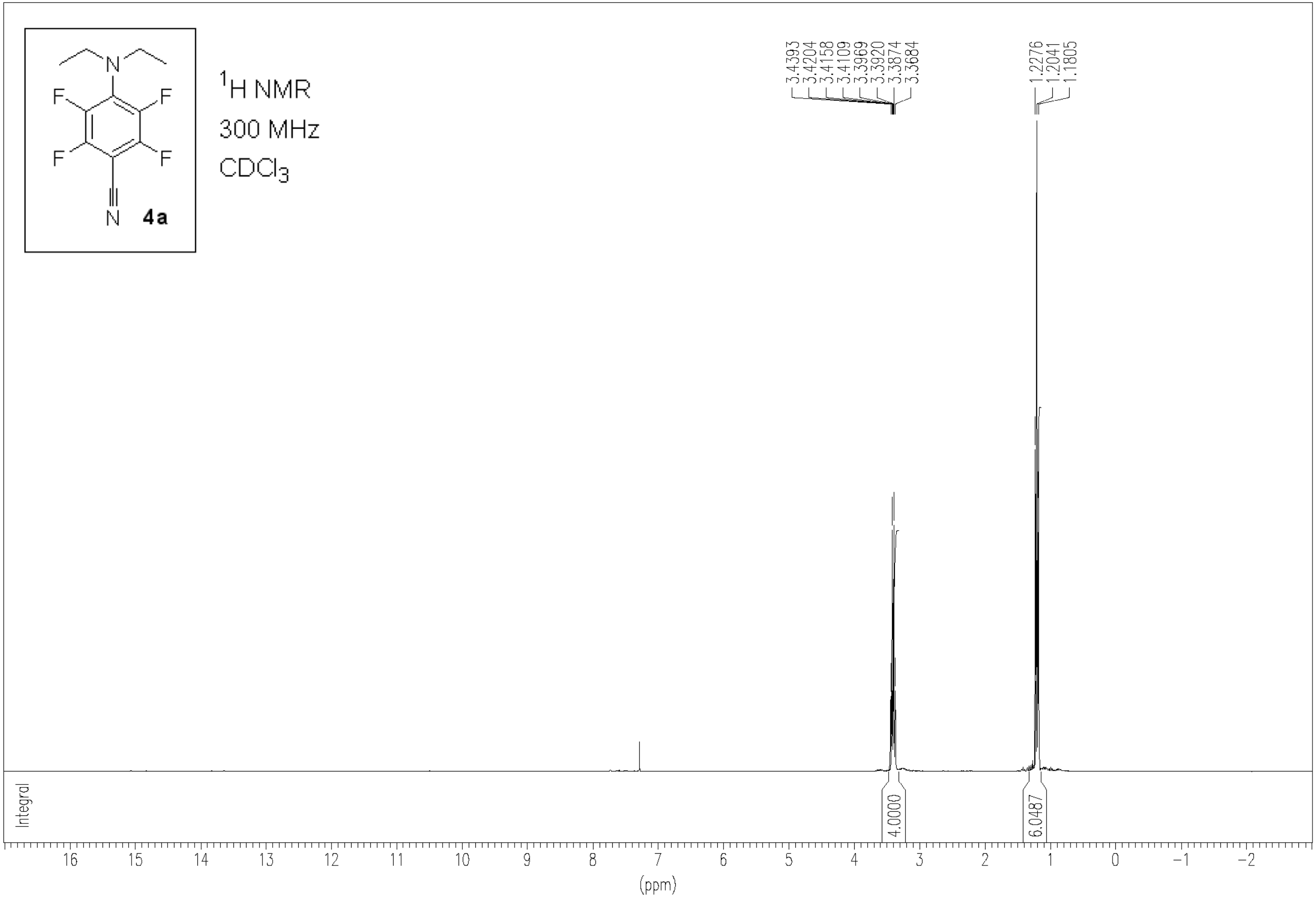

4a
1H NMR
300 MHz
CDCl3
3.4393
3.4204
3.4158
3.4109
3.3969
3.3920
3.3874
3.3684
1.2276
1.2041
1.1805
Integral
4.0000
6.0487
(ppm)

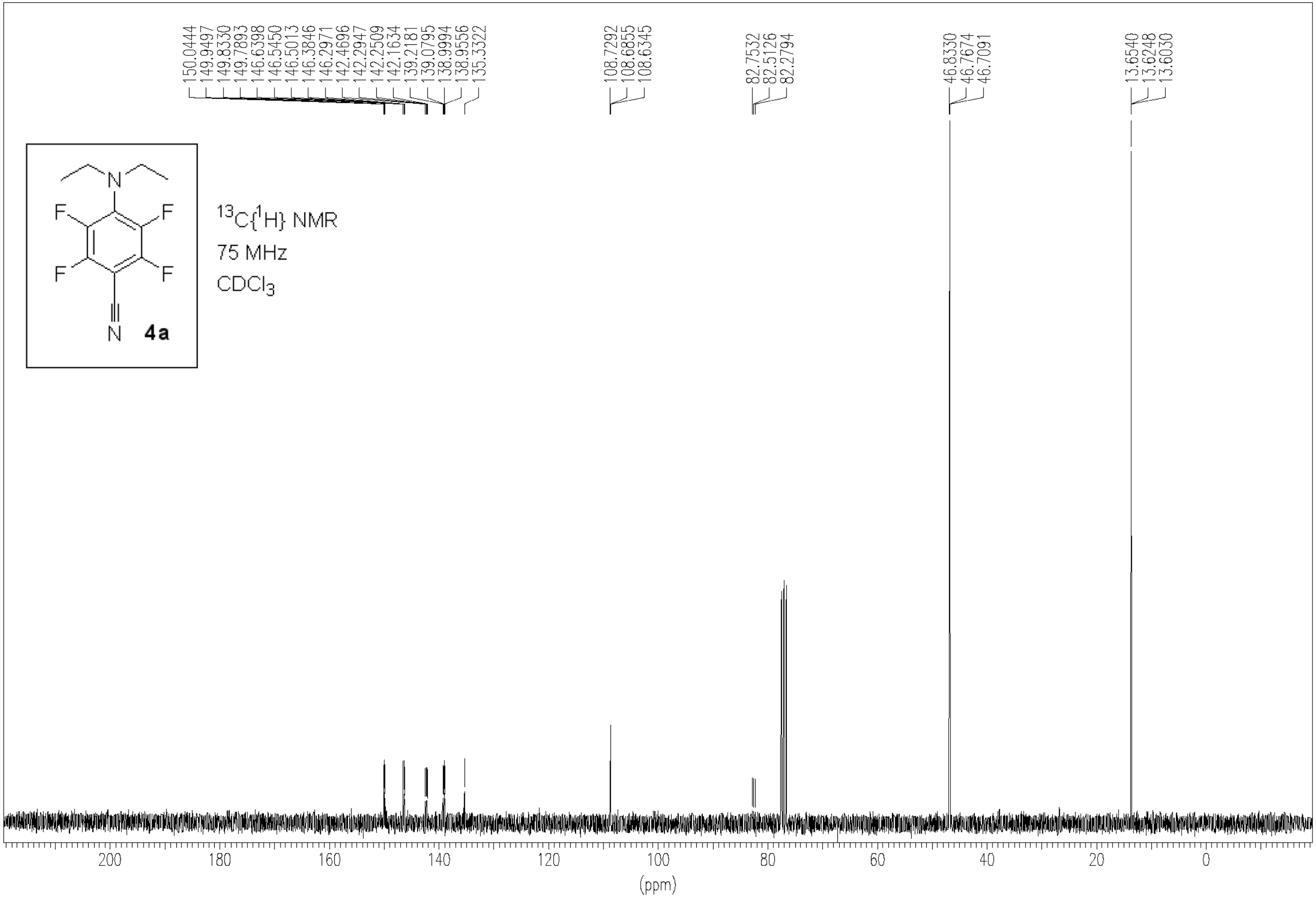
150.0444
149.9497
149.8330
149.7893
146.6398
146.5450
146.5013
146.3846
146.2971
142.4696
142.2947
142.2509
142.1634
139.2181
139.0795
138.9994
138.9556
135.3322
108.7292
108.6855
108.6345
82.7532
82.5126
82.2794
46.8330
46.7674
46.7091
13.6540
13.6248
13.6030
F
F
F
F
N
N
4a
$^{13}C\{^1H\}$ NMR
75 MHz
$CDCl_3$
200
180
160
140
120
100
80
60
40
20
0
(ppm)

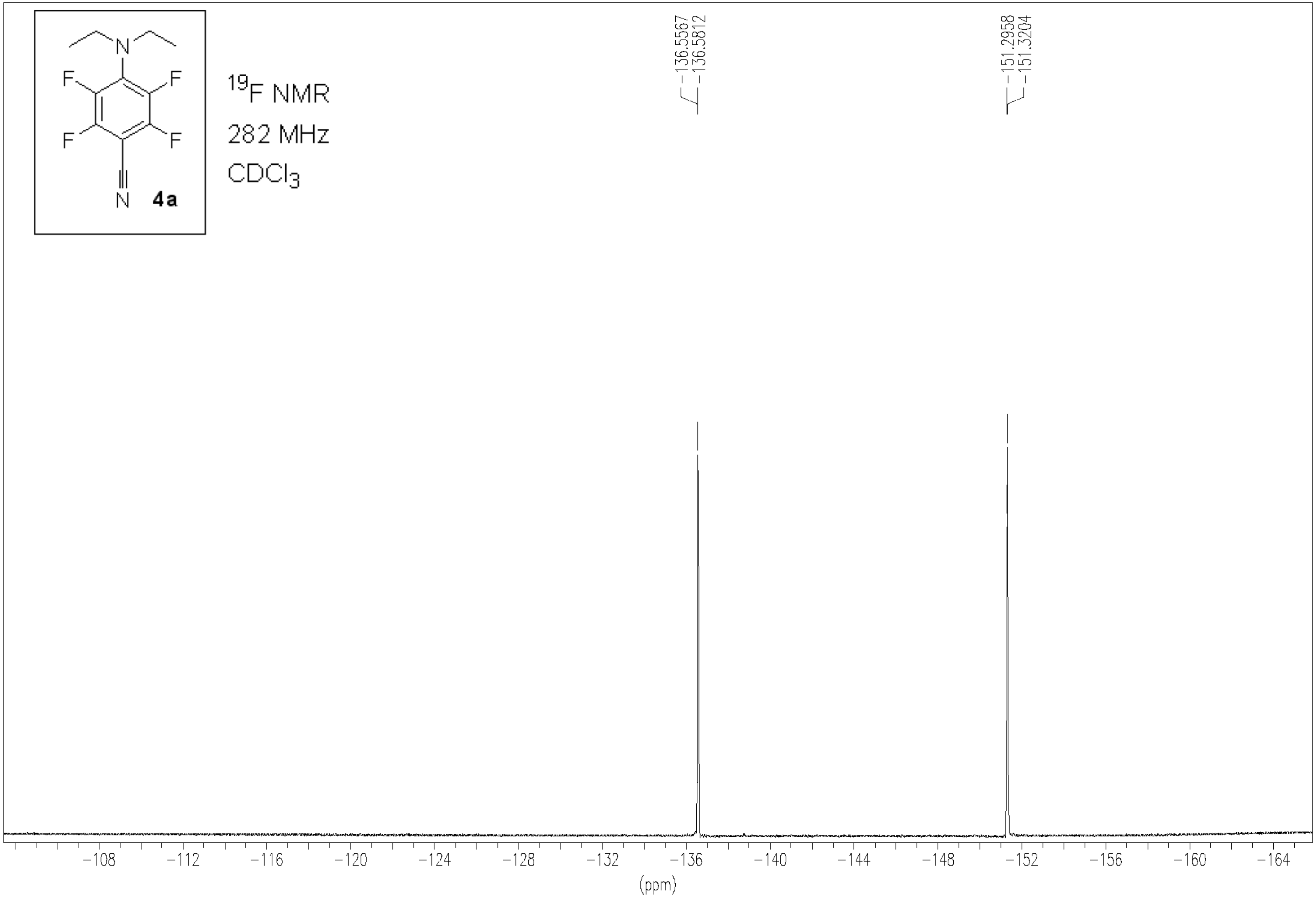

4a
19F NMR
282 MHz
CDCl3
-136.5567
-136.5812
-151.2958
-151.3204
-108
-112
-116
-120
-124
-128
-132
-136
-140
-144
-148
-152
-156
-160
-164
(ppm)

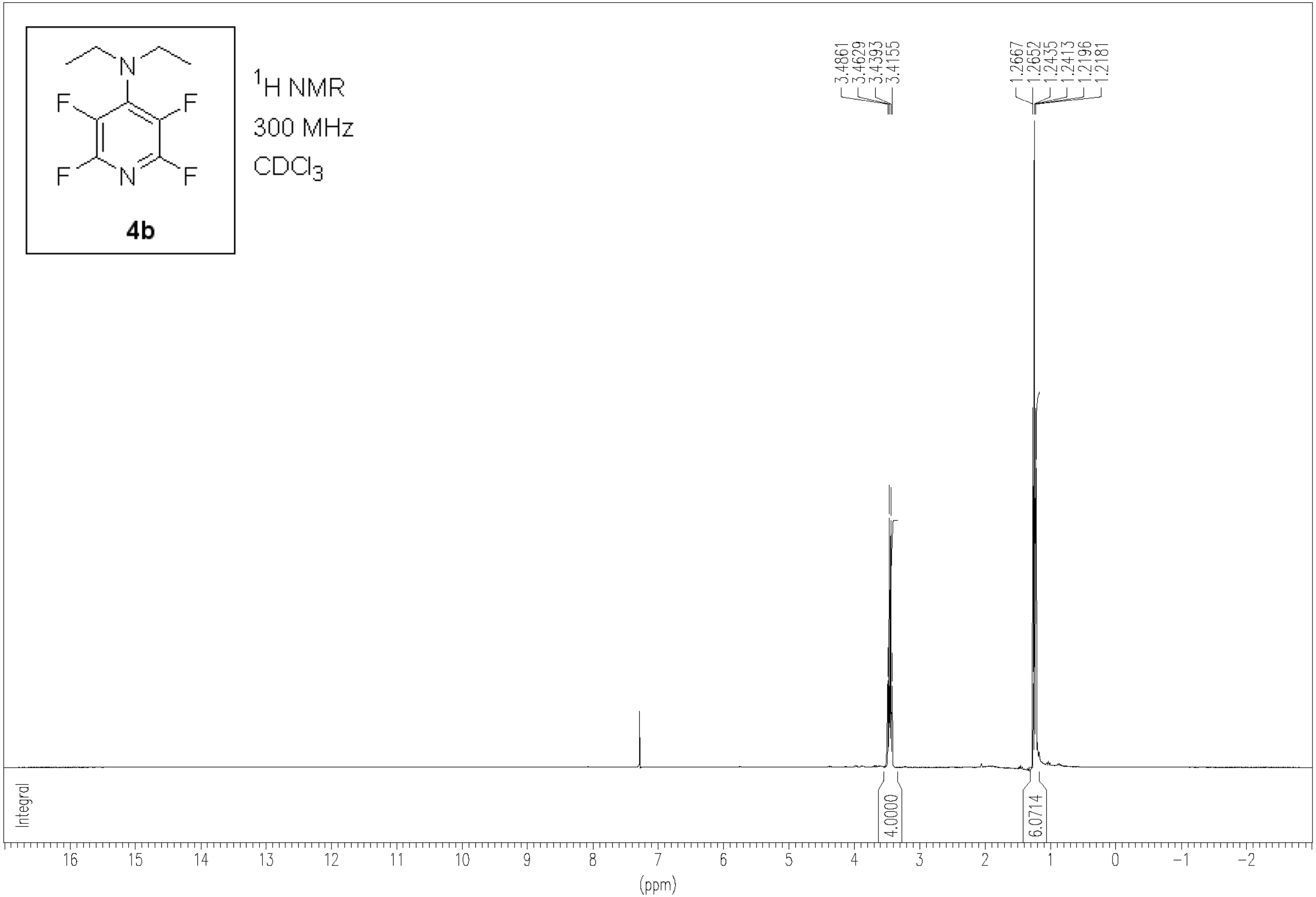
4b
1H NMR
300 MHz
CDCl3
3.4861
3.4629
3.4393
3.4155
1.2667
1.2652
1.2435
1.2413
1.2196
1.2181
Integral
4.0000
6.0714
(ppm)

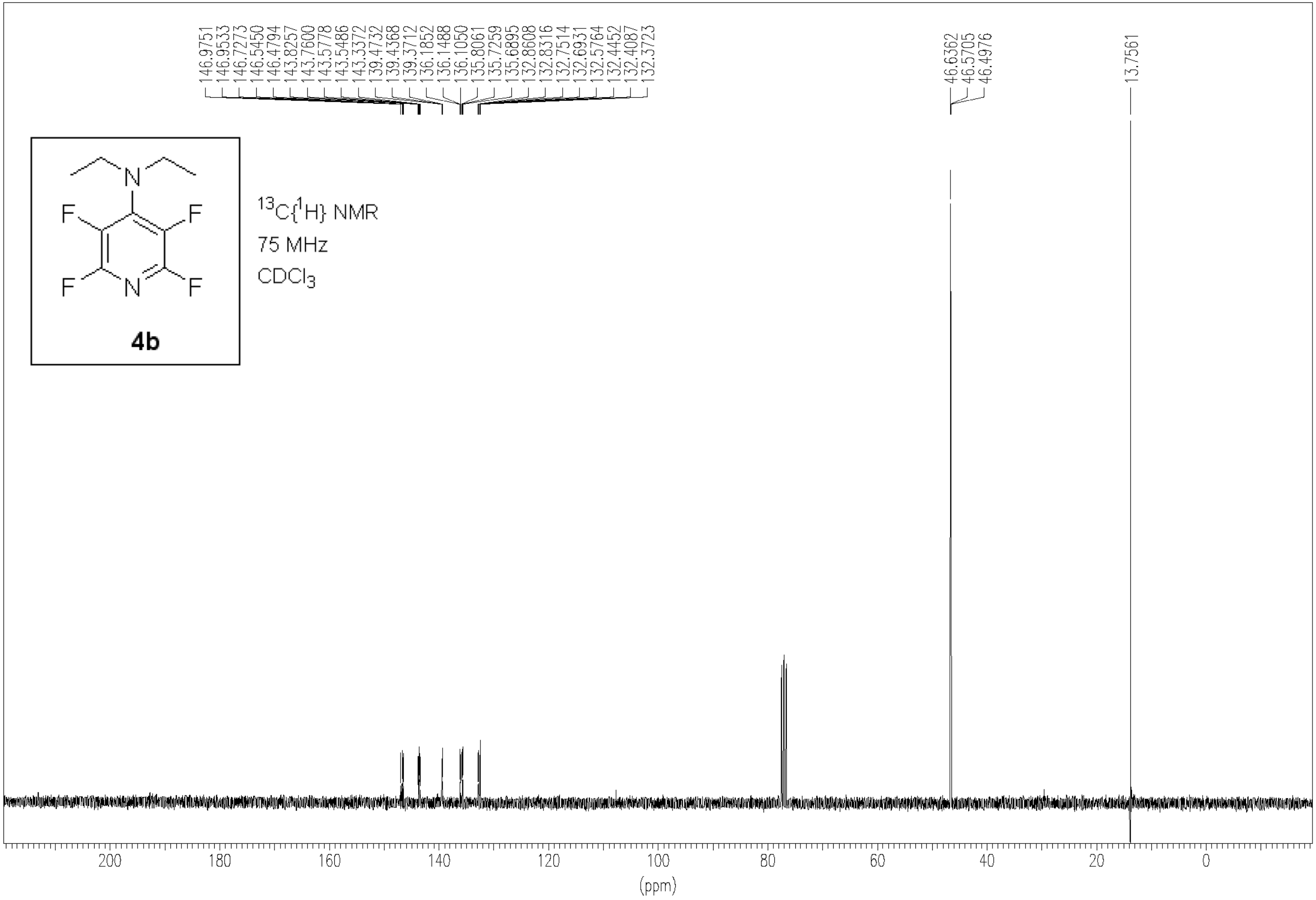
146.9751
146.9533
146.7273
146.5450
146.4794
143.8257
143.7600
143.5778
143.5486
143.3372
139.4732
139.4368
139.3712
136.1852
136.1488
136.1050
135.8061
135.7259
135.6895
132.8608
132.8316
132.7514
132.6931
132.5764
132.4452
132.4087
132.3723
46.6362
46.5705
46.4976
13.7561
N
F
F
F
F
N
4b
$^{13}C\{^{1}H\}$ NMR
75 MHz
$CDCl_3$
200
180
160
140
120
100
80
60
40
20
0
(ppm)

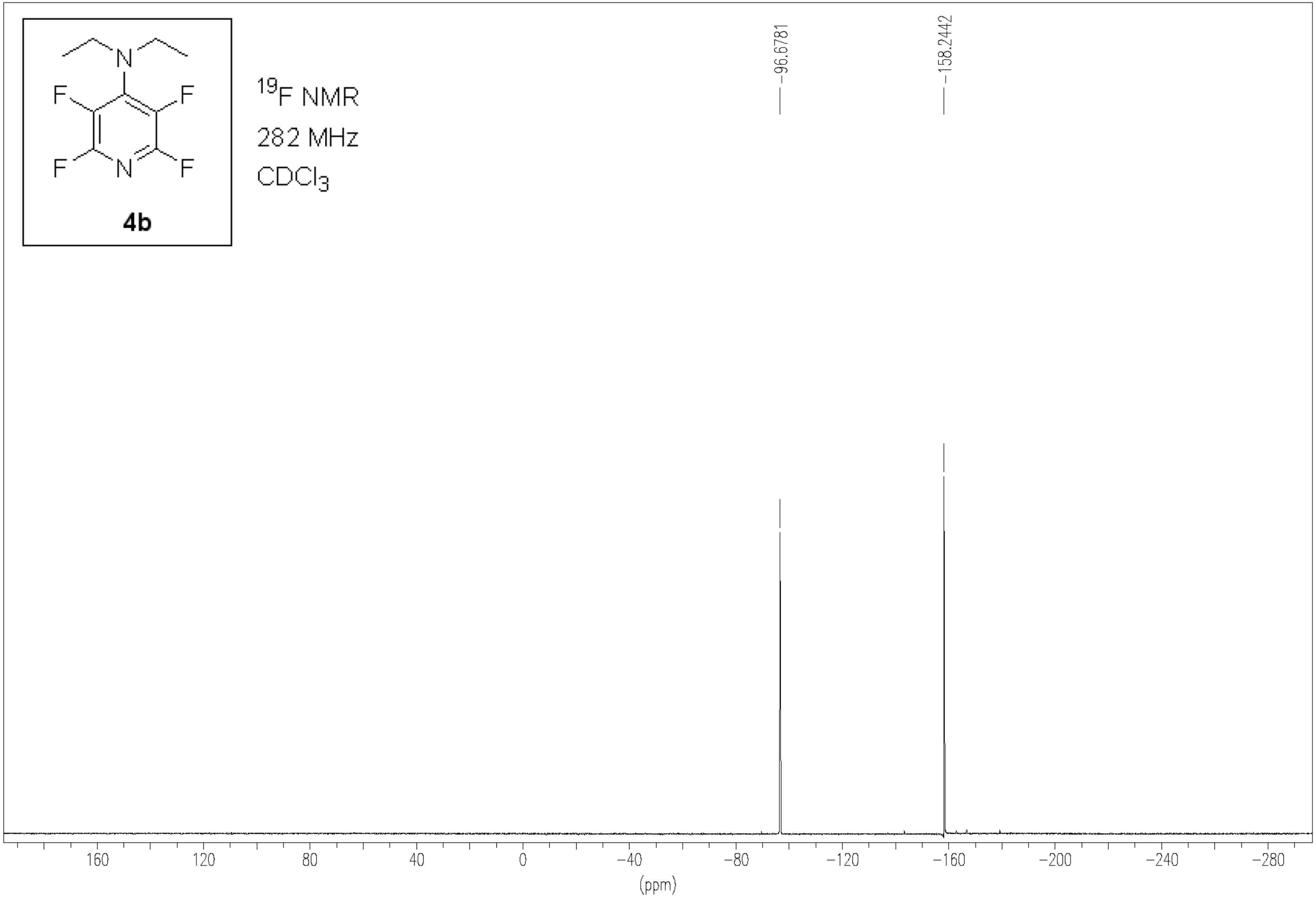

4b
19F NMR
282 MHz
CDCl3
-96.6781
-158.2442
160
120
80
40
0
-40
-80
-120
-160
-200
-240
-280
(ppm)

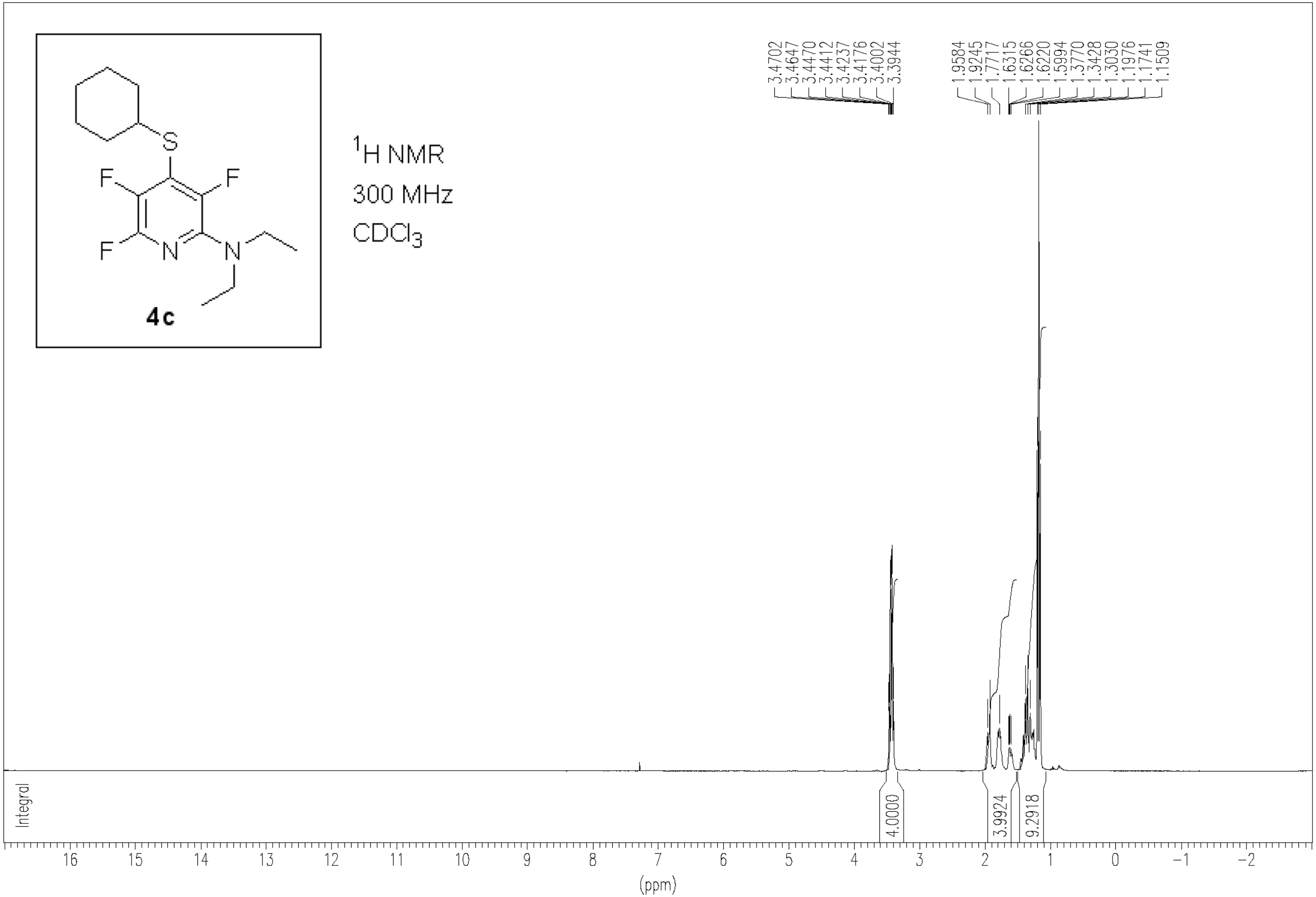

4c
1H NMR
300 MHz
CDCl3
3.4702
3.4647
3.4470
3.4412
3.4237
3.4176
3.4002
3.3944
1.9584
1.9245
1.7717
1.6315
1.6266
1.6220
1.5994
1.3770
1.3428
1.3030
1.1976
1.1741
1.1509
Integral
4.0000
3.9924
9.2918
16
15
14
13
12
11
10
9
8
7
6
5
4
3
2
1
0
−1
−2
(ppm)

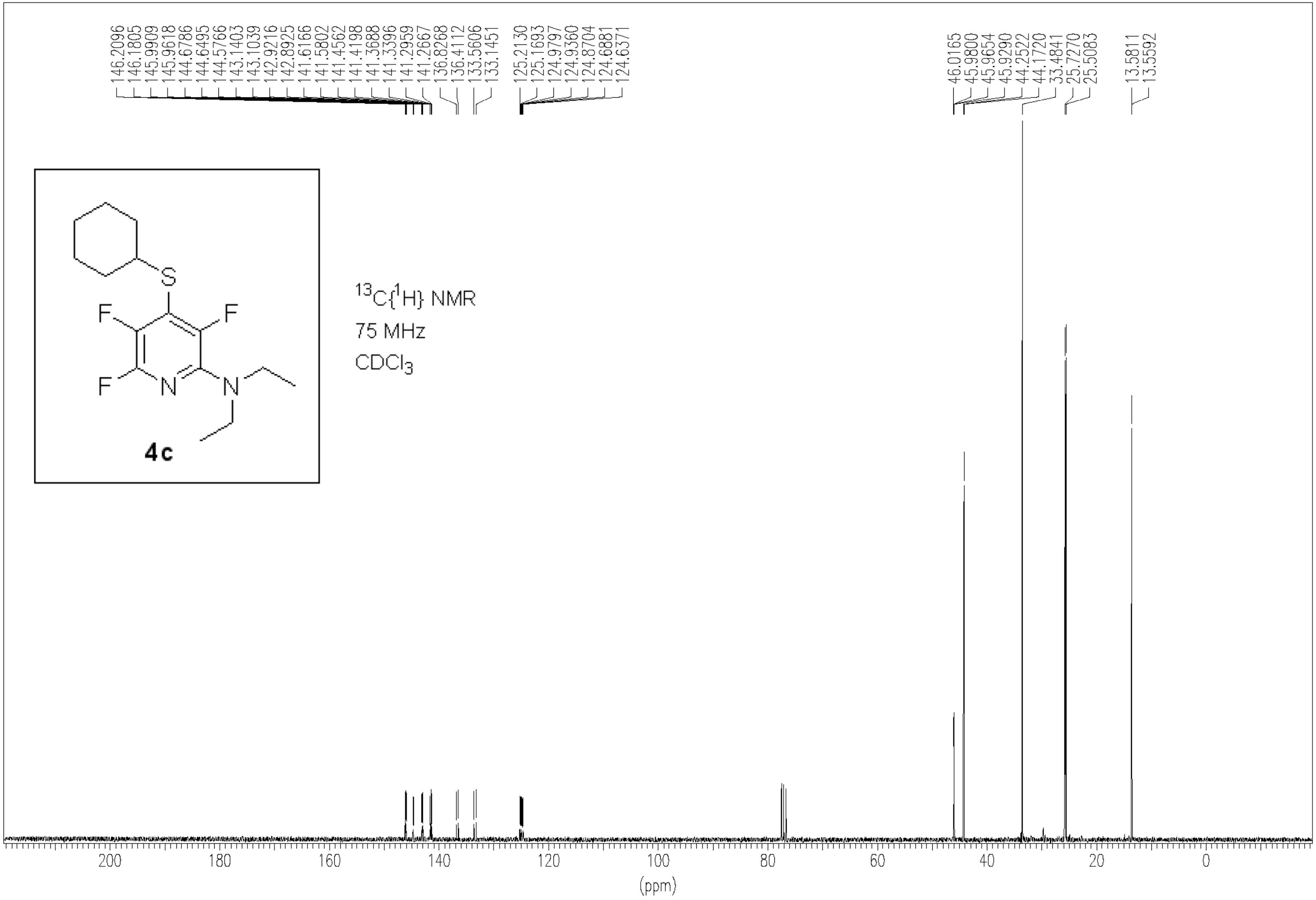
146.2096
146.1805
145.9909
145.9618
144.6786
144.6495
144.5766
143.1403
143.1039
142.9216
142.8925
141.6166
141.5802
141.4562
141.4198
141.3688
141.3396
141.2959
141.2667
136.8268
136.4112
133.5606
133.1451
125.2130
125.1693
124.9797
124.9360
124.8704
124.6881
124.6371
46.0165
45.9800
45.9654
45.9290
44.2522
44.1720
33.4841
25.7270
25.5083
13.5811
13.5592
S
F
F
F
N
N
4c
$^{13}C\{^{1}H\}$ NMR
75 MHz
$CDCl_3$
200
180
160
140
120
100
80
60
40
20
0
(ppm)

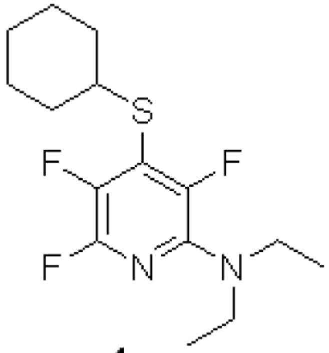

4c

^{19}F NMR
282 MHz
$CDCl_3$

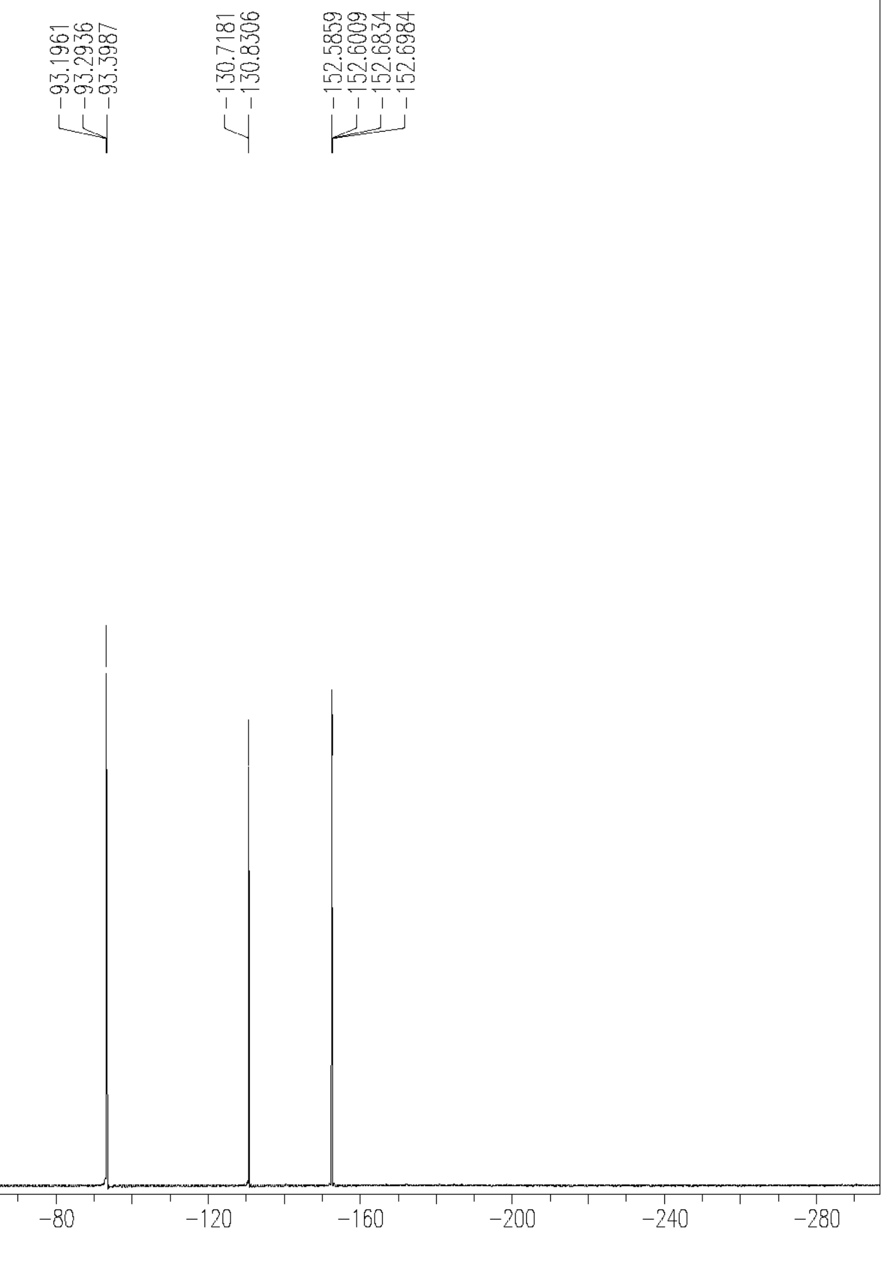

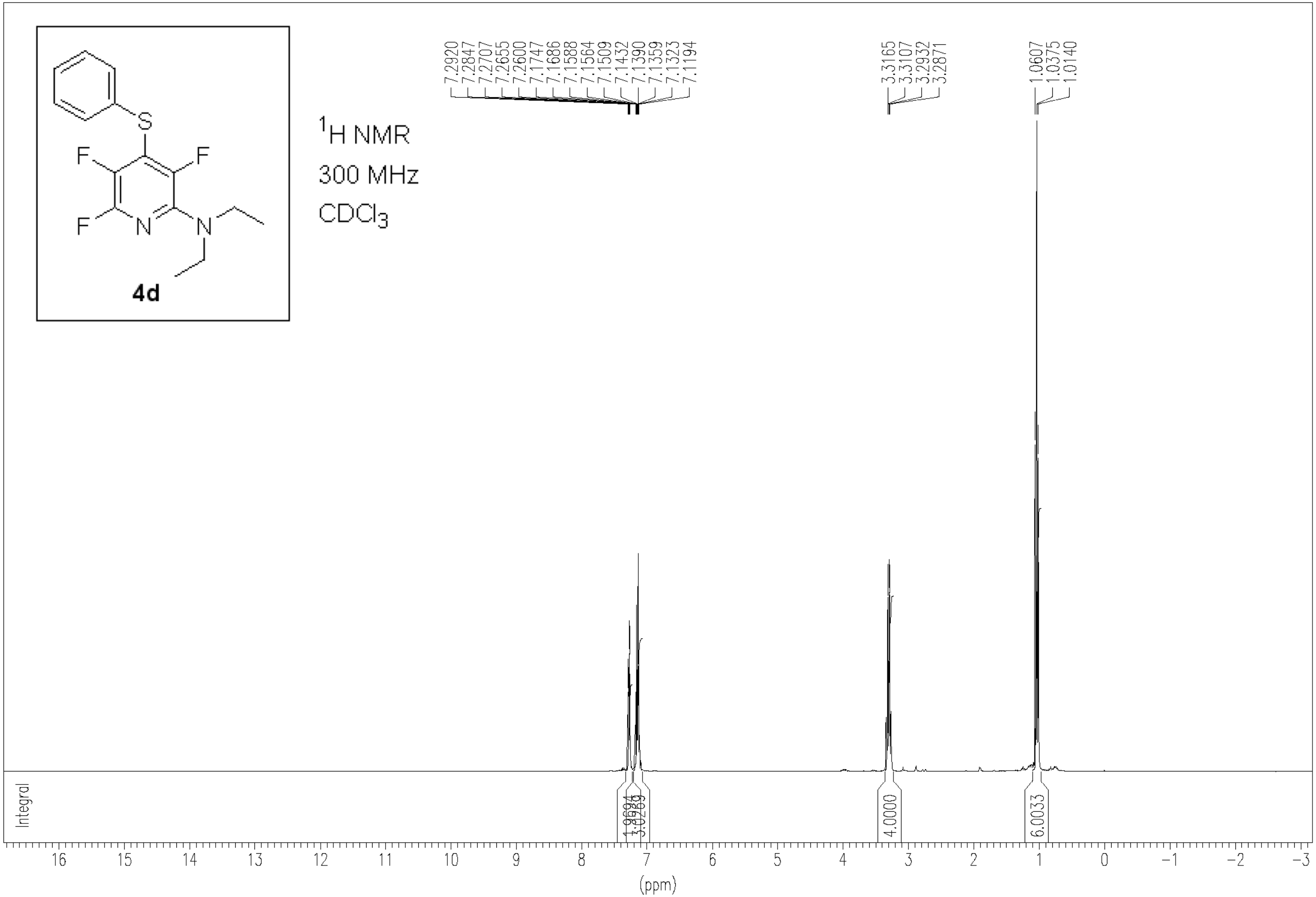

4d
^{1}H NMR
300 MHz
$CDCl_3$
7.2920
7.2847
7.2707
7.2655
7.2600
7.1747
7.1686
7.1588
7.1564
7.1509
7.1432
7.1390
7.1359
7.1323
7.1194
3.3165
3.3107
3.2932
3.2871
1.0607
1.0375
1.0140
Integral
4.0000
6.0033
(ppm)

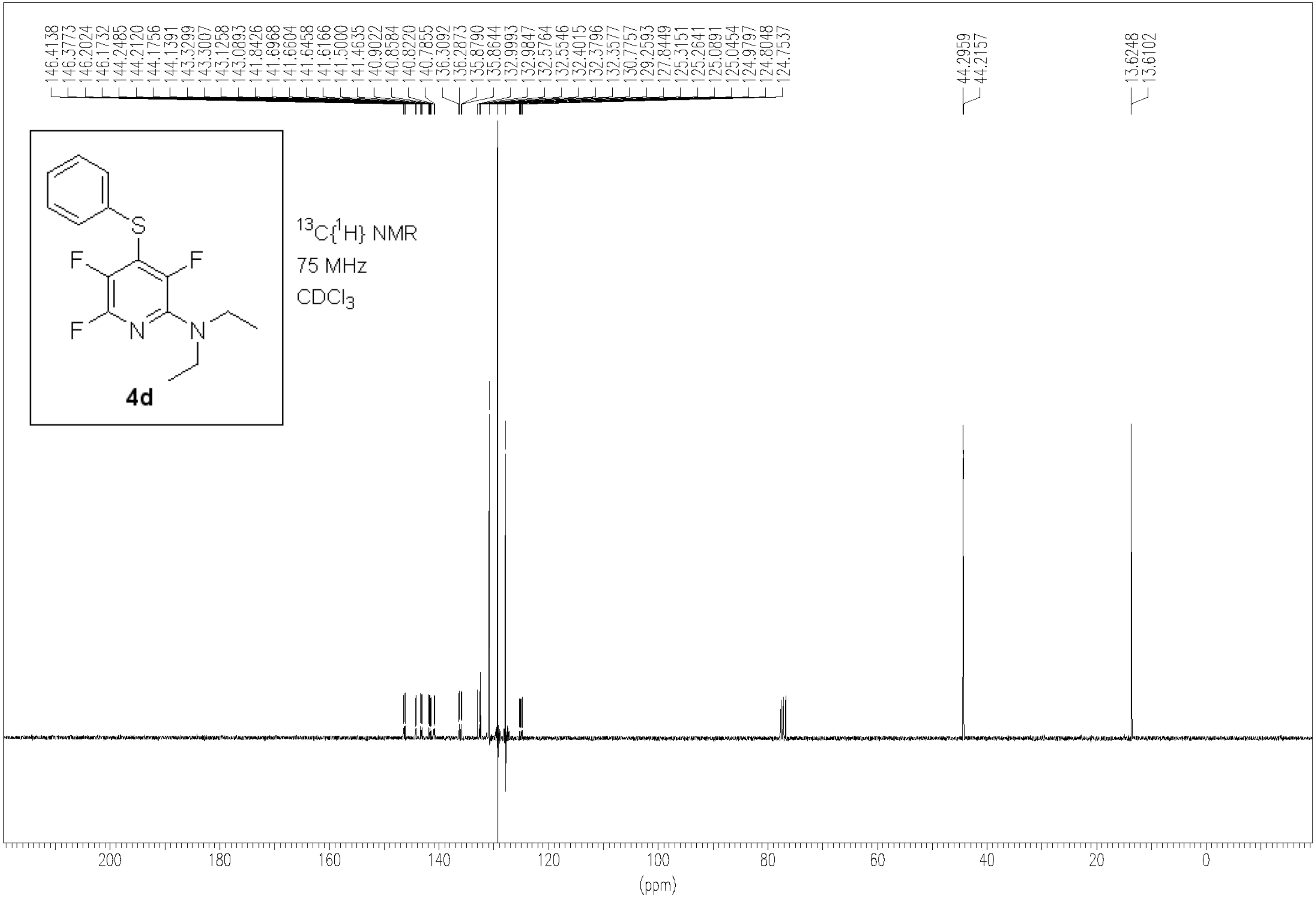
146.4138
146.3773
146.2024
146.1732
144.2485
144.2120
144.1756
144.1391
143.3299
143.3007
143.1258
143.0893
141.8426
141.6968
141.6604
141.6458
141.6166
141.5000
141.4635
140.9022
140.8584
140.8220
140.7855
136.3092
136.2873
135.8790
135.8644
132.9993
132.9847
132.5764
132.5546
132.4015
132.3796
132.3577
130.7757
129.2593
127.8449
125.3151
125.2641
125.0891
125.0454
124.9797
124.8048
124.7537
44.2959
44.2157
13.6248
13.6102
S
F
F
F
F
N
N
4d
$^{13}C\{^1H\}$ NMR
75 MHz
$CDCl_3$
200
180
160
140
120
100
80
60
40
20
0
(ppm)

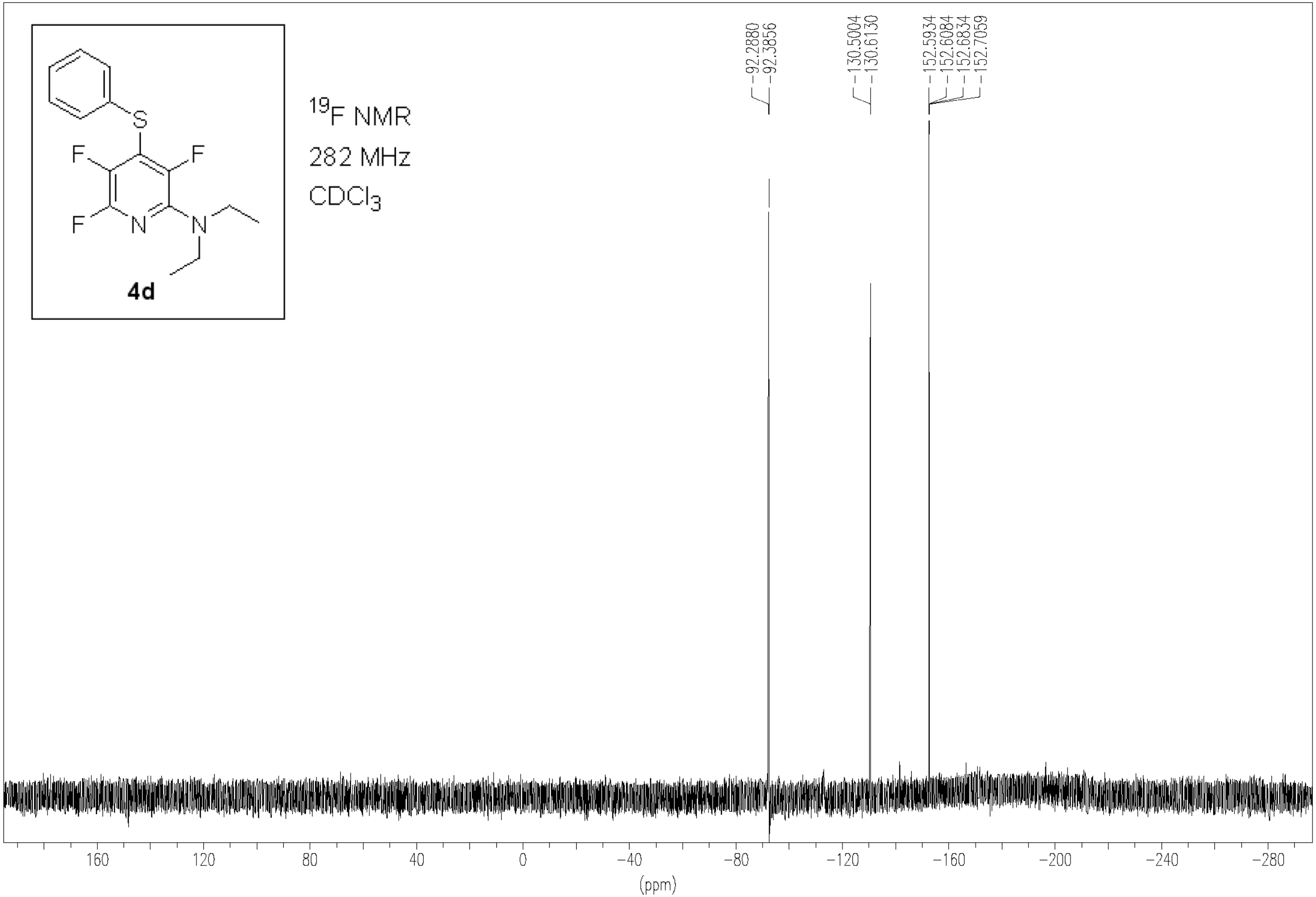

4d
^{19}F NMR
282 MHz
$CDCl_3$
−92.2880
−92.3856
−130.5004
−130.6130
−152.5934
−152.6084
−152.6834
−152.7059
(ppm)

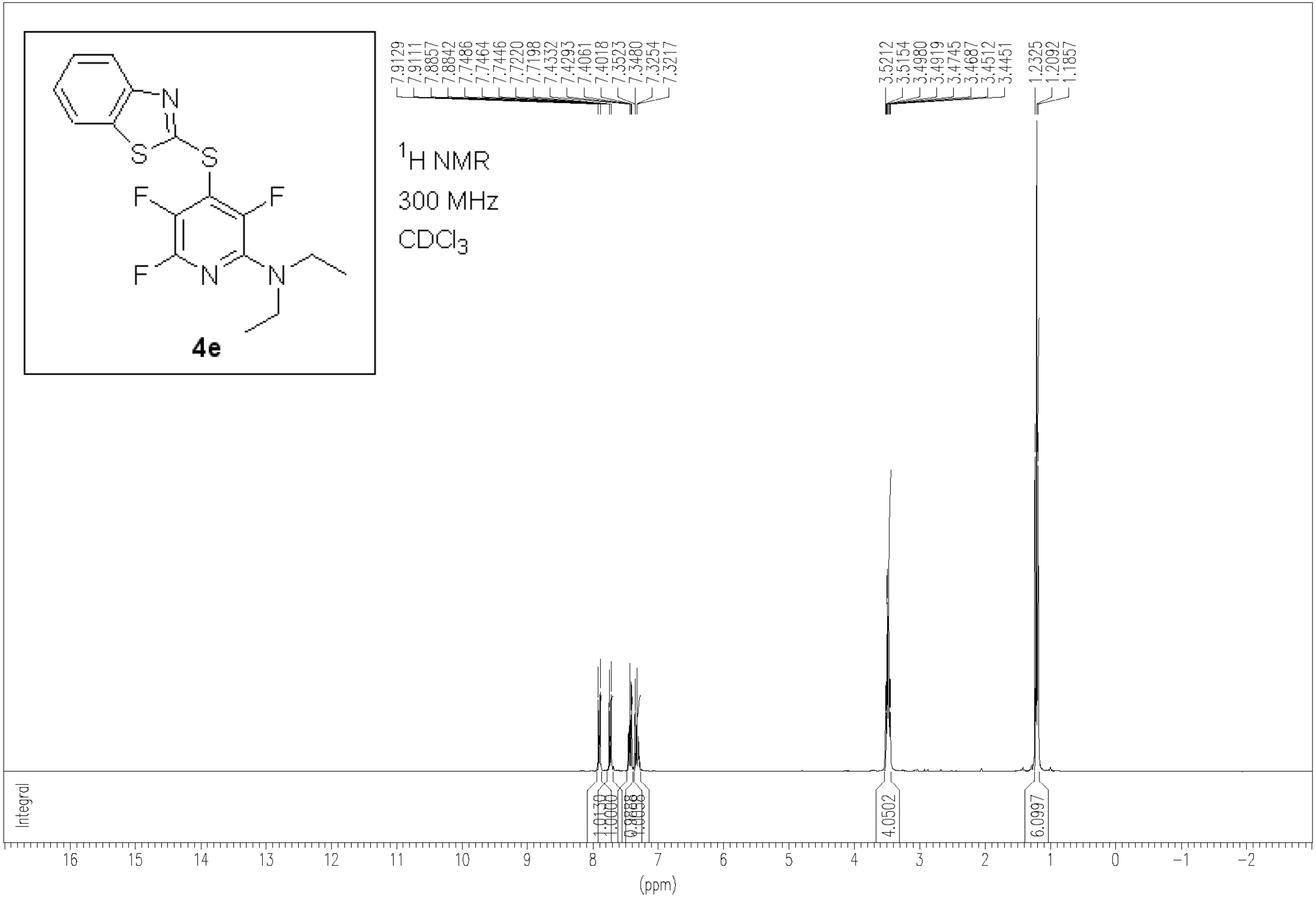

4e
1H NMR
300 MHz
CDCl3
7.9129
7.9111
7.8857
7.8842
7.7486
7.7464
7.7446
7.7220
7.7198
7.4332
7.4293
7.4061
7.4018
7.3523
7.3480
7.3254
7.3217
3.5212
3.5154
3.4980
3.4919
3.4745
3.4687
3.4512
3.4451
1.2325
1.2092
1.1857
Integral
4.0502
6.0997
(ppm)

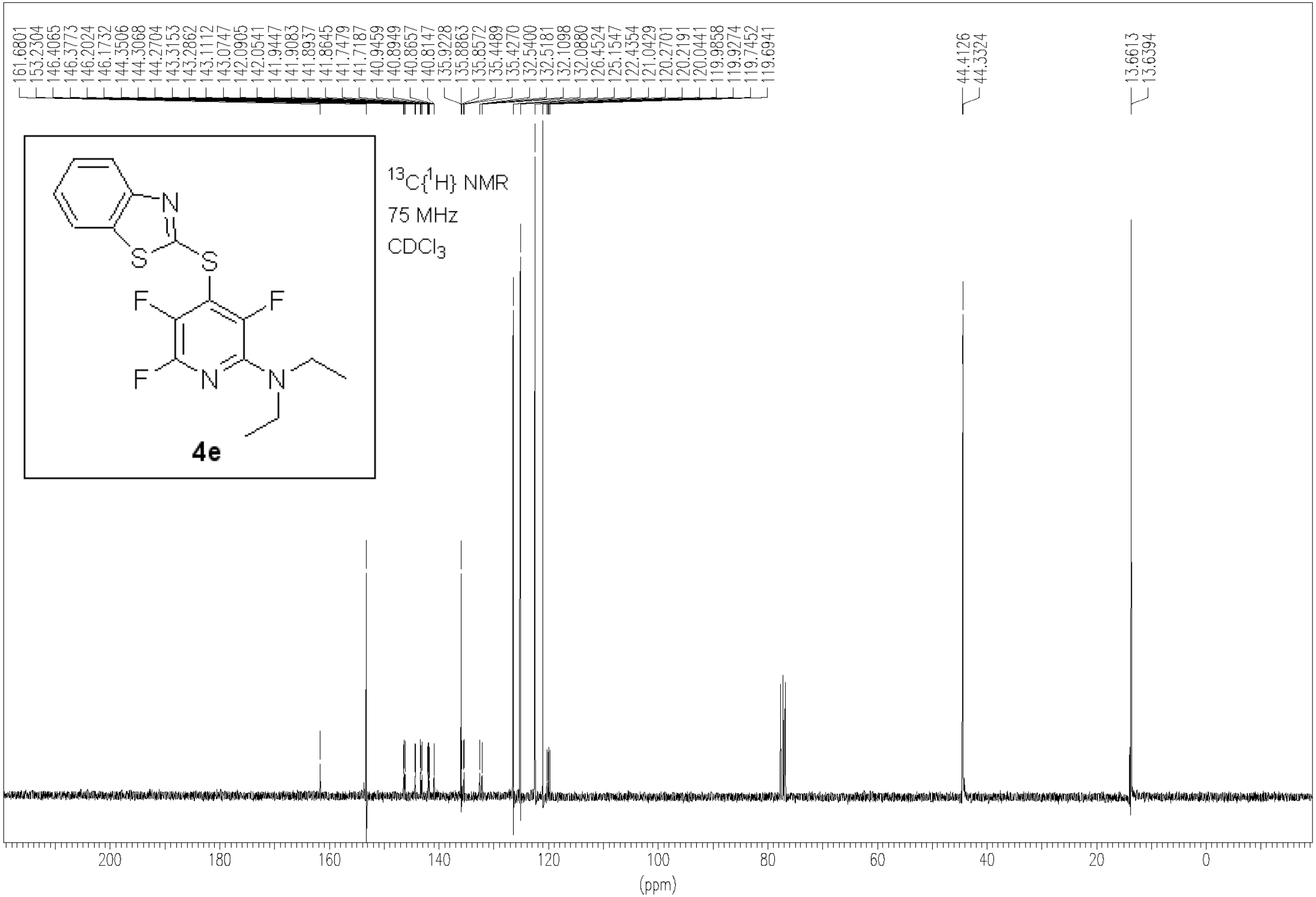
161.6801
153.2304
146.4065
146.3773
146.2024
146.1732
144.3506
144.3068
144.2704
143.3153
143.2862
143.1112
143.0747
142.0905
142.0541
141.9447
141.9083
141.8937
141.8645
141.7479
141.7187
140.9459
140.8949
140.8657
140.8147
135.9228
135.8863
135.8572
135.4489
135.4270
132.5400
132.5181
132.1098
132.0880
126.4524
125.1547
122.4354
121.0429
120.2701
120.2191
120.0441
119.9858
119.9274
119.7452
119.6941
44.4126
44.3324
13.6613
13.6394
$^{13}C\{^1H\}$ NMR
75 MHz
$CDCl_3$
N
S
S
F
F
F
N
N
4e
200
180
160
140
120
100
80
60
40
20
0
(ppm)

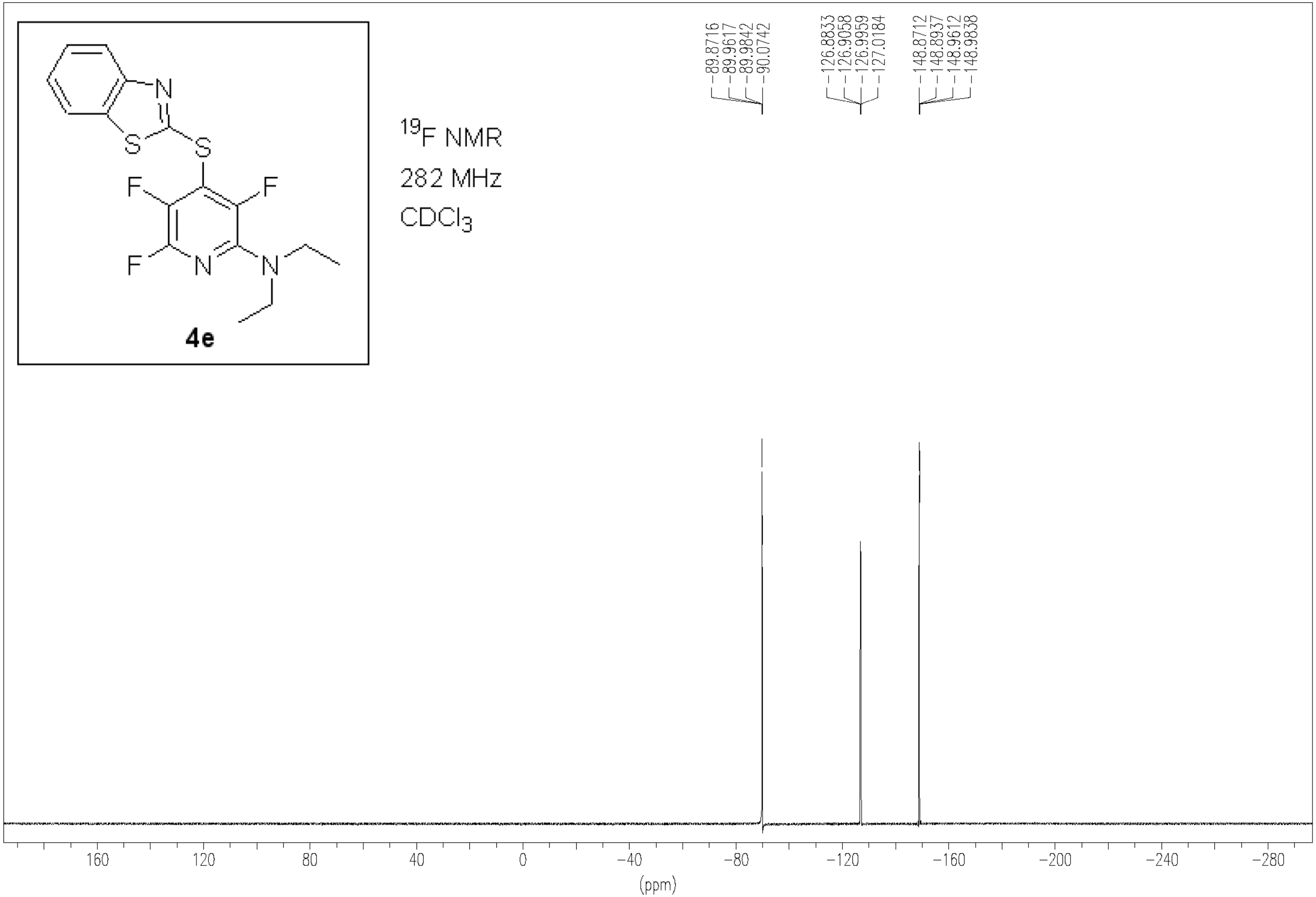
4e
19F NMR
282 MHz
CDCl3
-89.8716
-89.9617
-89.9842
-90.0742
-126.8833
-126.9058
-126.9959
-127.0184
-148.8712
-148.8937
-148.9612
-148.9838
160
120
80
40
0
−40
−80
−120
−160
−200
−240
−280
(ppm)

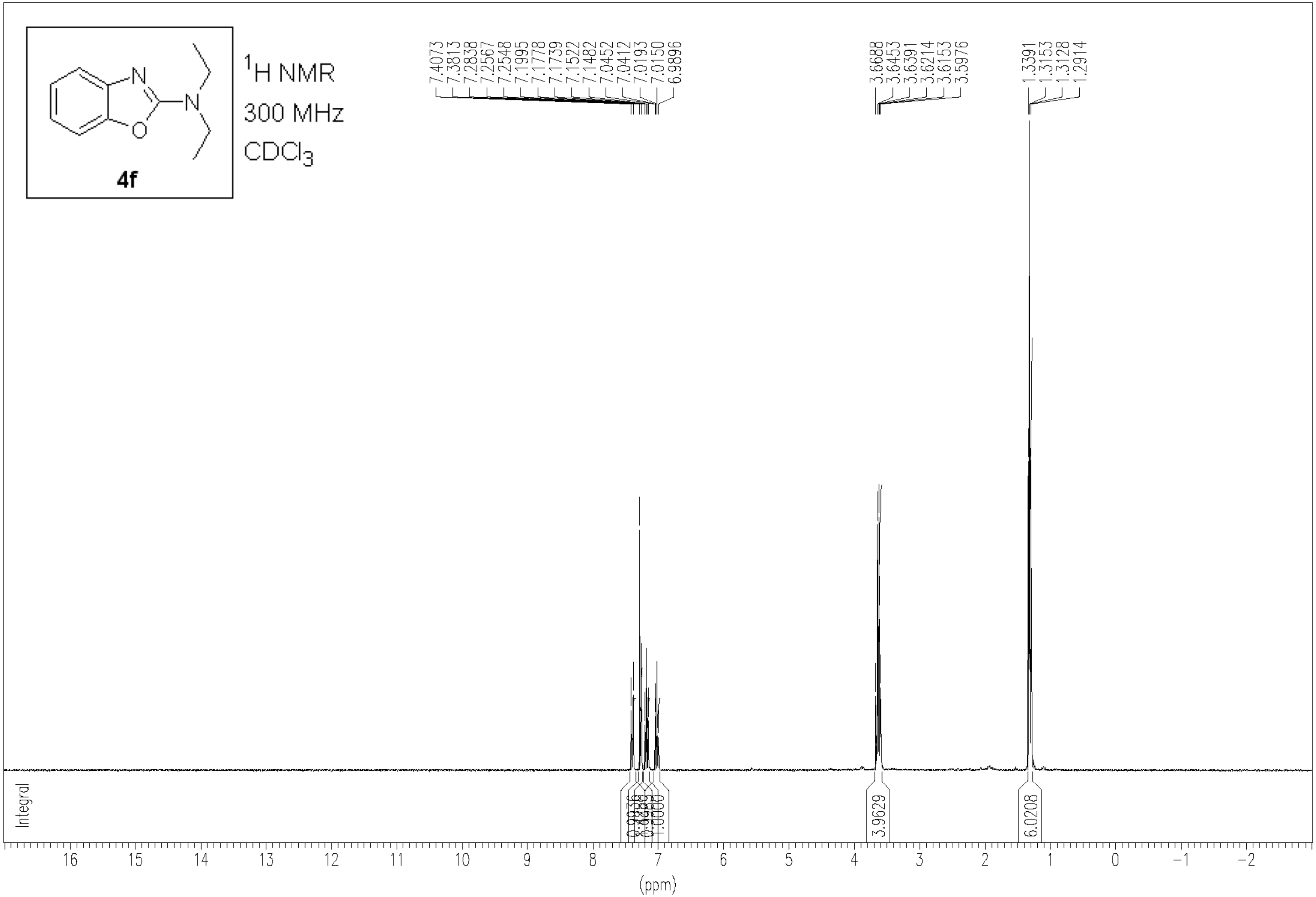
4f
^{1}H NMR
300 MHz
$CDCl_3$
7.4073
7.3813
7.2838
7.2567
7.2548
7.1995
7.1778
7.1739
7.1522
7.1482
7.0452
7.0412
7.0193
7.0150
6.9896
3.6688
3.6453
3.6391
3.6214
3.6153
3.5976
1.3391
1.3153
1.3128
1.2914
Integral
3.9629
6.0208
1.0000
16
15
14
13
12
11
10
9
8
7
6
5
4
3
2
1
0
−1
−2
(ppm)

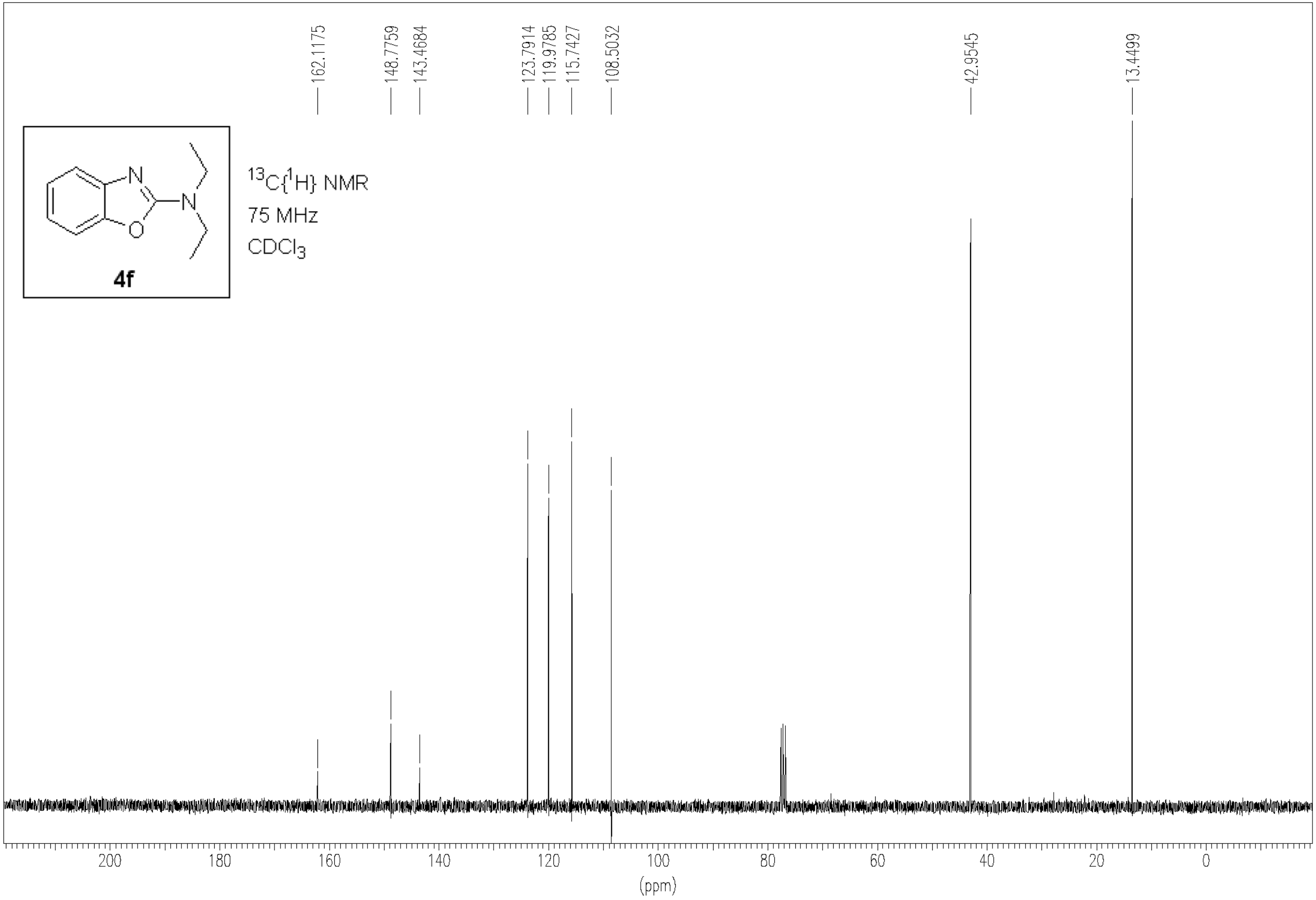

162.1175
148.7759
143.4684
123.7914
119.9785
115.7427
108.5032
42.9545
13.4499
4f
$^{13}C\{^1H\}$ NMR
75 MHz
$CDCl_3$
200
180
160
140
120
100
80
60
40
20
0
(ppm)

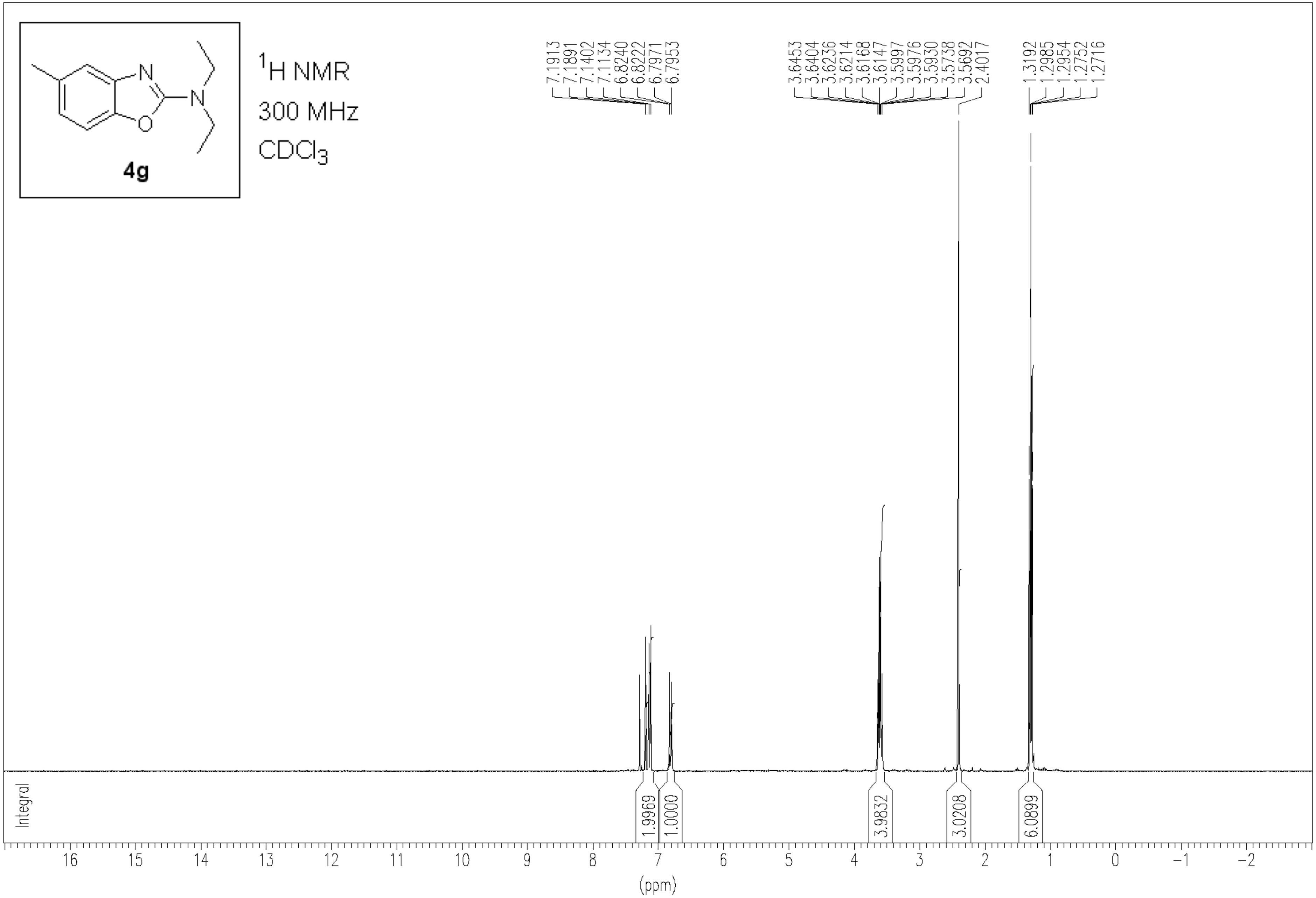

4g
1H NMR
300 MHz
CDCl3
7.1913
7.1891
7.1402
7.1134
6.8240
6.8222
6.7971
6.7953
3.6453
3.6404
3.6236
3.6214
3.6168
3.6147
3.5997
3.5976
3.5930
3.5738
3.5692
2.4017
1.3192
1.2985
1.2954
1.2752
1.2716
Integral
1.9969
1.0000
3.9832
3.0208
6.0899
(ppm)

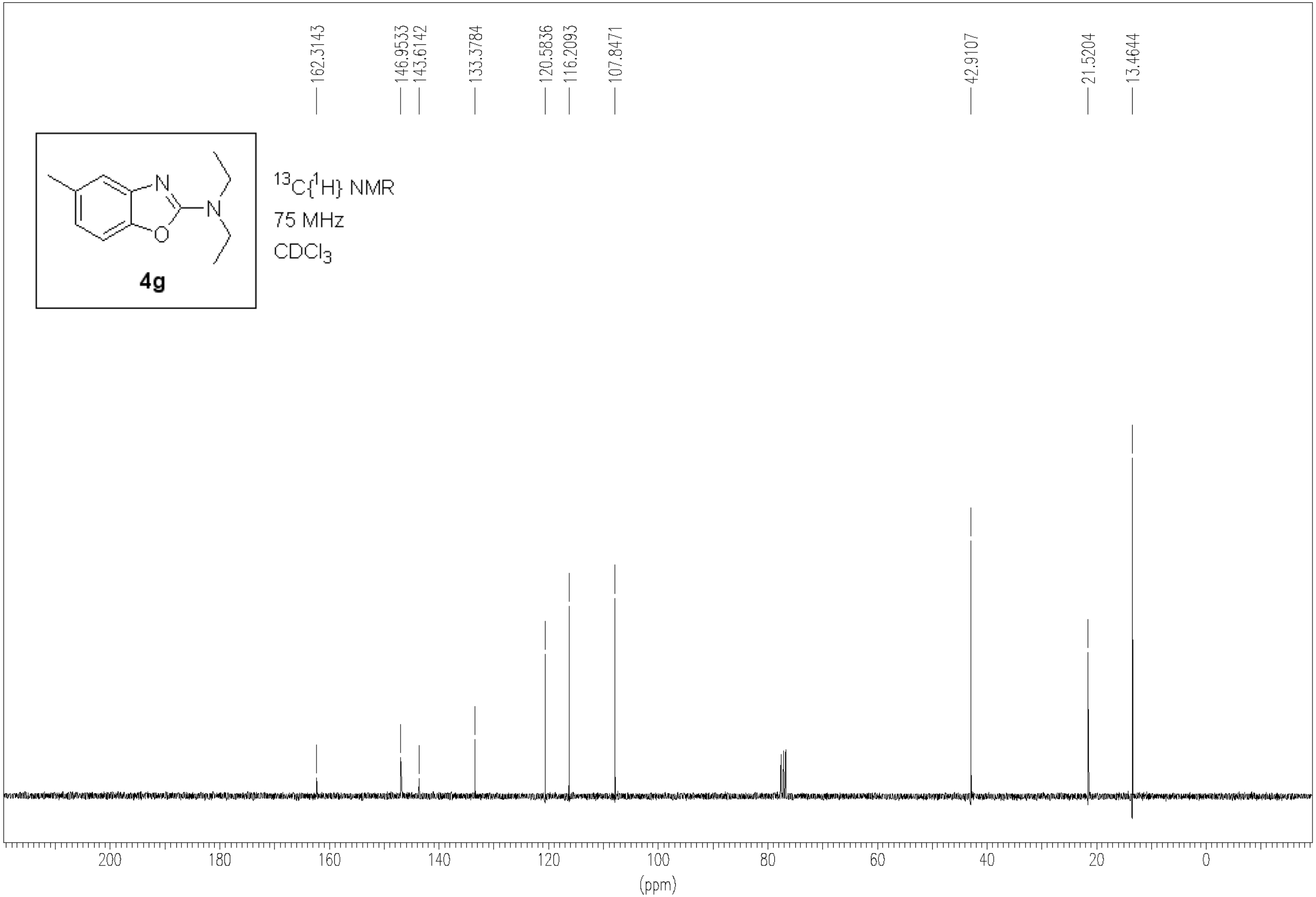
162.3143
146.9533
143.6142
133.3784
120.5836
116.2093
107.8471
42.9107
21.5204
13.4644
N
N
O
4g
13C{1H} NMR
75 MHz
CDCl3
200
180
160
140
120
100
80
60
40
20
0
(ppm)

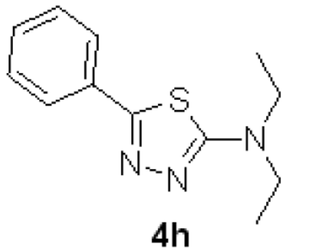

4h

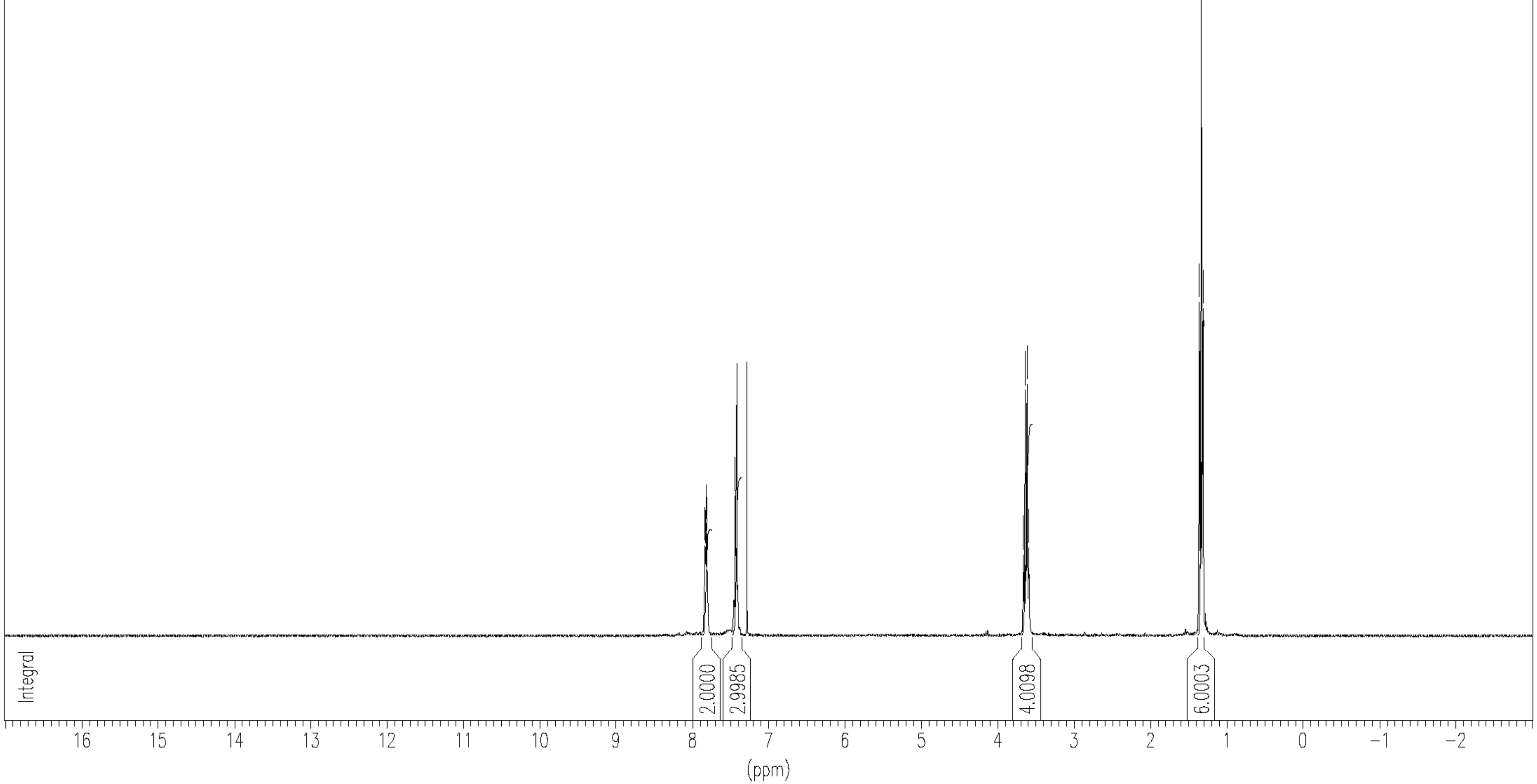

¹H NMR
300 MHz
CDCl3
7.8372
7.8298
7.8112
7.8066
7.4381
7.4189
7.4146
3.6614
3.6376
3.6141
3.5903
1.3553
1.3321
1.3082
Integral
2.0000
2.9985
4.0098
6.0003
16
15
14
13
12
11
10
9
8
7
6
5
4
3
2
1
0
−1
−2
(ppm)

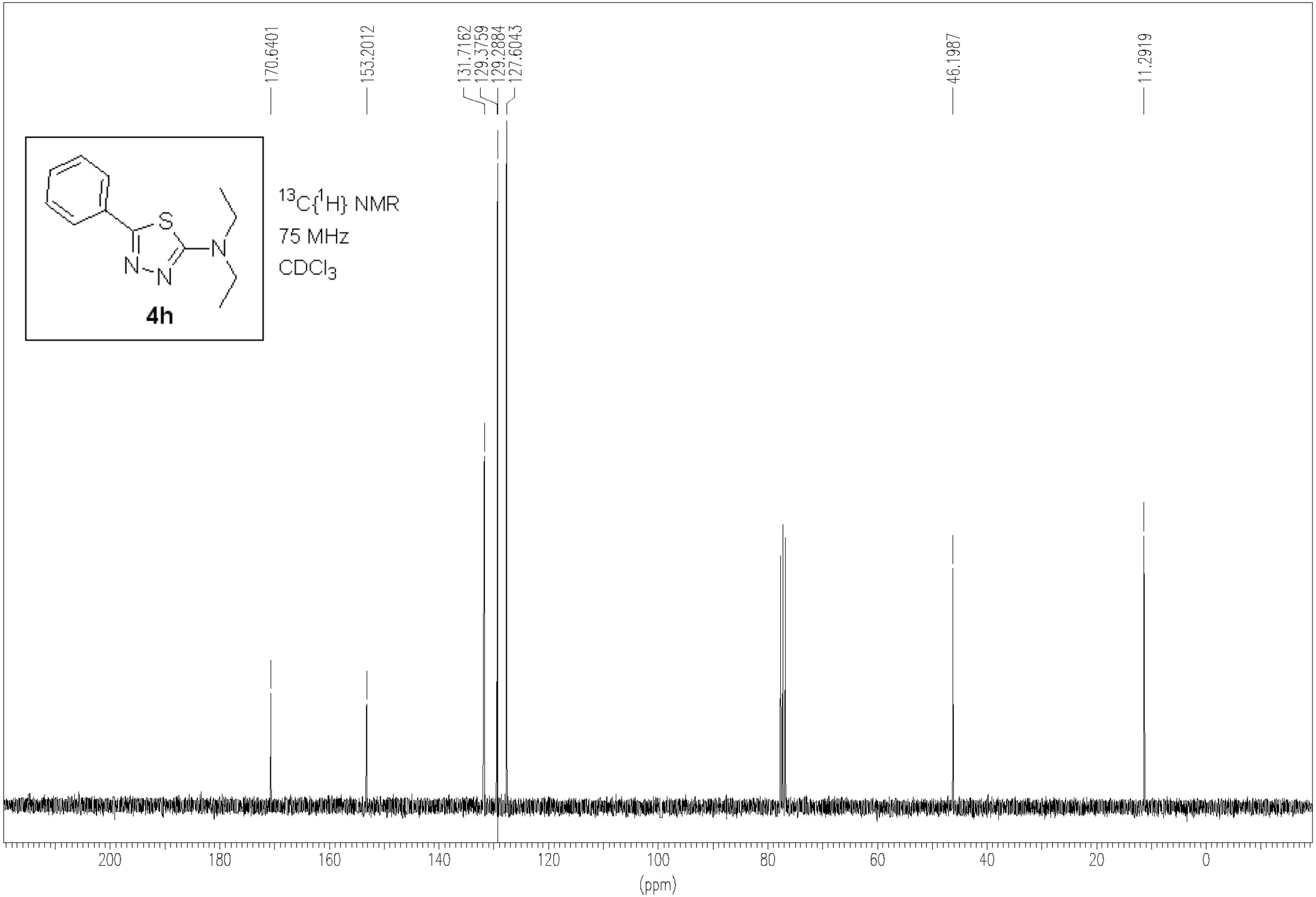
170.6401
153.2012
131.7162
129.3759
129.2884
127.6043
46.1987
11.2919
S
N
N
N
4h
$^{13}C\{^{1}H\}$ NMR
75 MHz
$CDCl_3$
200
180
160
140
120
100
80
60
40
20
0
(ppm)

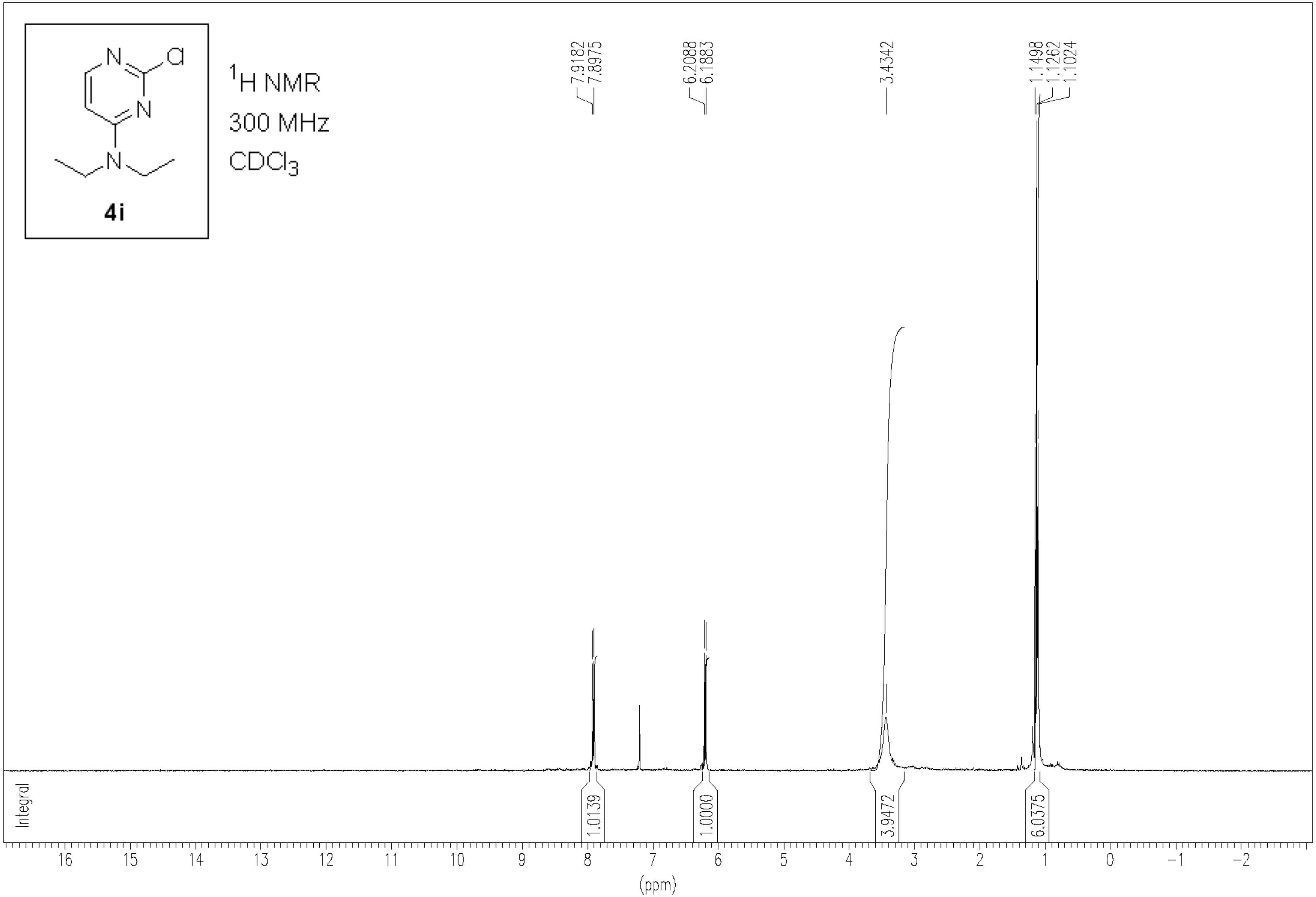
Cl
N
N
N
4i
^{1}H NMR
300 MHz
$CDCl_3$
7.9182
7.8975
6.2088
6.1883
3.4342
1.1498
1.1262
1.1024
Integral
1.0139
1.0000
3.9472
6.0375
16
15
14
13
12
11
10
9
8
7
6
5
4
3
2
1
0
−1
−2
(ppm)

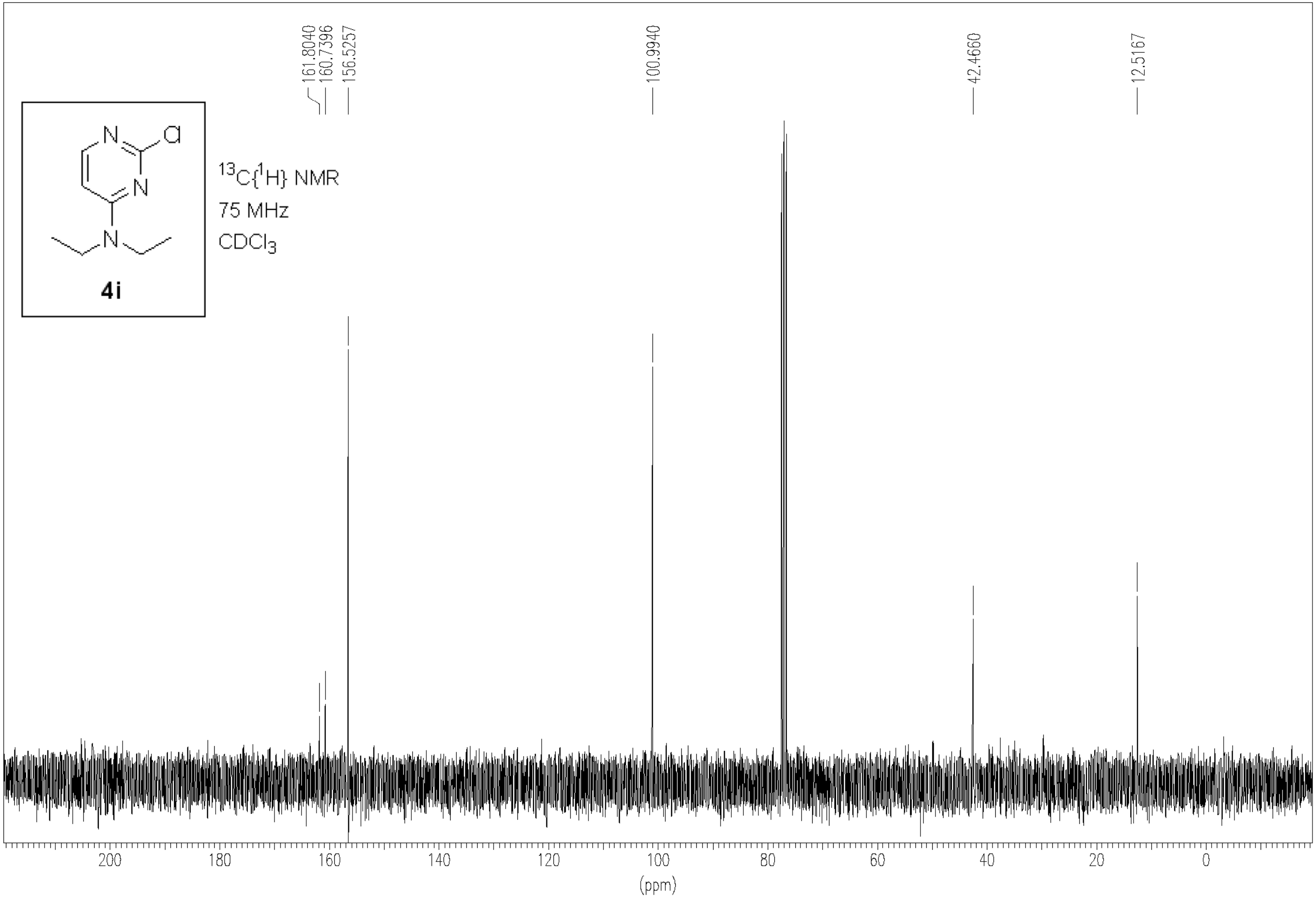
161.8040
160.7396
156.5257
100.9940
42.4660
12.5167
Cl
N
N
N
4i
$^{13}C\{^1H\}$ NMR
75 MHz
$CDCl_3$
200
180
160
140
120
100
80
60
40
20
0
(ppm)

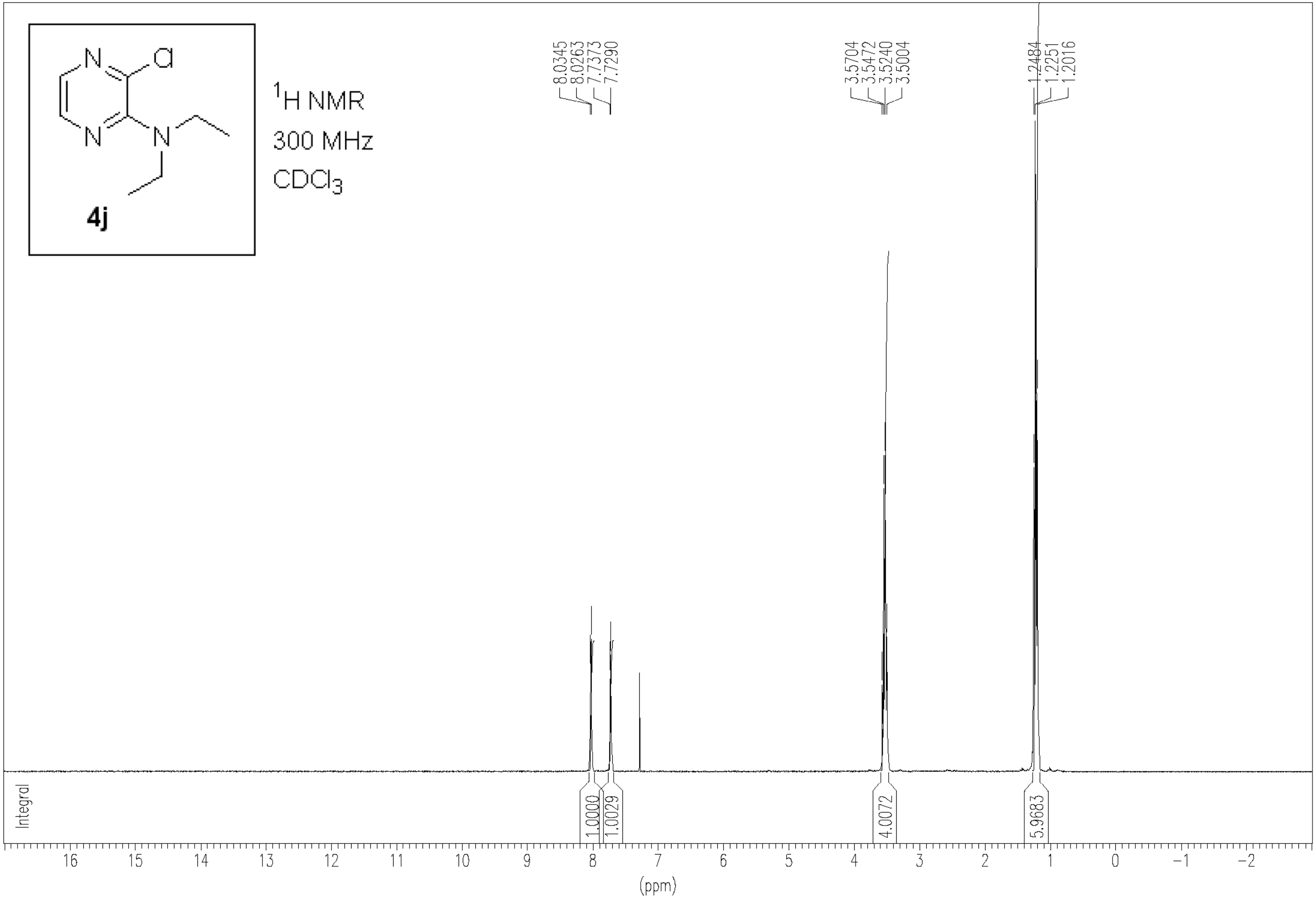
4j
1H NMR
300 MHz
CDCl3
8.0345
8.0263
7.7373
7.7290
3.5704
3.5472
3.5240
3.5004
1.2484
1.2251
1.2016
Integral
1.0000
1.0029
4.0072
5.9683
(ppm)

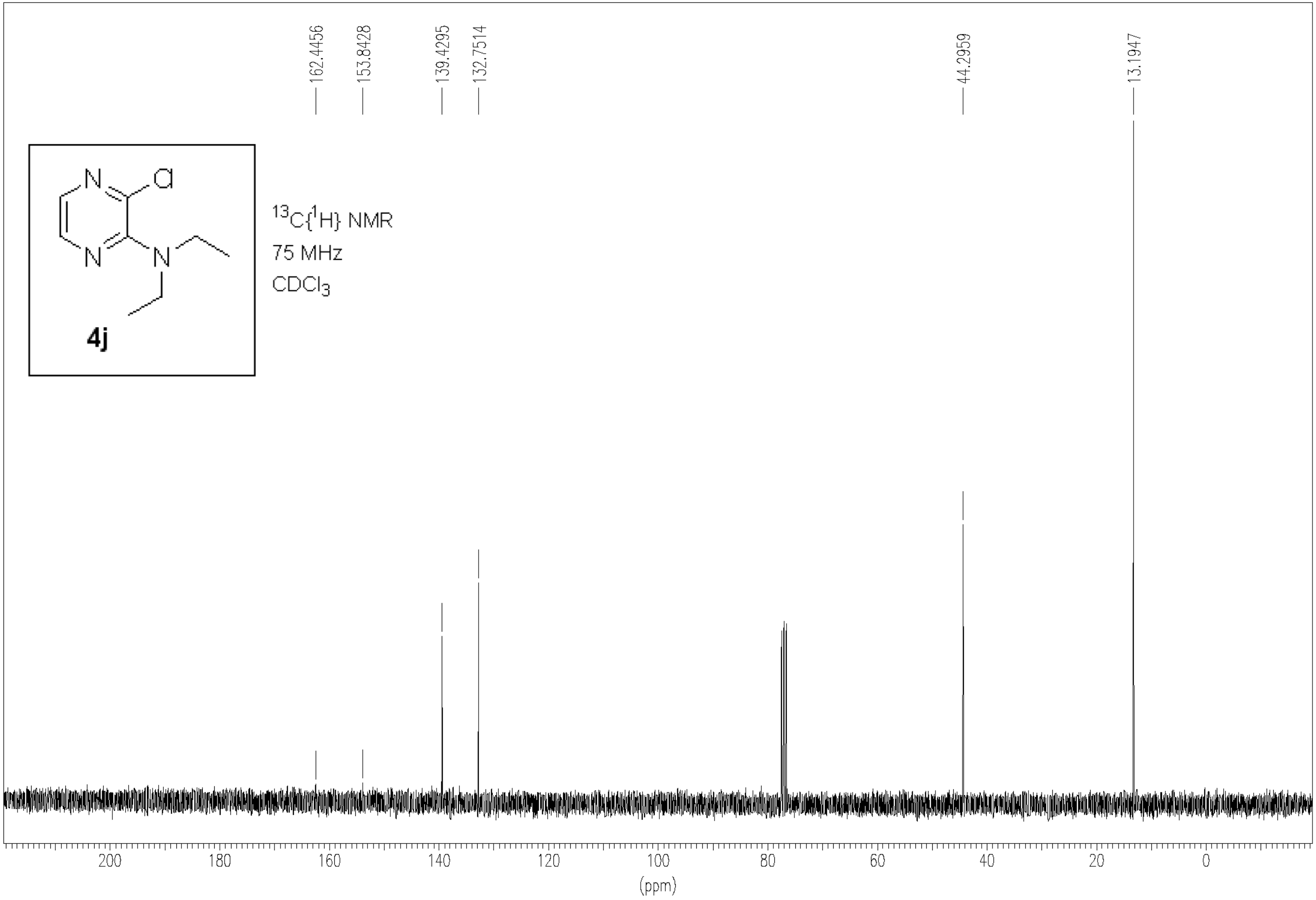
162.4456
153.8428
139.4295
132.7514
44.2959
13.1947
N
Cl
N
N
4j
$^{13}C\{^1H\}$ NMR
75 MHz
$CDCl_3$
200
180
160
140
120
100
80
60
40
20
0
(ppm)

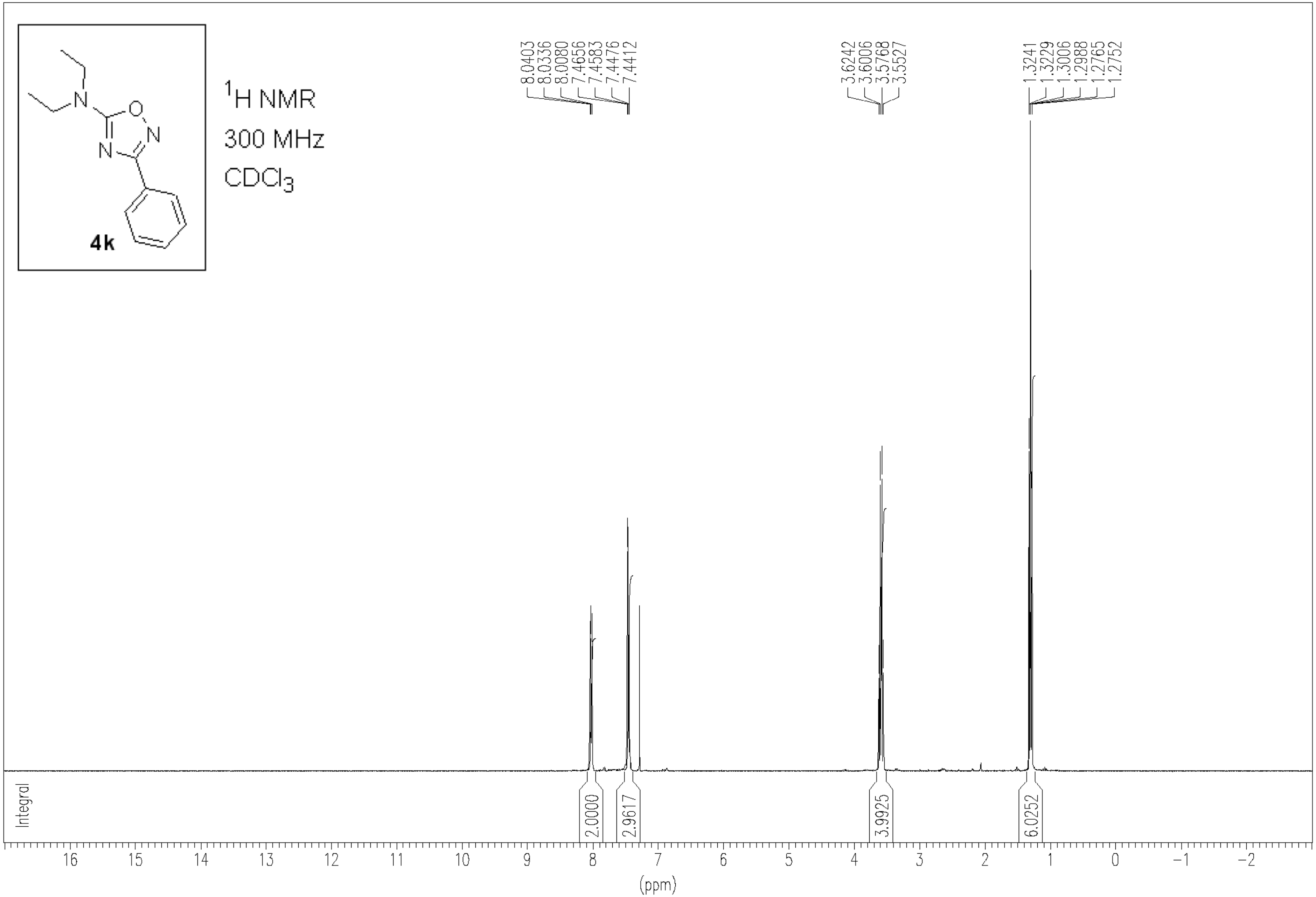

4k
^{1}H NMR
300 MHz
$CDCl_3$
8.0403
8.0336
8.0080
7.4656
7.4583
7.4476
7.4412
3.6242
3.6006
3.5768
3.5527
1.3241
1.3229
1.3006
1.2988
1.2765
1.2752
Integral
2.0000
2.9617
3.9925
6.0252
16 15 14 13 12 11 10 9 8 7 6 5 4 3 2 1 0 −1 −2
(ppm)

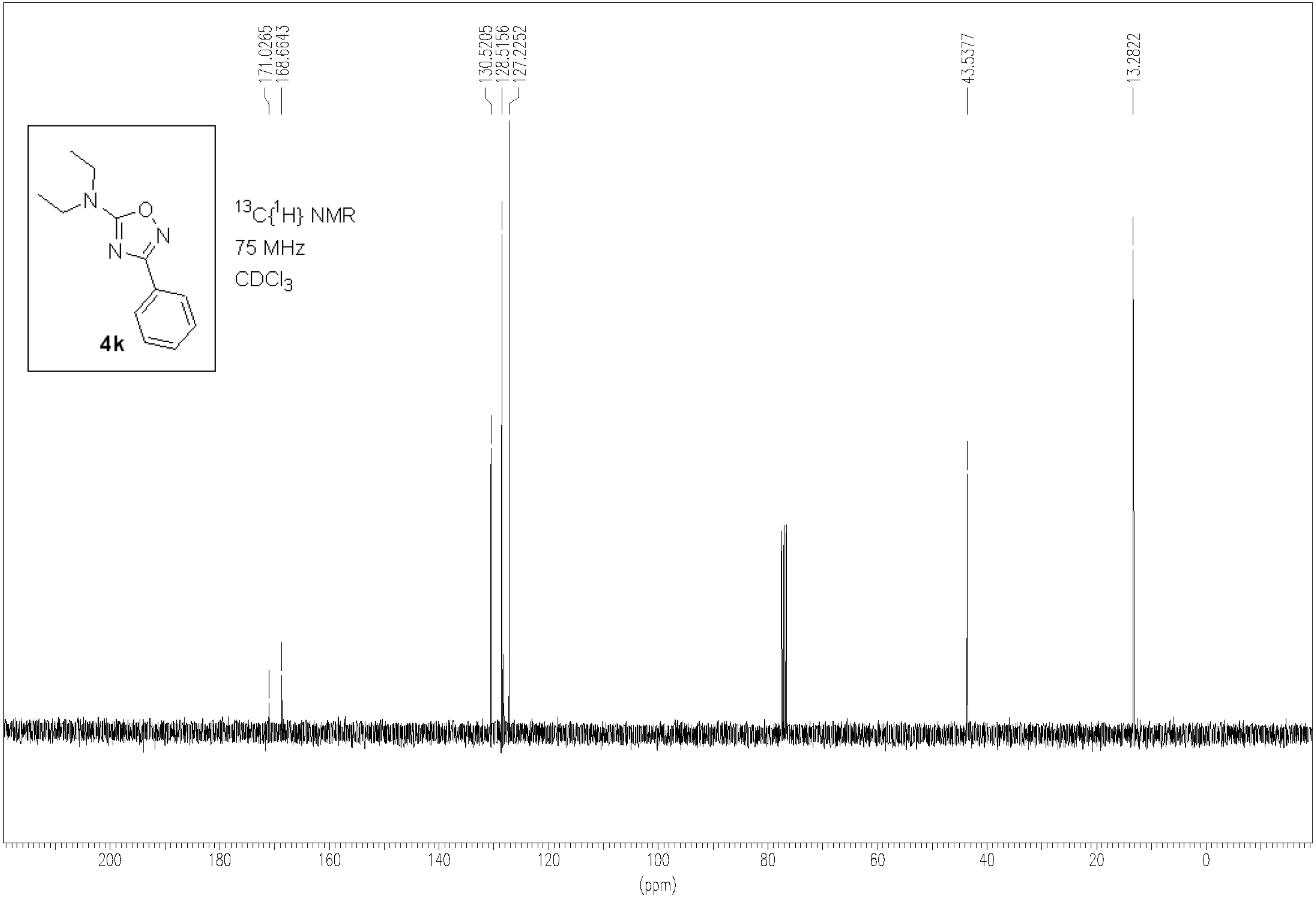

171.0265
168.6643
130.5205
128.5156
127.2252
43.5377
13.2822
4k
$^{13}C\{^1H\}$ NMR
75 MHz
$CDCl_3$
200
180
160
140
120
100
80
60
40
20
0
(ppm)

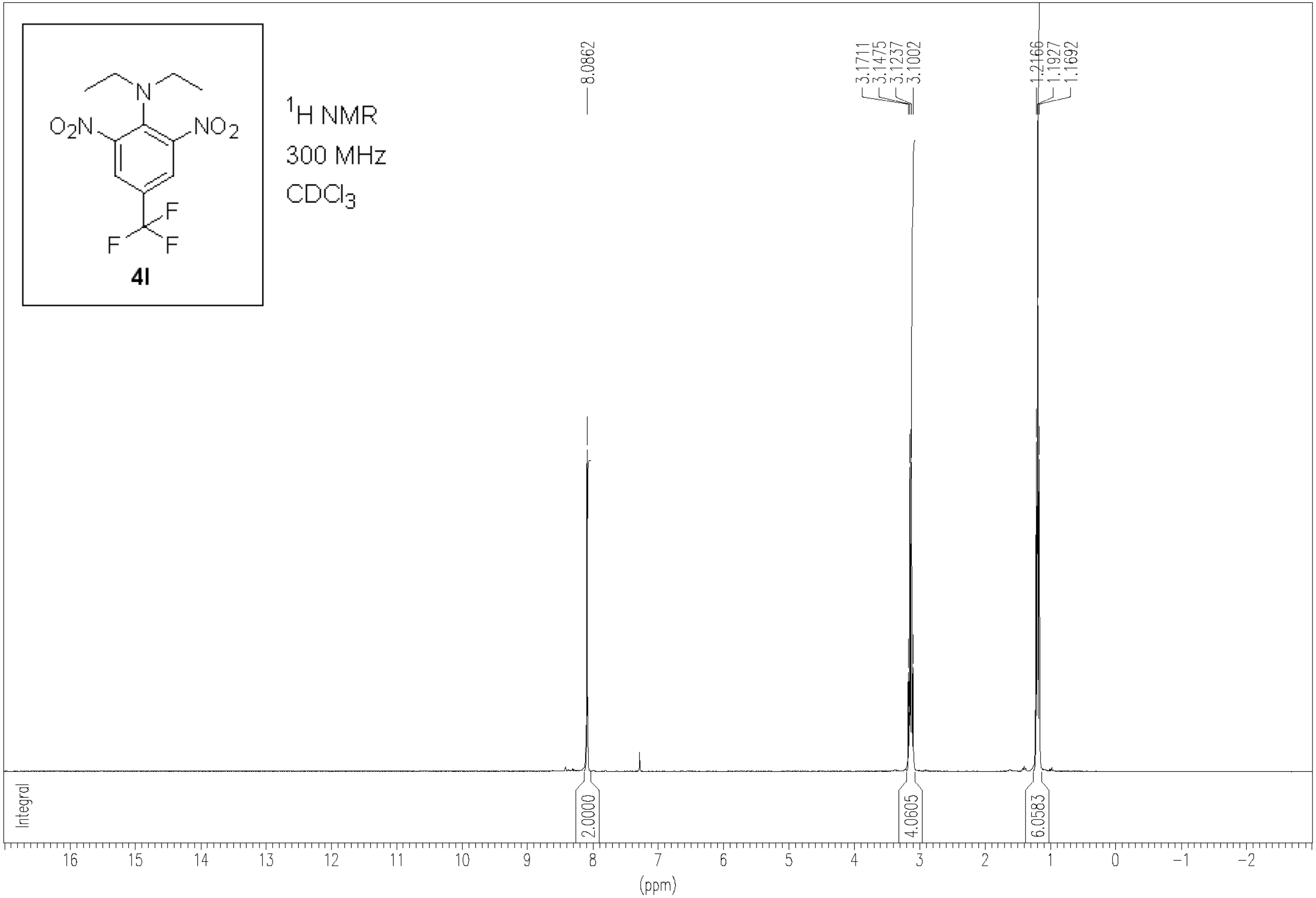
O2N
NO2
N
F
F
F
4l
1H NMR
300 MHz
CDCl3
8.0862
3.1711
3.1475
3.1237
3.1002
1.2166
1.1927
1.1692
Integral
2.0000
4.0605
6.0583
16
15
14
13
12
11
10
9
8
7
6
5
4
3
2
1
0
−1
−2
(ppm)

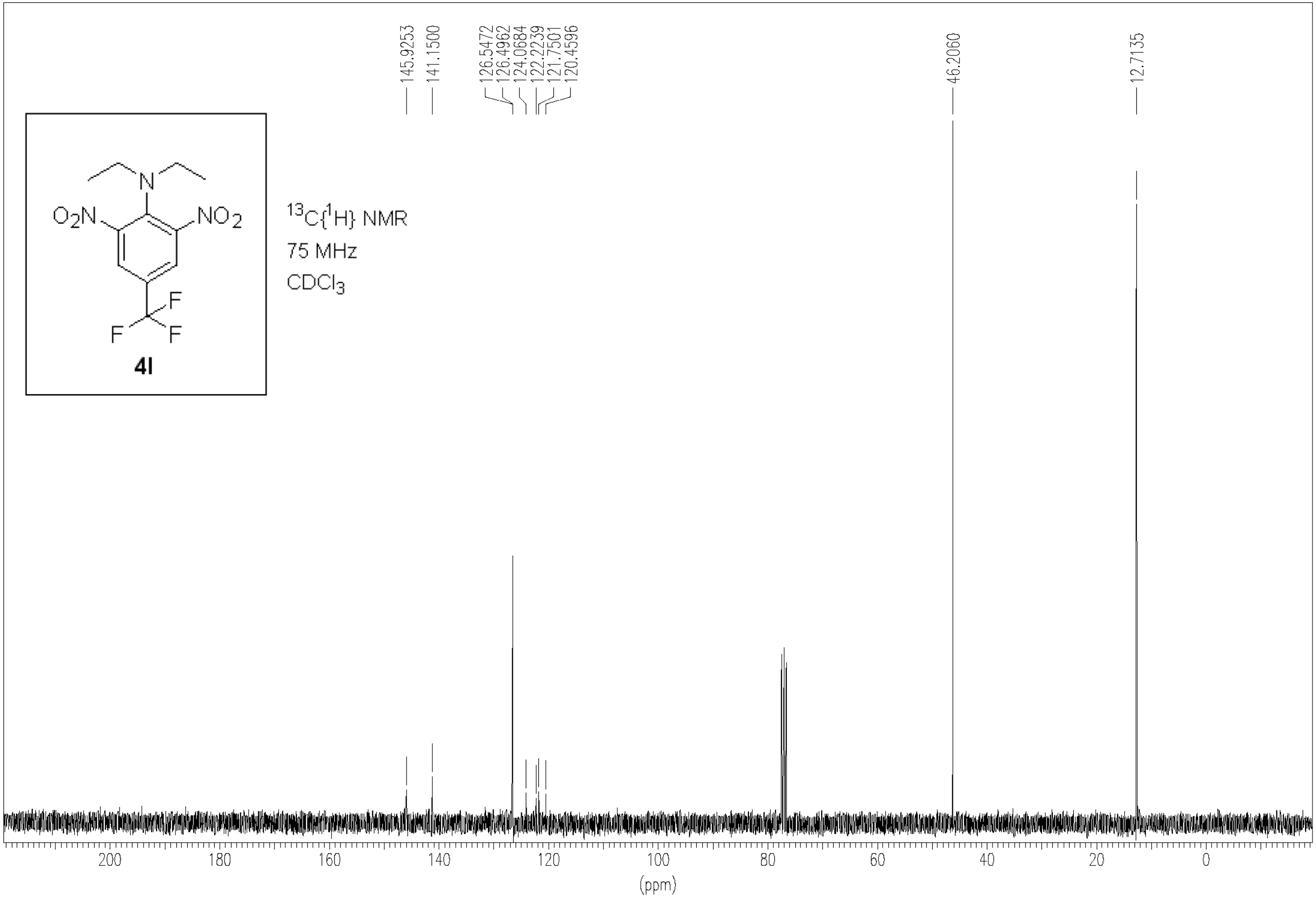
145.9253
141.1500
126.5472
126.4962
124.0684
122.2239
121.7501
120.4596
46.2060
12.7135
O2N
NO2
N
F
F
F
4l
13C{1H} NMR
75 MHz
CDCl3
200
180
160
140
120
100
80
60
40
20
0
(ppm)

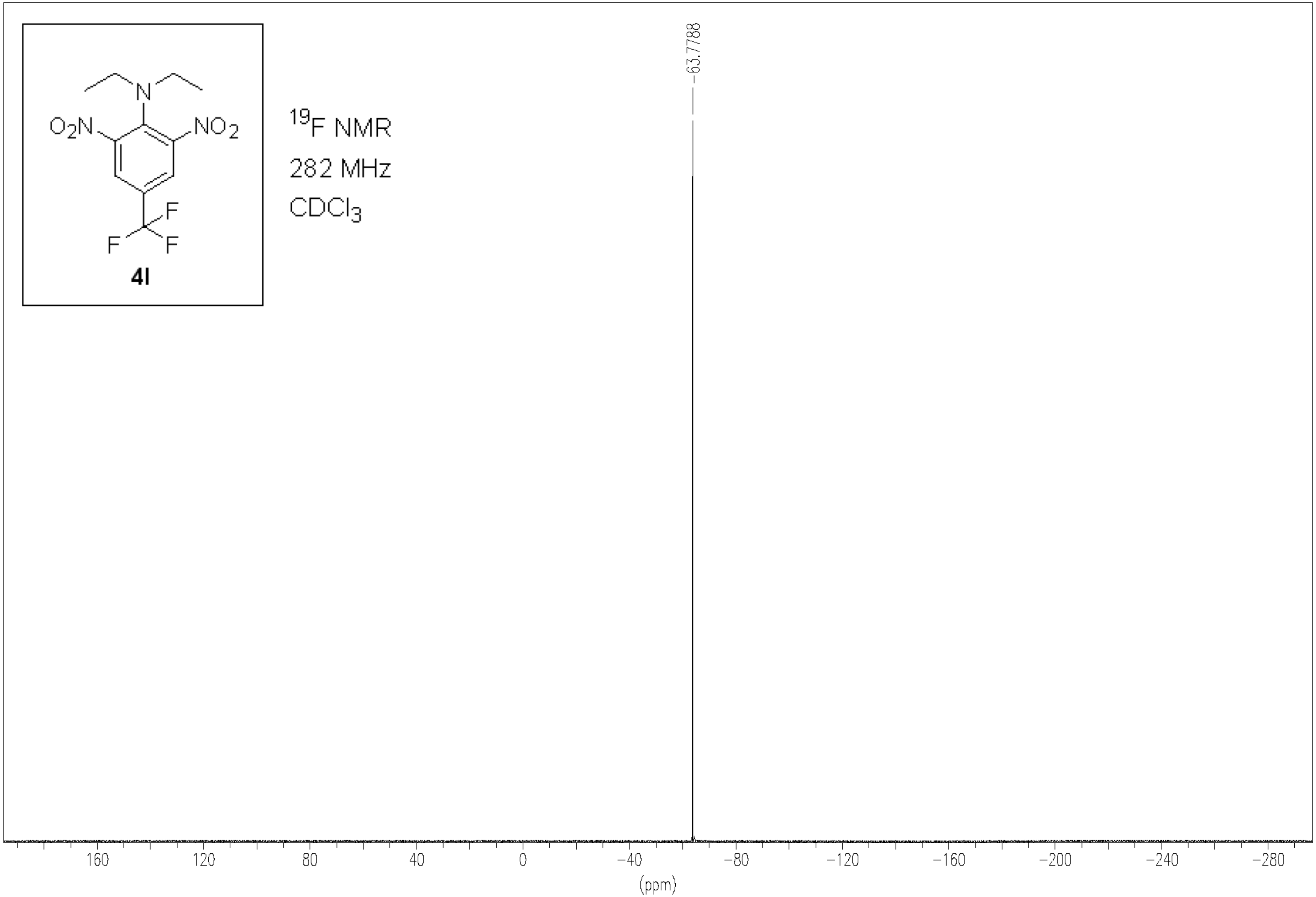
4l
^{19}F NMR
282 MHz
$CDCl_3$
−63.7788
160
120
80
40
0
−40
−80
−120
−160
−200
−240
−280
(ppm)

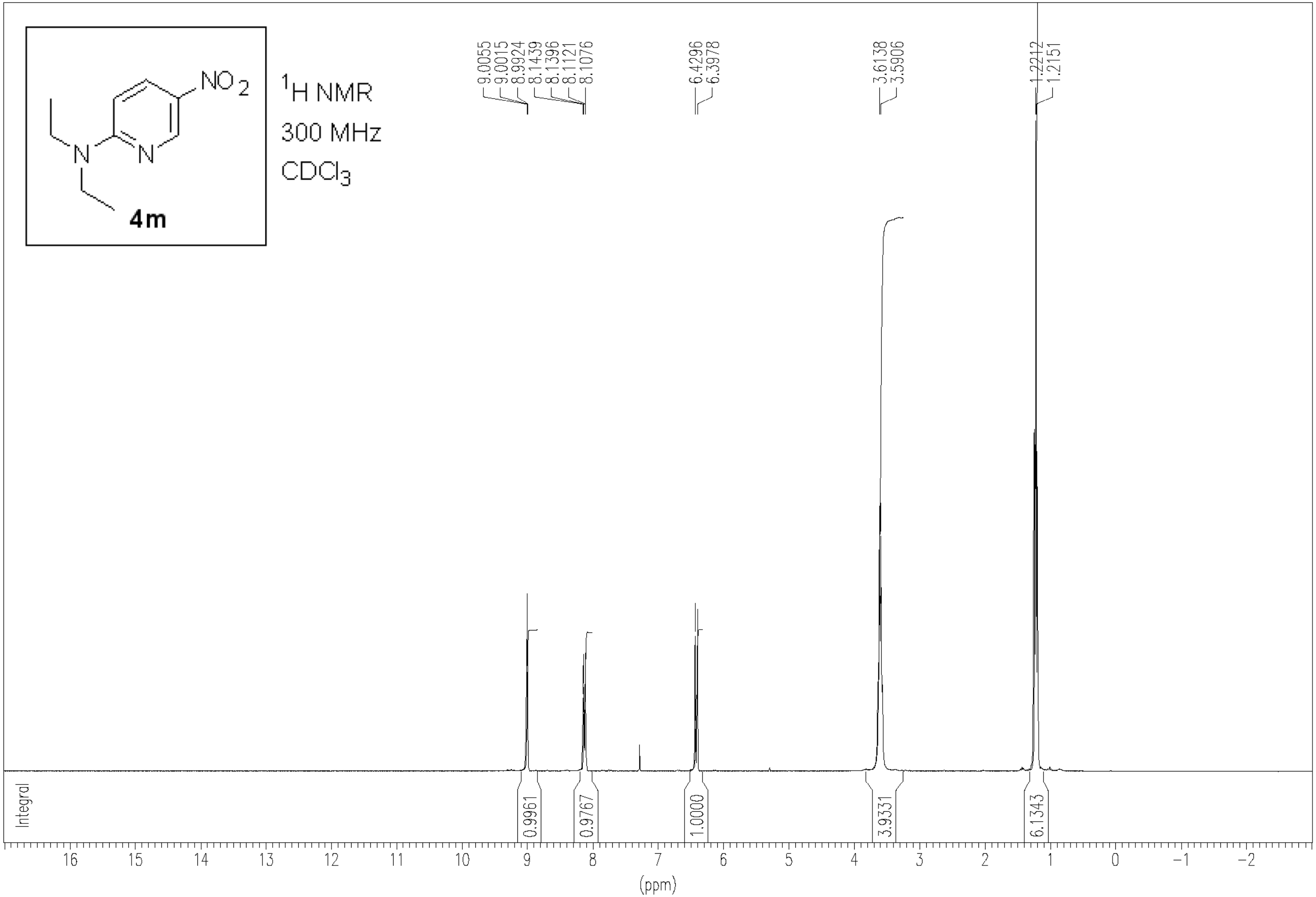
NO2
N
N
4m
1H NMR
300 MHz
CDCl3
9.0055
9.0015
8.9924
8.1439
8.1396
8.1121
8.1076
6.4296
6.3978
3.6138
3.5906
1.2212
1.2151
Integral
0.9961
0.9767
1.0000
3.9331
6.1343
16
15
14
13
12
11
10
9
8
7
6
5
4
3
2
1
0
−1
−2
(ppm)

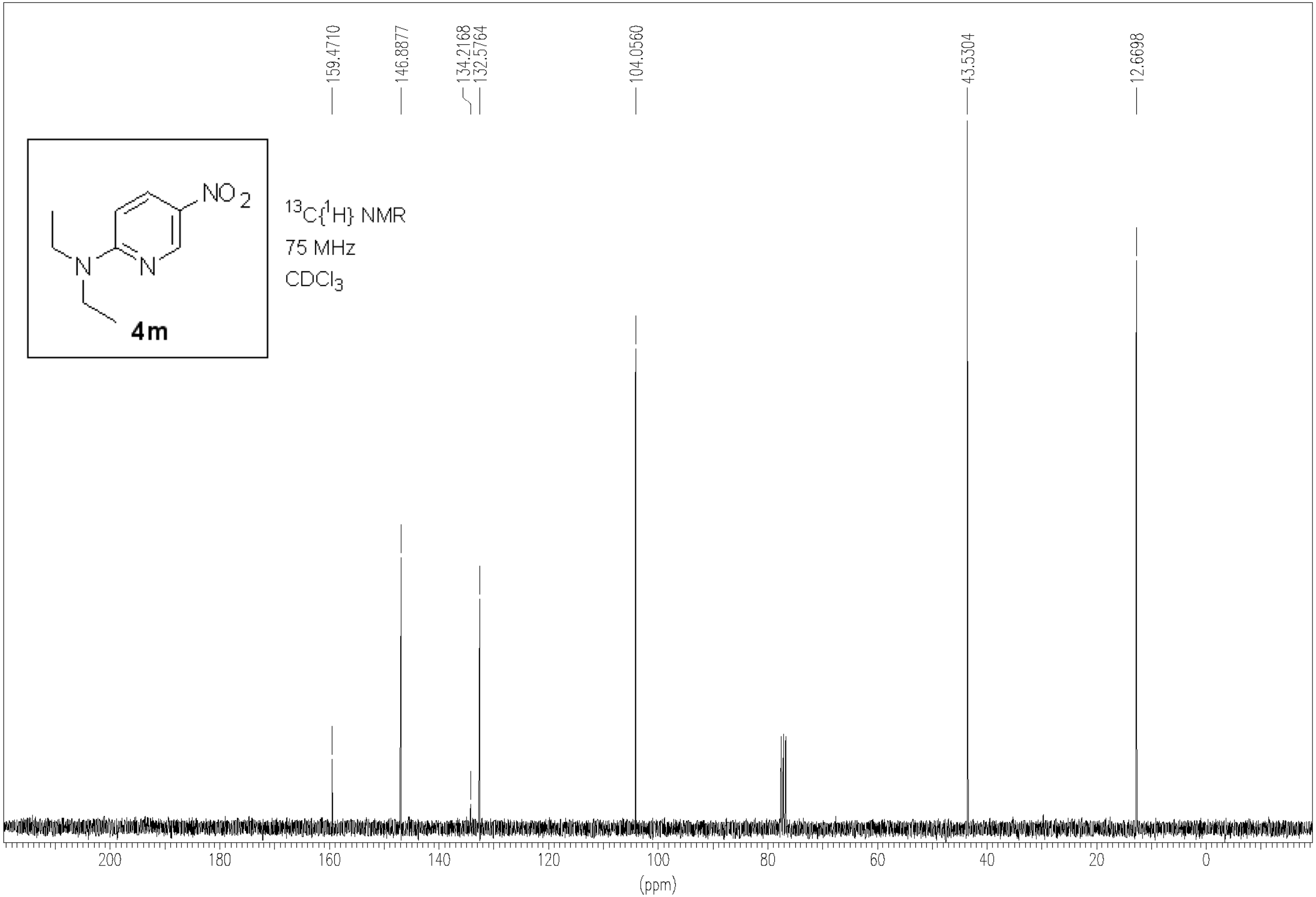
159.4710
146.8877
134.2168
132.5764
104.0560
43.5304
12.6698
NO2
N
N
4m
13C{1H} NMR
75 MHz
CDCl3
200
180
160
140
120
100
80
60
40
20
0
(ppm)

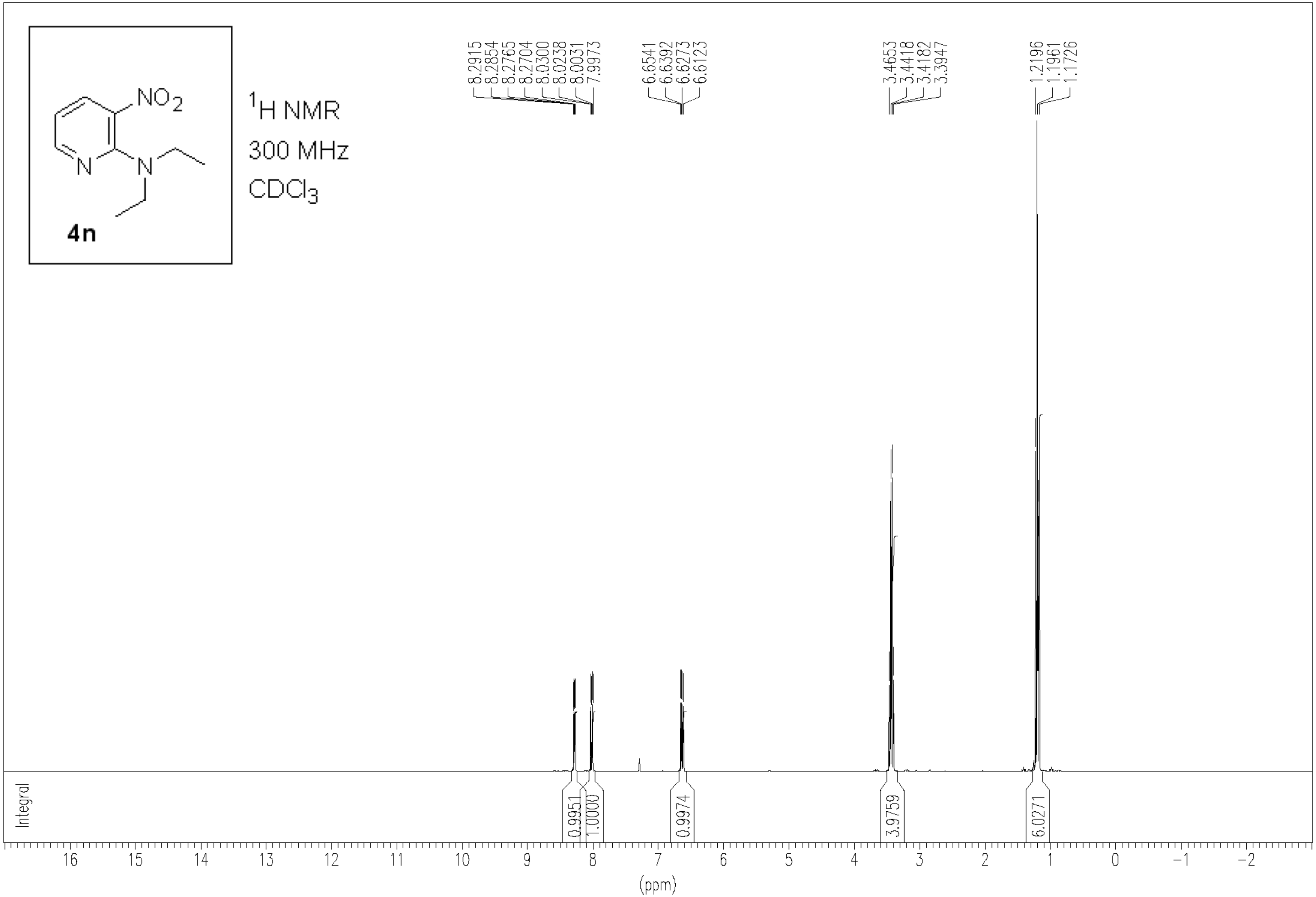

4n
¹H NMR
300 MHz
CDCl3
8.2915
8.2854
8.2765
8.2704
8.0300
8.0238
8.0031
7.9973
6.6541
6.6392
6.6273
6.6123
3.4653
3.4418
3.4182
3.3947
1.2196
1.1961
1.1726
Integral
0.9951
1.0000
0.9974
3.9759
6.0271
(ppm)

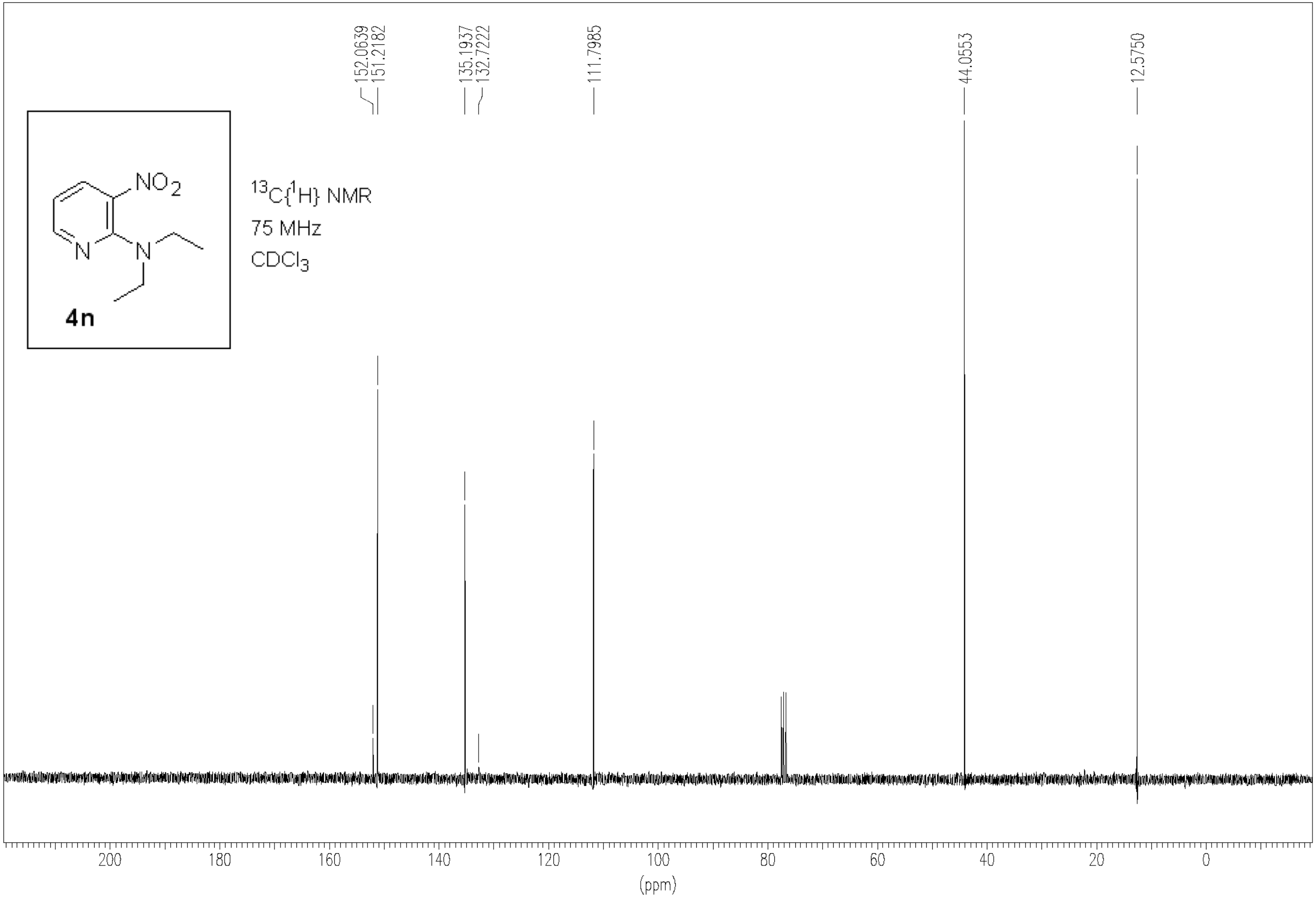

152.0639
151.2182
135.1937
132.7222
111.7985
44.0553
12.5750
NO2
N
N
4n
13C{1H} NMR
75 MHz
CDCl3
200
180
160
140
120
100
80
60
40
20
0
(ppm)

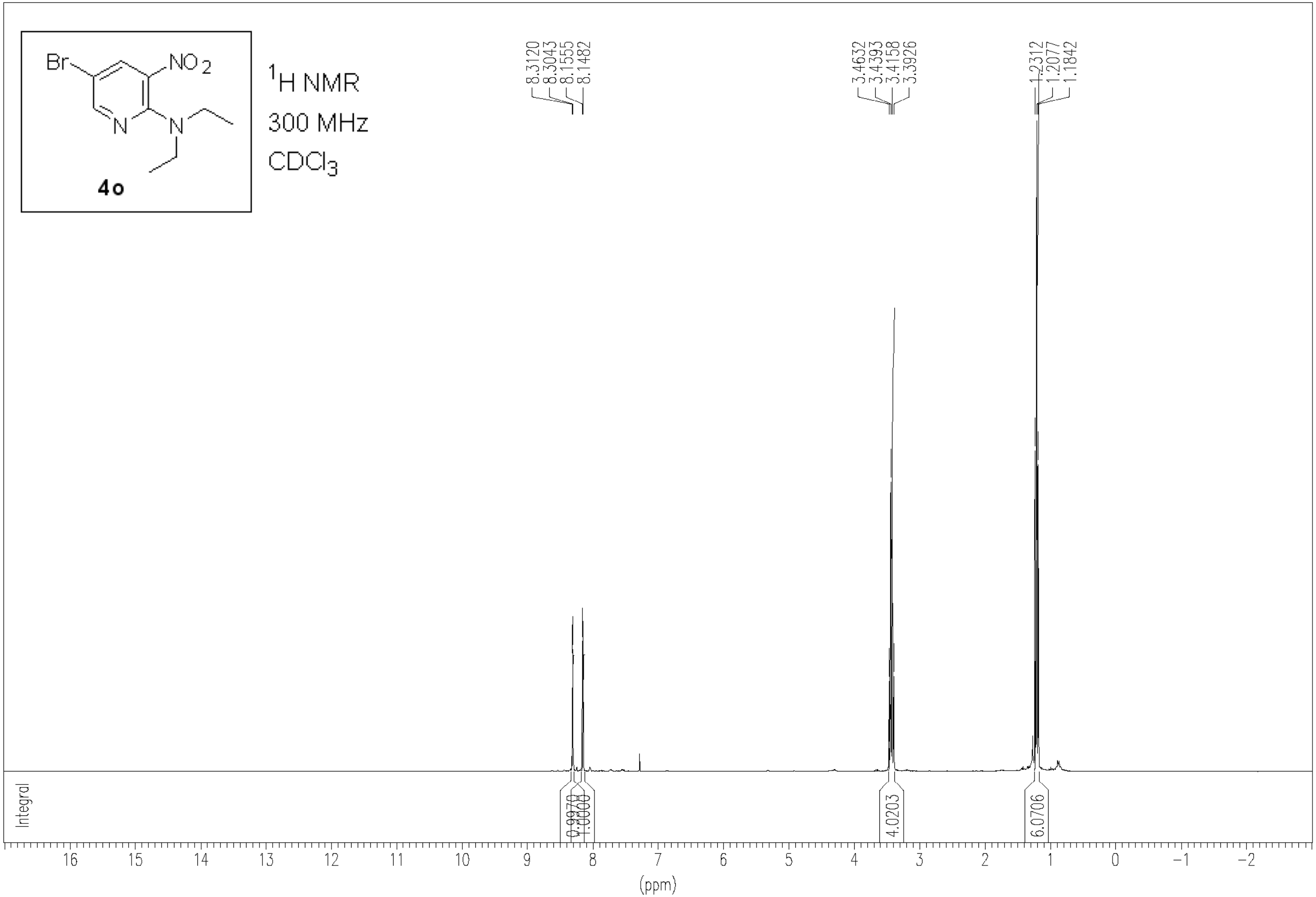

Br
NO_2
N
N
4o
^{1}H NMR
300 MHz
$CDCl_3$
8.3120
8.3043
8.1555
8.1482
3.4632
3.4393
3.4158
3.3926
1.2312
1.2077
1.1842
Integral
0.9970
1.0000
4.0203
6.0706
16 15 14 13 12 11 10 9 8 7 6 5 4 3 2 1 0 −1 −2
(ppm)

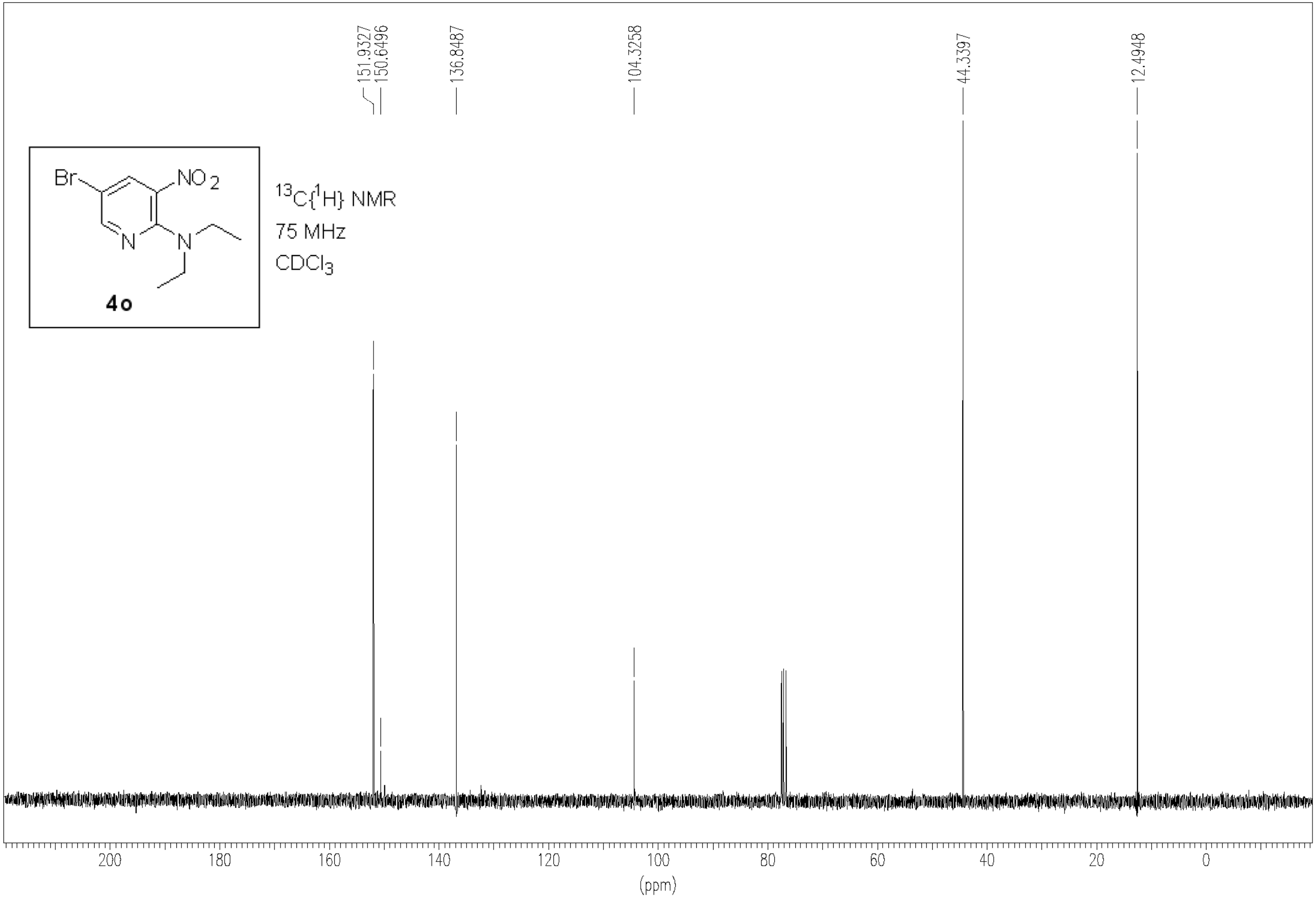
151.9327
150.6496
136.8487
104.3258
44.3397
12.4948
Br
NO2
N
N
4o
13C{1H} NMR
75 MHz
CDCl3
200
180
160
140
120
100
80
60
40
20
0
(ppm)

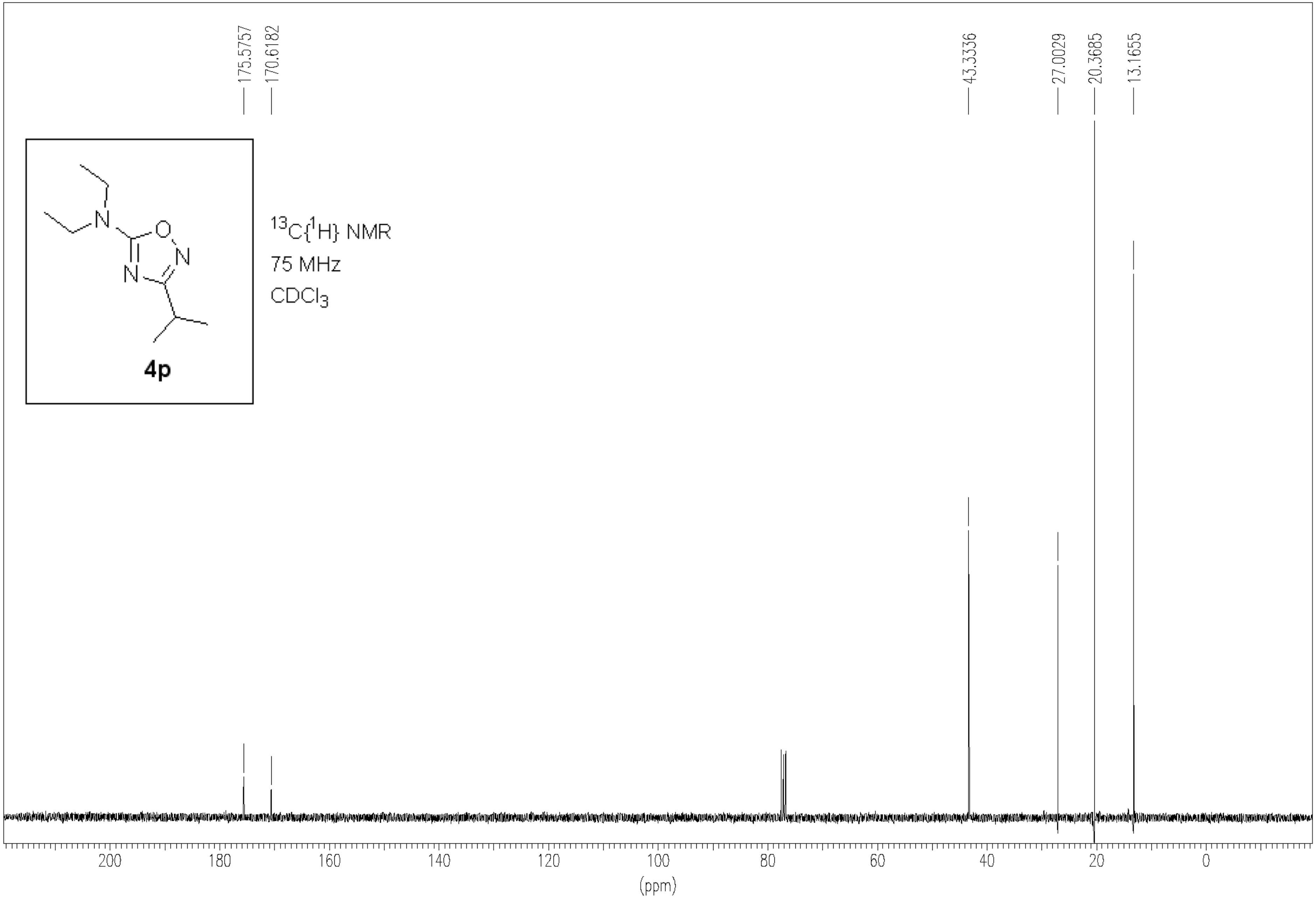
175.5757
170.6182
43.3336
27.0029
20.3685
13.1655
N
O
N
N
4p
$^{13}C\{^1H\}$ NMR
75 MHz
$CDCl_3$
200
180
160
140
120
100
80
60
40
20
0
(ppm)

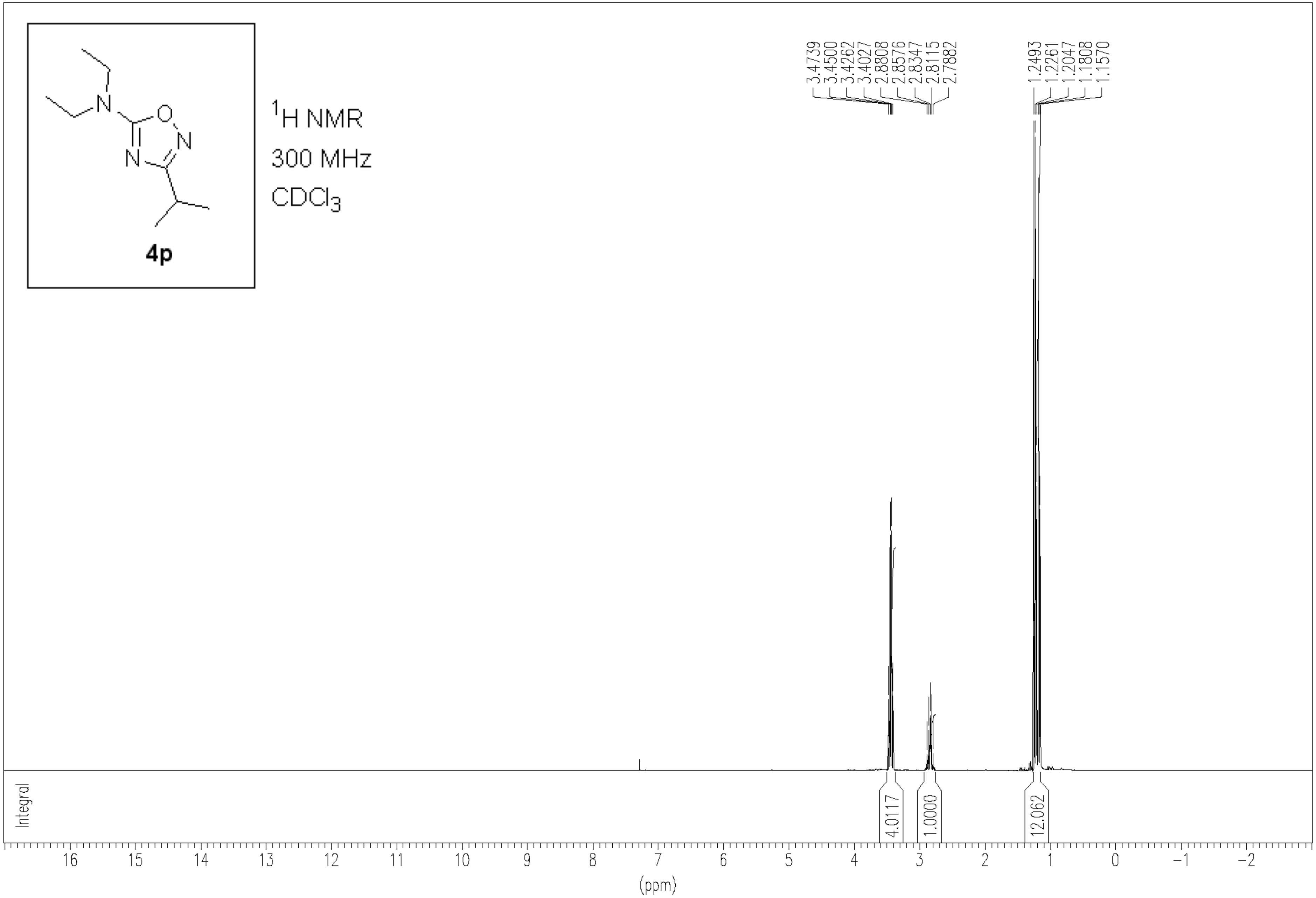

4p
^{1}H NMR
300 MHz
CDCl$_3$
3.4739
3.4500
3.4262
3.4027
2.8808
2.8576
2.8347
2.8115
2.7882
1.2493
1.2261
1.2047
1.1808
1.1570
Integral
4.0117
1.0000
12.062
16 15 14 13 12 11 10 9 8 7 6 5 4 3 2 1 0 −1 −2
(ppm)
